# Palladium Mesoionic Carbene Pre-catalyst for General Cross-Coupling Transformations in Deep Eutectic Solvents

**DOI:** 10.3389/fchem.2019.00700

**Published:** 2019-10-23

**Authors:** Xavier Marset, Beatriz Saavedra, Nerea González-Gallardo, Alexander Beaton, Martín M. León, Raúl Luna, Diego J. Ramón, Gabriela Guillena

**Affiliations:** Departamento de Química Orgánica, Facultad de Ciencias, Instituto de Síntesis Orgánica (ISO), Universidad de Alicante, Alicante, Spain

**Keywords:** mesoionic carbene, palladium, Deep Eutectic Solvent, sustainability, cross-coupling reaction

## Abstract

A strong σ-donor mesoionic carbene ligand has been synthesized and applied to four different palladium-catalyzed cross-coupling transformations, proving the catalyst/medium compatibility and the increased activity of this system over previous reports in Deep Eutectic Solvent medium. Some cross-coupling processes could be carried out at room temperature and using aryl chlorides as starting materials. The possible implementation of multistep synthesis in eutectic mixtures has also been explored. The presence of palladium nanoparticles in the reaction media has been evaluated and correlated to the observed activity.

## Introduction

Organic Chemistry is, by definition, the study of carbon containing compounds. Thus, reactions that involve C-C bond formation have a great impact in this discipline. Due to this fact, C-C bond forming cross-coupling reactions have arisen as a main tool in Organic Synthesis. The importance and high applicability of this kind of reactions has been proven with the award of several Nobel Prizes in this area over the last years (Johansson Seechurn et al., 2012). Several metallic catalysts have been applied to these reactions, with palladium derived catalysts being the most employed (Biffis et al., 2018). This is the case for reactions as important as Suzuki (1985), Sonogashira (2002), Heck (1982), or Hiyama couplings (Hiyama, 2002).

Due to their enormous relevance, these reactions are performed nowadays in ton-scale, affording products of great importance in areas such as pharmaceuticals, fine chemical, and agrochemical industries (Torborg and Beller, 2009). For this reason, these reactions have been largely studied over the last decades and efficient catalysts have been developed. However, most of these cross-coupling reactions are performed using traditional volatile organic compounds (VOCs) as solvents. These solvents are usually flammable, toxic and persistent in the atmosphere, and their replacement by safer alternatives following Green Chemistry principles is highly desired (Anastas and Warner, 1998).

Some reports of cross-coupling reactions in neoteric solvents have been recently described, including the use of water (Chatterjee and Ward, 2016), γ-valerolactone (Ismalaj et al., 2014), glycerol (Reina et al., 2018), or supercritical carbon dioxide (Feng et al., 2010) as solvents. Recently, Deep Eutectic Solvents (DESs) have been explored as reaction media for cross-coupling reactions, with some advantages over the aforementioned solvents (García-Álvarez, 2014).

DESs are mixtures formed combining two or more components that display, usually strong hydrogen-bond interactions, affording a mixture with a melting point much lower than each of their individual components (Abbott et al., 2004). These solvents have a negligible vapor pressure and are non-flammable. Most of the components that are used to produce these eutectic mixtures are naturally-occurring, biodegradable, and biorenewable. Furthermore, there are supposed to exist more than one million possible mixtures (Hammond et al., 2017), affording the possibility of designing a solvent for each reaction. Due to their high versatility, tunability, and sustainability, their use as medium to carry out metal-catalyzed organic transformations has attracted the interest of organic chemists.

**Figure 1:**
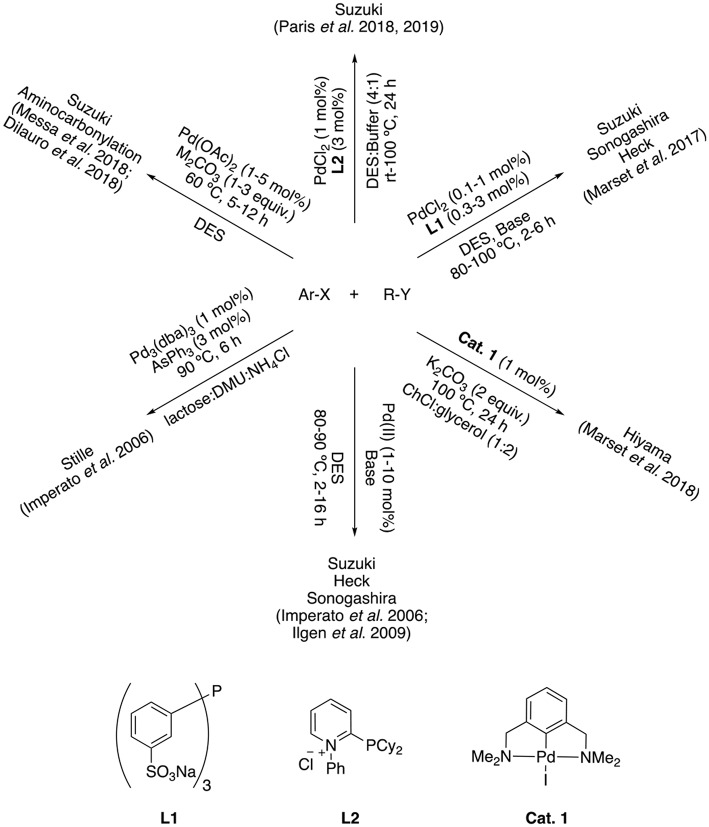
Cross-coupling reactions performed in DESs.

With DESs rising their popularity, it did not take long for cross coupling reactions to be tested in this novel reaction medium. Therefore, cross-coupling reactions in DESs have gained importance over the last few years as it is proven by the growing number of published articles in this research area. However, more efficient systems with broader applicability still have to be developed (Figure 1).

In order to improve the activity of a catalytic system, ligands are usually employed to tune the pre-catalyst properties. Considering the palladium-catalyzed cross coupling reactions in DES, first reports of the Suzuki (Imperato et al., 2006a), Sonogashira and Heck couplings (Ilgen and König, 2009) were performed in a ligand-free fashion, requiring high palladium loadings (up to 10 mol%). The Suzuki reaction in DES have been accomplished also with lower Pd(OAc)_2_ loadings by employing aryltrifluoroborates instead of boronic acids (Dilauro et al., 2018) as well as in the aminocarbonylation of aryl iodides (Messa et al., 2018). Our group have also contributed to this research field with the Hiyama reaction performed with a robust NCN-Pd pincer catalyst in ChCl:glycerol (Marset et al., 2018). However, the use of ionic phosphines seems to increase the compatibility of the palladium pre-catalysts with the reaction media, as it has been proven by the use of anionic TPPTS ligand, which was followed by enzymatic catalyzed reactions (Paris et al., 2018, 2019). Thus, the use of designed ligands seems mandatory for enhancing the activity of palladium catalysts in DES media. Early reports proved that arsines were better ligands than traditional phosphines when the Stille coupling was performed in DES (Imperato et al., 2006b). Our group also showed that α-cationic phosphine ligands greatly enhanced the activity of PdCl_2_ in the Suzuki, Heck and Sonogashira couplings performed in eutectic mixtures, while traditional non-ionic phosphines were not adequate (Marset et al., 2017). Those α-cationic phosphines have lower σ-donor ability compared to traditional phosphines, while they π-acceptor character is enhanced. This property could improve the catalyst activity in elementary steps such as reductive elimination or coordination of substrates to the metal center due to its electron-poor character (Alcarazo, 2016; Nicholls and Alcarazo, 2019).

The catalytic activity of metal complexes in cross-coupling reactions is often improved by the use of ligands with strong electron-donating properties. For this reason, in an attempt to increase the activity of DES-compatible catalysts the synthesis of a N-heterocyclic carbene (NHC)-Pd complex was carried out. NHC ligands, as well as phosphines, are strong σ-donors and weak π-acceptors. However, abnormal N-heterocyclic carbenes (aNHC) are reported to be even stronger σ-donors ligands (Crabtree, 2013). This stronger σ-donors ability increase the electron density at the metal center, enhancing the propensity of the metal to undergo oxidative addition (Krueger and Albrecht, 2011; Donnelly et al., 2013; Albrecht, 2014). To the best of our knowledge, the use of aNHC-Pd complexes in DESs as reaction medium has not been explored so far. For this reason, we aimed to synthetize an abnormal NHC-Pd complex and test it in different cross coupling reactions performed in DES.

## Results and Discussion

**Scheme 1:**
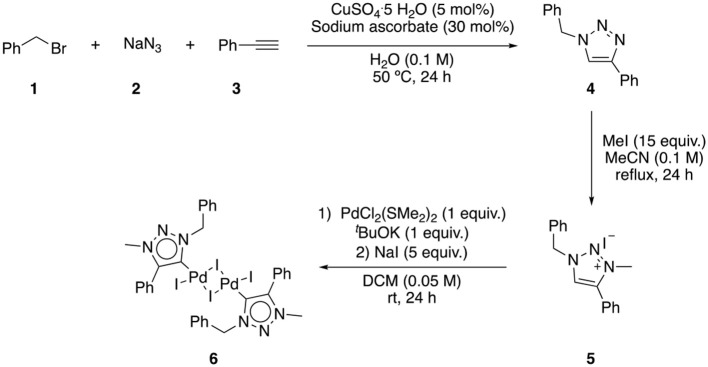
Synthesis of palladium complex **6**.

Complex **6** was prepared according to literature procedures (Scheme 1). The first step involved a multicomponent click-type reaction to afford triazole **4**, which was subjected to an alkylation reaction, yielding the salt **5**. Ligand **5** was then, deprotonated with potassium *tert*-butoxide and treated with a palladium precursor. Finally, an excess of NaI was added to afford complex **6**.

Once the catalyst was prepared, its ability to catalyze the Suzuki coupling was tested (Supplementary Table S1). The reaction between 4′-bromoacetophenone and phenylboronic acid was chosen as model reaction. A 40% yield of product **9a** was obtained after 3 h using water as solvent, while only 12% yield was observed using ChCl:ethylene glycol (1:2) as reaction media. Water is, obviously, a non-toxic and inexpensive solvent, but dealing with aqueous residues is not an easy task. For this reason, the use of DESs as reaction media has an increased value. It is worth to mention that eutectic mixtures can maintain their structure even when small amounts of water are added to the mixture, while their physical properties are modulated (Hammond et al., 2017). For this reason, it was decided to add certain amounts of water to the reaction DES mixture, observing an increase in the reaction rate when 1 to 10 equivalents of water were added, while adding 20 equivalents decreased the reaction yield (Supplementary Table S1, entries 2–9). Then other solvents (entries 10–20) and bases (entries 21–27) were tested, obtaining the best results with ChCl:ethylene glycol, 10 equivalents of water and 1.5 equivalents of K_2_CO_3_, as base (99% yield). With the optimal conditions in hand, the scope of aryl halides was evaluated (Table 1). The reaction worked with moderate to excellent yields for aryl bromides bearing electro-withdrawing and electro-donating functional groups at different positions. Electron-poor aryl chlorides were also efficiently coupled with phenylboronic acids with moderate to good yields at room temperature in 3 h.

**Table 1:** Scope of Suzuki reaction^[a]^.

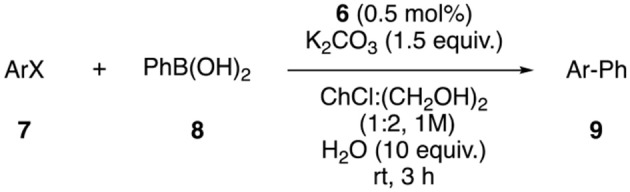				
**Entry**	**Ar**	**X**	**Product**	**Yield (%)^[b]^**
1	4-(HO_2_C)C_6_H_4_	Br	**9a**	90
2	4-(HO_2_C)C_6_H_4_	Cl	**9a**	60^[c]^
3	4-(CHO)C_6_H_4_	Br	**9b**	95
4	4-(CHO)C_6_H_4_	Cl	**9b**	60^[c]^
5	4-(MeCO)C_6_H_4_	Br	**9c**	99
6	4-(MeCO)C_6_H_4_	Cl	**9c**	50^[c]^
7	4-(O_2_N)C_6_H_4_	Br	**9d**	95
8	4-(O_2_N)C_6_H_4_	Cl	**9d**	90^[c]^
9	4-(NC)C_6_H_4_	Br	**9e**	98
10	4-(NC)C_6_H_4_	Cl	**9e**	95^[c]^
11	C_6_H_5_	Br	**9f**	60
12	4-FC_6_H_4_	Br	**9g**	79
13	4-MeC_6_H_4_	Br	**9h**	49
14	1-naphtyl	Br	**9i**	80
15	1-naphtyl	Cl	**9i**	43^[c]^
16	4-(HO)C_6_H_4_	Br	**9j**	50
17	4-(HO)C_6_H_4_	Cl	**9j**	20^[c]^
18	4-(MeO)C_6_H_4_	Br	**9k**	90
19	4-(MeO)C_6_H_4_	Cl	**9k**	50^[c]^

[a] *Reaction conditions: Aryl halide (0.2 mmol), phenyl boronic acid (0.21 mmol), K_2_CO_3_ (0.3 mmol), complex **6** (0.001 mmol), and water (36 μL) in 0.2 mL of ChCl:ethylene glycol (1:2) were stirred at rt for 3 h*.

[b] *Isolated yield after flash chromatography*.

[c] *Reaction time 10 h*.

On the other hand, aryl boronic acids bearing different substituents, such as alkyl, hydroxyl or halide groups, were coupled with 4′-bromoacetophenone with good to excellent yields (Table 2).

**Table 2:** Scope of arylboronic acids^[a]^.

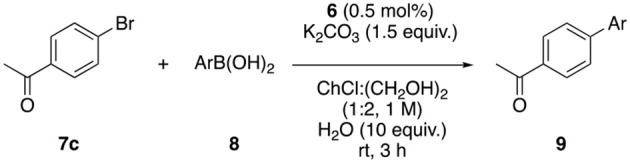			
**Entry**	**Ar**	**Product**	**Yield (%)^[b]^**
1	3-MeC_6_H_4_	**9l**	87
2	4-(HO)C_6_H_4_	**9m**	75
3	4-ClC_6_H_4_	**9n**	95
4	4-FC_6_H_4_	**9o**	84

[a] *Reaction conditions: 4′-bromoacetophenone (0.2 mmol), aryl boronic acid (0.21 mmol), K_2_CO_3_ (0.3 mmol), complex **6** (0.001 mmol), and water (36 μL) in 0.2 mL of ChCl:ethylene glycol (1:2) were stirred at rt for 3 h*.

[b] *Isolated yield after flash chromatography*.

Since recent reports have proven the compatibility of DES with the use of organometallic reagents such as organolithium or organomagnesium compounds (Vidal et al., 2014, 2016; García-Álvarez et al., 2015, 2018; Rodríguez-Álvarez et al., 2018), we decided to test the cross-coupling products **9b** and **9c** as substrates for the addition of different organometallic species in ChCl:ethyleneglycol as solvent. Aldehyde **9b** could be reduced to the corresponding primary alcohol with NaBH_4_ (Table 3, entry 1) or coupled with *n*-BuLi (entry 4). Better results were obtained with ketone **9c**, observing the aforementioned transformations (entries 5 and 8), as well as the addition of Grignard reagents (entries 6–7). These results highlighted the possibility of implementing DESs in a broad spectrum of different synthetic steps for the preparation of any product.

**Table 3:** Organometallic addition to carbonyl derivatives in DES^[a]^.

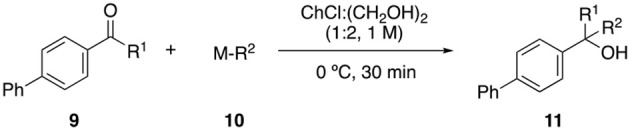					
	**M-R**^**2**^	**R**^**1**^	**R**^**2**^	**Product**	**Conv.^[b]^**
1	NaBH_4_	H	H	**11a**	10
2	*n*-BuLi	H	*n*-Bu	**11b**	25
3	NaBH_4_	Me	H	**11c**	10
4	H_2_C = CHCH_2_MgBr	Me	H_2_C = CHCH_2_	**11d**	15
5	*n*-BuLi	Me	*n*-Bu	**11e**	70

[a] *Reaction conditions: to a vigorously stirred solution of product **9b/9c** (0.2 mmol) in 0.3 mL of ChCl:ethylene glycol at 0°C, 0.5 mmol of the corresponding reagent **10** was added. The solution was stirred at rt for 30 min*.

[b] *Conversion determined by GC*.

To further prove the activity of the catalyst, other cross-coupling reactions were tested. The Sonogashira reaction was optimized with catalyst **6** in DESs as reaction media, obtaining the best results with AcChCl:urea (1:2) as solvent and ^i^PrNH_2_ as base in 2 h at room temperature, using only 1 mol% of complex **6** (95% yield, Supplementary Table S2). Then, the scope of the reaction was evaluated, obtaining moderate to excellent yields for the coupling of different aryl iodides with phenylacetylene (Table 4). 4′-Bromoacetophenone and 4′-chloroacetophenone were also submitted to the reaction with phenylacetylene but they failed. Excellent yields were obtained for electron-poor aryl iodides, although the yield was lower for neutral or electron rich aryl halides. However, results were improved by increasing the reaction time or the reaction temperature from rt to 80°C, which proved the stability of the catalyst at higher temperatures.

**Table 4:** Scope of Sonogashira coupling^[a]^.

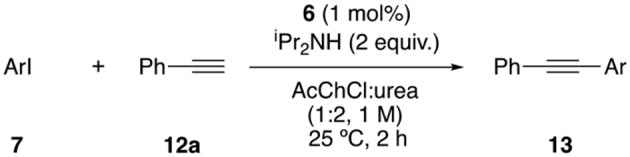			
**Entry**	**Ar**	**Product**	**Yield (%)^[b]^**
1	4-(MeCO)C_6_H_4_	**13a**	93
2	Ph	**13b**	56, (68)^[c]^, (81)^[d]^
3	4-MeC_6_H_4_	**13c**	36, (46)^[c]^, (22)^[d]^ (76)^[e]^
4	4-(O_2_N)C_6_H_4_	**13d**	82
5	2-MeC_6_H_4_	**13e**	26, (38)^[c]^, (31)^[d]^, (53)^[e]^
6	2-thienyl	**13f**	36, (38)^[c]^
7	4-FC_6_H_4_	**13g**	51, (62)^[c]^, (99)^[d]^
8	4-(MeO)C_6_H_4_	**13h**	28, (46)^[c]^, (30)^[d]^, (60)^[e]^

[a] *Reaction conditions: Aryl iodide (0.2 mmol), phenylacetylene (0.4 mmol), ^i^Pr_2_NH (0.4 mmol), complex **6** (0.002 mmol) in 0.2 mL of AcChCl:urea (1:2) were stirred at rt for 2 h*.

[b] *Isolated yield after flash chromatography*.

[c] *Reaction performed in 6 h*.

[d] *Reaction performed during 6 h at 80°C*.

[e] *Reaction performed during 24 h*.

The Heck coupling was also evaluated. As in the previous reaction, AcChCl:urea (1:2) proved to be the best solvent after a thoughtful optimization process (99% yield, Supplementary Table S3). Good to excellent results were obtained for the coupling of electron-poor aryl iodides with different substitution patterns with methylacrylate, using 1.5 equiv. of NaOAc as base at 120°C and only 1 mol% of complex **6** (Table 5, entries 1–4). In the case of using electron-rich starting materials, the reaction time was increased to 16 h, obtaining the corresponding cross-coupling products with good to excellent yields (entries 6–8). Also, in this case aryl bromides (4′-bromoacetophenone) and aryl chlorides (4′-chloroacetophenone) failed.

**Table 5:** Scope of Heck coupling^[a]^.

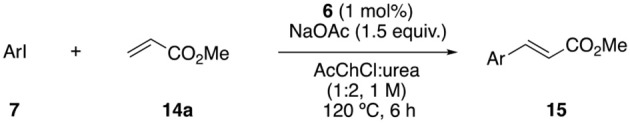			
**Entry**	**Ar**	**Product**	**Yield (%)^[b]^**
1	4-(O_2_N)C_6_H_4_	**15a**	99
2	4-(MeCO)C_6_H_4_	**15b**	95
3	4-(F_3_C)C_6_H_4_	**15c**	80
4	Ph	**15d**	72
5	1-naphthyl	**15e**	48
6	2-MeC_6_H_4_	**15f**	57^[c]^
7	4-MeC_6_H_4_	**15g**	91^[c]^
8	4-(MeO)C_6_H_4_	**15h**	78^[c]^

[a] *Reaction conditions: Aryl iodide (0.2 mmol), methyl acrylate (0.25 mmol), NaOAc (0.3 mmol), complex **6** (0.002 mmol) in 0.2 mL of AcChCl:urea (1:2) were stirred at 120°C for 6 h*.

[b] *Isolated yield after flash chromatography*.

[c] *Reaction performed during 16 h*.

The biaryl synthesis through cross-coupling reactions is usually performed under Suzuki conditions. Nevertheless, Hiyama reaction offers certain advantages from a sustainable and economic point of view, although organosilanes are typically less reactive than the corresponding boron counterparts. For this reason, it was decided to study the Hiyama reaction between aryl bromides and phenyltrimethoxysilane. The best result was obtained using ChCl:glycerol (1:2) as solvent, 1.5 equiv. of K_2_CO_3_ as base at 100°C for 24 h (70% yield, Supplementary Table S4). Good to excellent yields were observed for both, electron-poor (Table 6, entries 1–4) and electron-rich aryl bromides (entries 5–6). Furthermore, the reaction was compatible with heteroaryl bromides, affording the corresponding heterobiaryls in excellent yields (entries 7–9).

**Table 6:** Scope of Hiyama Reaction^[a]^.

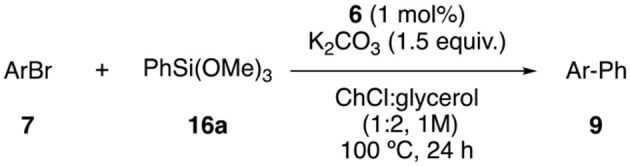			
**Entry**	**Ar**	**Product**	**Yield (%)^[b]^**
1	4-(MeCO)C_6_H_4_	**9c**	70
2	4-(O_2_N)C_6_H_4_	**9d**	88
3	C_6_H_5_	**9f**	95
4	4-FC_6_H_4_	**9g**	95
5	4-(HO)C_6_H_4_	**9j**	85
6	4-(MeO)C_6_H_4_	**9k**	67
7	3-pyridyl	**9p**	95
8	3-furyl	**9q**	88
9	2-thienyl	**9r**	88

[a] *Reaction conditions: Aryl bromide (0.5 mmol), PhSi(OMe)_3_ (0.75 mmol), K_2_CO_3_ (0.75 mmol), complex **6** (0.005 mmol) in 1 mL of ChCl:glycerol (1:2) were stirred at 100°C for 24 h*.

[b] *Isolated yield after flash chromatography*.

An attempt to reuse the catalyst and solvent by extracting the organic products with 2-MeTHF for the Suzuki and Hiyama reactions was performed. However, important drops in the reaction yields were observed in the second cycle (from 92 and 95% to 36 and 40%, respectively). Probably the poor activity of the recycled system could be explained by the formation of inactive palladium black, as well as the iodine poisoning of palladium nanoparticles (Cano et al., 2016).

In addition, with the aim of understanding better the catalytic process, a mercury poisoning test was performed. Thus, if the reactions were catalyzed by palladium nanoparticles (NPs), an important drop in the reaction yield should be noticed (Sigeev et al., 2015). The four cross-coupling reactions studied were analyzed again under optimal conditions but with the addition of 250 mol% of Hg(0). A complete inhibition of the catalytic activity was observed in the cases of the Suzuki, Sonogashira and Heck cross-coupling reactions. Surprisingly, no inhibition at all was observed in the case of the Hiyama coupling. A similar result was previously observed employing a Pd NCN-pincer in DES (Marset et al., 2018).

**Figure 2:**
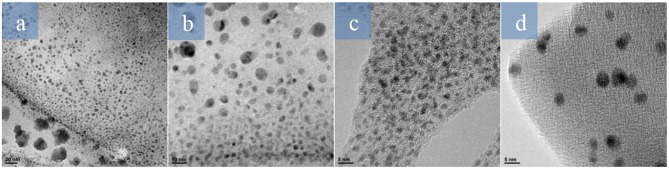
TEM images of the crude media after **(a)** Suzuki, **(b)** Sonogashira, **(c)** Heck, and **(d)** Hiyama cross-coupling reactions.

Since the formation of palladium nanoparticles seemed to be vital for the outcome of the desired organic transformations, the crude materials of the standard reactions were analyzed by electron transmission microscopy (TEM) to characterize those NPs (Figure 2). The size distributions of the observed nanoparticles are summarized in Table 7. Similar results were obtained in the cases of Suzuki and Sonogashira reactions. Although the solvent and base employed were different for each transformation (Suzuki and Sonogashira), a similar size of the nanoparticles was observed in both cases. In the case of Heck reaction, the average size of the nanoparticles was smaller, probably due to the higher temperature of the reaction and the different composition of the mixture. On the other hand, bigger NPs were found in the crude material of the Hiyama reaction (performed at 100°C). However, the longer reaction time required for this transformation could favor the NP aggregation, which may also explain the recyclability issues.

**Table 7:** Size distribution of palladium nanoparticles.

**Reaction**	**Average length (nm)^[a]^**
Suzuki	6.1 ± 3.0
Sonogashira	6.7 ± 2.9
Heck	2.1 ± 0.4
Hiyama	8.7 ± 3.2

[a] *Average calculated from at least 200 NPs observed in HRTEM images*.

**Figure 3:**
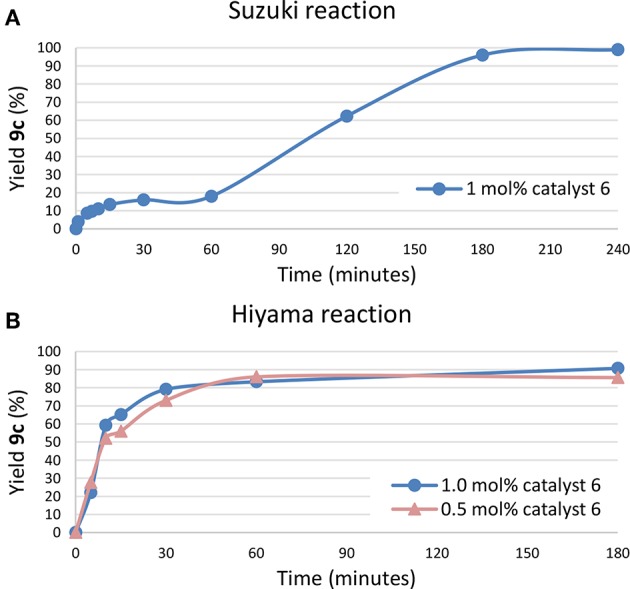
**(A)** Kinetic plot of the Suzuki cross-coupling reaction, **(B)** Kinect plot of the Hiyama cross-coupling reaction.

In order to better understanding the nature of the catalytic process kinetic studies and XPS analysis were performed (Figure 3). In the Suzuki kinetic plot an induction period was observed. This result seems to indicate that until palladium nanoparticles were not formed, the reaction did not occur. So, catalyst **6** plays a pre-catalyst role and palladium nanoparticles seems to be the real catalyst which is in consonance with the mercury test. However, in the Hiyama reaction any induction period was observed even decreasing the catalytic amount. Pre-formed palladium nanoparticles were employed to carry out Hiyama reaction under the optimized conditions but no catalysis was observed. So, this result seems to indicate that soluble palladium species with coordinated NHC ligands might be considered as the catalyst for the reaction through a Pd(II)-Pd(IV) catalytic process (Szulmanowicz et al., 2013; Modak et al., 2015; Mondal et al., 2018).

Also, XPS analysis (see Supplementary Material) shows that the oxidation state of the initial catalyst **6** changes completely to palladium(0) and palladium(II) oxide species, after the Suzuki reaction under the optimized conditions.

## Conclusion

The use of a mesoionic carbene ligand in palladium-catalyzed cross-coupling reactions has been evaluated for the first time in Deep Eutectic Solvents as reaction media, proving to be an excellent and general pre-catalyst (it decomposes to PdNPs) for many different cross-coupling reactions. The electronic properties of the reported palladium complex have allowed to carry out the Suzuki cross-coupling reaction in the aforementioned neoteric solvents at room temperature, even using aryl chlorides as reagents. Furthermore, the Sonogashira, Heck and Hiyama reactions have been also optimized and their scope evaluated, proving the great applicability of the mesoionic carbene-palladium complex. In addition, the reaction media is compatible with the use of organometallic reagents, demonstrating the possibility of implementing DESs in a broad spectrum of synthetic steps. The low catalyst loading, the sustainable properties of the employed solvents and the vast scope analyzed demonstrate that the use of this catalyst in combination with DESs as reaction media is an interesting protocol for carrying out any type of C_*sp*_2-C_*sp*_2_(*sp*)_ bond forming transformations. However, is still necessary to improve these protocols using DESs as solvents, to overcome the catalyst loading amount obtained in classical organic solvents.

## Data Availability Statement

All datasets for this study are included in the manuscript/Supplementary Material.

## Author Contributions

GG and DR contributed conception and design of the study. XM, BS, NG-G, AB, ML, and RL performed the synthetic experiments and characterization of products. XM, BS, and NG-G wrote the first draft of the manuscript. All authors contributed to manuscript revision, read, and approved the submitted version.

### Conflict of Interest

The authors declare that the research was conducted in the absence of any commercial or financial relationships that could be construed as a potential conflict of interest.
