# Supplementary material for: Palladium Mesoionic Carbene Pre-catalyst for General Cross-Coupling Transformations in Deep Eutectic Solvents
Source: Front Chem. 2019 Oct 23;7:700. doi: 10.3389/fchem.2019.00700 (PMC6819497; doi:10.3389/fchem.2019.00700)

Supplementary Material

1 Optimization tables

Supplementary Table S1. Optimization of the reaction conditions of the Suzuki coupling.[a]

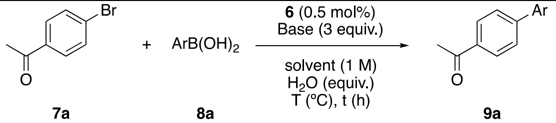

7a 8a 9a

Entry	Solvent	Base	t (h)	T (ºC)	Equiv. H_2O	Yield (%)[b]
1	H_2O	K_2CO_3	3	25	0	40
2	ChCl:ethylene glycol (1:2)	K_2CO_3	3	25	0	12
3	ChCl:ethylene glycol (1:2)	K_2CO_3	24	25	0	99
4	ChCl:ethylene glycol (1:2)	K_2CO_3	3	25	0	30
5	ChCl:ethylene glycol (1:2)	K_2CO_3	3	25	1	41
6	ChCl:ethylene glycol (1:2)	K_2CO_3	3	25	3	56
7	ChCl:ethylene glycol (1:2)	K_2CO_3	3	25	5	60
8	ChCl:ethylene glycol (1:2)	K_2CO_3	3	25	10	92
9	ChCl:ethylene glycol (1:2)	K_2CO_3	3	25	20	10
10	ChCl:glycerol (1:2)	K_2CO_3	3	25	10	62
11	ChCl:urea (1:2)	K_2CO_3	3	25	10	10
12	ChCl:urea (1:2)	-	3	25	10	10
13	ChCl:DMU (1:2)	K_2CO_3	3	25	10	10
14	ChCl:DMU (1:2)	-	3	25	10	10
15	ChCl:resorcinol (1:2)	K_2CO_3	3	25	10	44
16	Ethylene glycol	K_2CO_3	3	25	10	36
17	Glycerol	K_2CO_3	3	25	10	12
18	Ethanol	K_2CO_3	3	25	10	60
19	DMF	K_2CO_3	3	25	10	67
20	Toluene	K_2CO_3	3	25	10	27
21	ChCl:ethylene glycol (1:2)	K_2CO_3	3	25	10	90
22	ChCl:ethylene glycol (1:2)	K_2CO_3	3	40	10	40
23	ChCl:ethylene glycol (1:2)	KF	3	25	10	50
24	ChCl:ethylene glycol (1:2)	NaOH	3	25	10	60
25	ChCl:ethylene glycol (1:2)	Cs_2CO_3	3	25	10	95
26	ChCl:ethylene glycol (1:2)	$^{i}Pr_2NH$	3	25	10	0
27	ChCl:ethylene glycol (1:2)	-	3	25	10	0
28	ChCl:ethylene glycol (1:2)	K_2CO_3	3	25	10	90[c]
29	ChCl:ethylene glycol (1:2)	K_2CO_3	3	25	10	90[d]

[a] Reaction conditions: aryl bromide (0.2 mmol), K_2CO_3 (0.4 mmol), phenylboronic acid (0.25 mmol) and complex 6 (0.001 mmol) in 0.2 mL of solvent.[b] Yield determined by GC using tridecane as internal standard. [c] 0.3 mmol of K_2CO_3 were used. [d] 0.3 mmol of K_2CO_3 and 0.21 mmol of $PhB(OH)_2$ were used.

Supplementary Table S2. Optimization of Sonogashira coupling.[a]

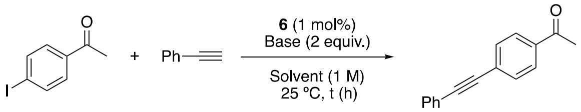

Entry	Base	t (h)	Solvent	Yield (%)[b]
1	$^iPrNH_2$	3	ChCl:ethylene glycol (1:2)	91
2	$^iPrNH_2$	3	ChCl:glycerol (1:2)	86
3	$^iPrNH_2$	3	AcChCl:urea (1:2)	93
4	$^iPrNH_2$	3	Decanoic acid:menthol (1:2)	0
5	$^iPrNH_2$	3	Decanoic acid:TBAB (2:1)	50
6	Na_2CO_3	3	AcChCl:urea (1:2)	5
7	$NaHCO_3$	3	AcChCl:urea (1:2)	1
8	K_2CO_3	3	AcChCl:urea (1:2)	75
9	NaOAc	3	AcChCl:urea (1:2)	39
10	K_3PO_4	3	AcChCl:urea (1:2)	56
11	$^iPrNH_2$	2	AcChCl:urea (1:2)	95
12	$^iPrNH_2$	1	AcChCl:urea (1:2)	34

[a] Reaction conditions: Aryl iodide (0.2 mmol), phenylacetylene (0.4 mmol), base (0.4 mmol), complex **6** (0.002 mmol) in 0.2 mL solvent were stirred at rt. [b] Yield determined by GC using tridecane as internal standard.

Supplementary Table S3. Optimization of Heck coupling.[a]

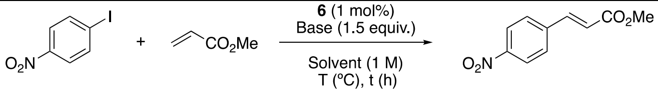

Entry	Base	T (ºC)	t (h)	Solvent	Yield (%)[b]
1	NaOAc	120	6	ChCl:ethylene glycol (1:2)	90
2	NaOAc	80	6	ChCl:ethylene glycol (1:2)	53
3	NaOAc	25	24	ChCl:ethylene glycol (1:2)	0
4	NaOAc	120	6	AcChCl:urea (1:2)	<99
5	NaOAc	120	6	ChCl:glycerol (1:2)	52
6	NaOAc	120	6	ChCl:urea (1:2)	0
7	NaOAc	120	6	AcChCl:acetamide (1:2)	59
8	NaOAc	120	6	Decanoic acid:menthol (1:2)	0
9	NaOAc	120	6	Decanoic acid:TBAB (2:1)	45
10	Na_2CO_3	120	6	AcChCl:urea (1:2)	0
11	K_2CO_3	120	6	AcChCl:urea (1:2)	19
12	K_3PO_4	120	6	AcChCl:urea (1:2)	0
13	$NaHCO_3$	120	6	AcChCl:urea (1:2)	0
14	NaOAc	120	3	AcChCl:urea (1:2)	40
15	NaOAc	120	5	AcChCl:urea (1:2)	58

[a] Reaction conditions: Aryl iodide (0.2 mmol), methyl acrylate (0.25 mmol), base (0.3 mmol), complex **6** (0.002 mmol) in 0.2 mL of solvent. [b] Yield determined by GC using tridecane as internal standard.

Supplementary Table S4. Optimization of Hiyama coupling.[a]

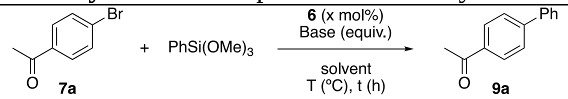

Entry	Catalyst (mol%)	Base (equiv.)	T (ºC)	t (h)	Solvent	Yield (%)[b]
1	1	K_2CO_3 (2)	100	24	ChCl:glycerol (1:2)	73
2	1	K_2CO_3 (2)	100	24	ChCl:ethyleneglycol (1:2)	12
3	1	K_2CO_3 (2)	100	24	ChCl:urea (1:2)	0
4	1	K_2CO_3 (2)	100	24	DecA:TBAB (1:2)	0
5	1	K_2CO_3 (2)	100	24	AcChCl:urea (1:2)	0
6	1	K_2CO_3 (2)	100	24	AcChCl:acetamide (1:2)	0
7	1	Na_2CO_3 (2)	100	24	ChCl:glycerol (1:2)	55
8	1	$NaHCO_3$ (2)	100	24	ChCl:glycerol (1:2)	30
9	1	K_3PO_4 (2)	100	24	ChCl:glycerol (1:2)	24
10	1	K_2CO_3 (1.5)	100	24	ChCl:glycerol (1:2)	70
11	1	K_2CO_3 (1)	100	24	ChCl:glycerol (1:2)	53
12	1	K_2CO_3 (2.5)	100	24	ChCl:glycerol (1:2)	38
13	1	K_2CO_3 (1.5)	100	6	ChCl:glycerol (1:2)	54
14	1	K_2CO_3 (1.5)	100	16	ChCl:glycerol (1:2)	60
15	1.5	K_2CO_3 (1.5)	100	24	ChCl:glycerol (1:2)	71
16	0.7	K_2CO_3 (1.5)	100	24	ChCl:glycerol (1:2)	35
17	1	K_2CO_3 (1.5)	50	24	ChCl:glycerol (1:2)	55
18	-	K_2CO_3 (1.5)	100	24	ChCl:glycerol (1:2)	-
19	1	-	100	24	ChCl:glycerol (1:2)	-

[a] Reaction conditions: Aryl bromide (0.5 mmol), $PhSi(OMe)_3$ (0.75 mmol), base (equiv.) in 1 mL of solvent. [b] Yield determined by GC using tridecane as internal standard.

2 XPS analysis

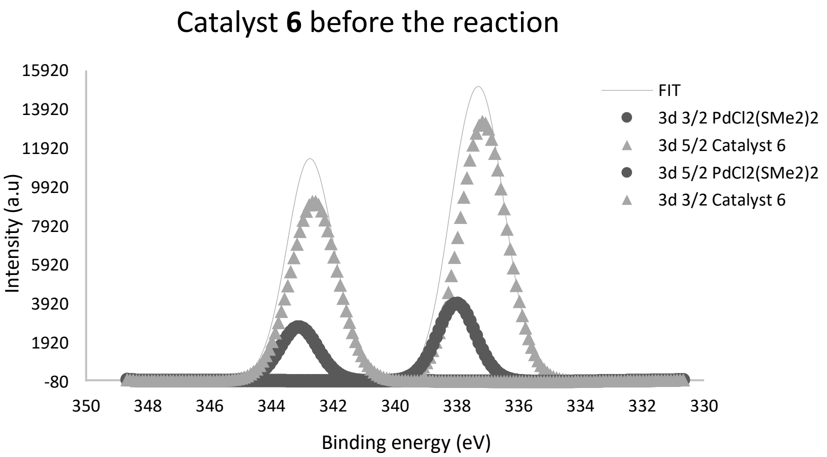

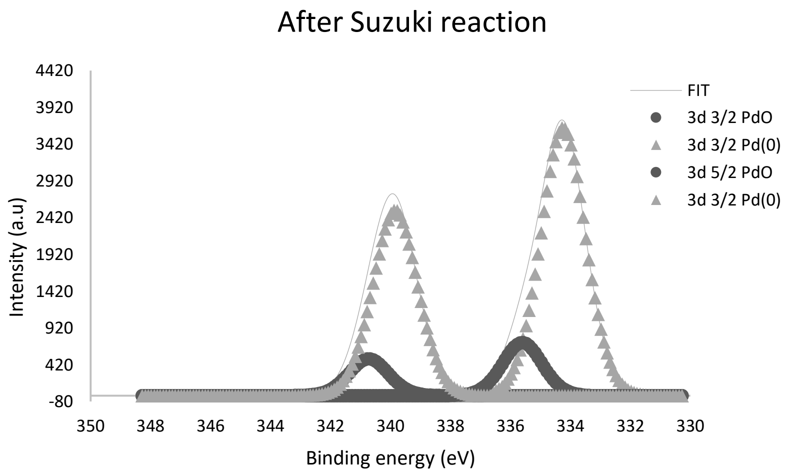
After Suzuki reaction
4420
3920
3420
2920
2420
1920
1420
920
420
-80
Intensity (a.u)
350
348
346
344
342
340
338
336
334
332
330
Binding energy (eV)
FIT
3d 3/2 PdO
3d 3/2 Pd(0)
3d 5/2 PdO
3d 3/2 Pd(0)

3 Characterization data.

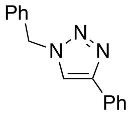

1-benzyl-4-phenyl-1*H*-1,2,3-triazole (4):(Sharghi et al., 2009) White solid; R_f = 0.57 (hexane/ethyl acetate: 1/1); m.p. 124-126 °C; t_r = 18.67; ^{1}H NMR (300 MHz, $CDCl_3$): δ = 7.80 (d, J = 7.3 Hz, 2H, ArH), 7.68 (s, 1H, CH-N), 7.45-7.25 (m, 8H, ArH), 5.55 (s, 2H, CH_2Ph); ^{13}C NMR (101 MHz, $CDCl_3$): δ = 148.3, 134.8, 130.6, 129.2 (2C), 128.9 (2C), 128.8, 128.3, 128.2 (2C), 125.8 (2C), 119.7, 54.3; IR (ATR): ν = 1450, 1223, 767 cm^{-1}; MS (EI) m/z (%): 235 (M^+, 26), 207 (74), 206 (73), 180 (11), 116 (100), 106 (12), 105 (16), 104 (24), 91(80), 89 (26), 77 (20), 65 (14).

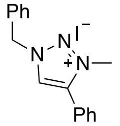

1-benzyl-3-methyl-4-phenyl-1*H*-1,2,3-triazolium chloride (5):(Mathew et al., 2008) Brown solid; R_f = 0.43 (ethyl acetate/methanol: 2/1); m.p. 127-129 °C; ^{1}H NMR (300 MHz, $CDCl_3$): δ = 9.42 (s, 1H, CH-N), 7.80-7.35 (m, 10H, ArH), 6.04 (s, 2H, CH_2Ph), 4.32 (s, 3H, NCH_3); ^{13}C NMR (101 MHz, $CDCl_3$): δ = 143.1, 132.1, 131.3, 130.1 (2C), 130.0, 129.8 (2C), 129.7 (2C), 129.5 (2C), 129.4, 121.7, 57.7, 39.6; IR (ATR): ν = 3038, 1157, 764 cm^{-1}.

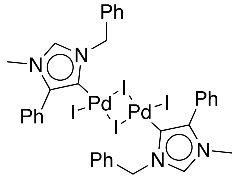

Complex (6):(Mathew et al., 2008) Brown solid; R_f = 0.57 (ethyl acetate/methanol: 2/1); m.p. 140 °C (decompose); ^{1}H NMR (300 MHz, DMSO-d_6): δ = 8.00-7.95 (m, 4H, ArH), 7.65-7.55 (m, 10H, ArH), 7.45-7.35 (m, 6H, ArH), 5.90 (s, 4H, 2xCH_2Ph), 4.04 (s, 6H, 2xNCH_3); ^{13}C NMR (101 MHz, DMSO-d_6): δ = 142.9, 133.8, 129.9 (2C), 129.7, 129.6 (2C), 129.3, 128.5 (2C), 128.4 (2C), 127.0, 58.7, 38.2 (C_{trz}-Pd not observerd); IR (ATR): ν = 3028, 1323, 1078, 771 cm^{-1}.

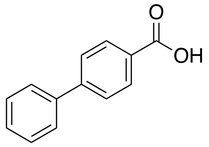

1-([1,1'-biphenyl]-4-carboxylic acid (9a):(Bunda et al., 2018) White solid; R_f = 0.47 (hexane/ethyl acetate: 1/1); m.p. 225-227 °C; t_r = 15.65; ^{1}H NMR (300 MHz, DMSO-d_6): δ = 12.96 (s, 1H, CO_2H), 8.10-7.95 (m, 2H, ArH), 7.85-7.80 (m, 2H, ArH), 7.80-7.70 (m, 2H, ArH), 7.55-7.45 (m, 2H, ArH), 7.45-7.40 (m, 1H, ArH); ^{13}C NMR (101 MHz, $CDCl_3$): δ = 167.1, 144.3, 139.0, 129.9 (2C), 129.6, 129.0 (2C), 128.2, 126.9 (2C), 126.8 (2C); IR (ATR): ν = 3346, 2918, 1675, 1607, 1287 cm^{-1}; MS (EI) m/z (%): 198 (M^+, 19), 190 (84), 177 (48), 152 (14), 96 (100), 78 (55).

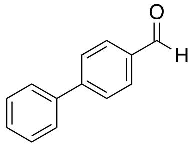

1-([1,1'-biphenyl]-4-carbaldehyde (9b): (Karimi et al., 2019) White solid; R_f = 0.47 (hexane/ethyl acetate: 4/1); m.p. 59-61 °C; t_r = 14.40; ^{1}H NMR (300 MHz, $CDCl_3$): δ = 10.06 (s, 1H, CHO), 7.95 (d, J = 8.2 Hz, 2H, ArH), 7.75 (d, J = 8.2 Hz, 2H, ArH), 7.70-7.60 (m, 2H, ArH), 7.55-7.40 (m, 3H, ArH); ^{13}C NMR (101 MHz, $CDCl_3$): δ = 192.0, 147.2, 139.8, 135.3, 130.3 (2C), 129.1 (2C), 128.6, 127.8 (2C), 127.4 (2C); IR (ATR): ν = 2839, 2743, 1696, 1601 cm^{-1}; MS (EI) m/z (%): 183 (M^+ + 1, 13), 182 (M^+, 99), 181 (100), 153 (33), 152 (59), 151 (17), 76 (13).

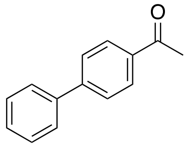

1-([1,1'-biphenyl]-4-yl)ethanone (9c):(Fairlamb et al., 2004) Orange solid; R_f = 0.50 (hexane/ethyl acetate: 4/1); m.p. 109-113 °C; t_r = 13.7; ^{1}H NMR (300 MHz, $CDCl_3$): δ = 8.04 (dt, J = 8.5, 1.9 Hz, 2H, ArH), 7.70-7.60 (dt, J = 8.5, 1.9 Hz, 4H, ArH), 7.50-7.40 (m, 3H, ArH), 2.63 (s, 3H, $COCH_3$); ^{13}C NMR (75 MHz, $CDCl_3$): δ = 197.9, 145.9, 140.0, 135.9, 129.1 (2C), 129.0 (2C), 128.3 (2C), 127.4 (2C), 127.3, 26.8; IR (ATR): ν = 3082, 1676, 1599, 721 cm^{-1}; MS (EI) m/z (%): 196 (M^+, 53), 182 (14), 181 (100), 153 (34), 152 (49), 151 (14), 76 (10).

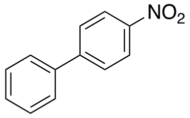

4-nitro-1,1'-biphenyl (9d):(Fairlamb et al., 2004) Yellow solid; R_f = 0.67 (hexane/ethyl acetate: 4/1); m.p. 98-100 °C; t_r = 13.8; ^{1}H NMR (300 MHz, $CDCl_3$): δ = 8.30 (d, J = 9.0 Hz, 2H, ArH), 7.74 (d, J = 9.0 Hz, 2H, ArH), 7.65-7.60 (m, 2H, ArH), 7.55-7.40 (m, 3H, ArH); ^{13}C NMR (75 MHz, $CDCl_3$): δ = 147.8, 147.2, 138.9, 129.3 (2C), 129.1, 127.9 (2C), 127.5

(2C), 124.3 (2C); IR (ATR): ν = 2921, 1595, 1512, 1340, 773 cm^{-1}; MS (EI) *m/z* (%): 200 (M^{+}+1, 14), 199 (M^{+}, 100), 169 (31), 153 (24), 152 (79), 151 (24), 141 (21).

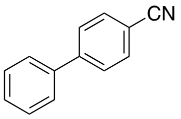

[1,1'-biphenyl]-4-carbonitrile (9e):(Stevens et al., 2005) White solid; R_f = 0.53 (hexane/ethyl acetate: 4/1); m.p. 69-71 °C; t_r = 14.46; ^{1}H NMR (300 MHz, $CDCl_3$): δ = 7.75-7.65 (m, 4H, ArH), 7.60-7.55 (m, 2H, ArH), 7.50-7.40 (m, 3H, ArH); ^{13}C NMR (75 MHz, $CDCl_3$): δ = 145.8, 139.3, 132.7 (2C), 129.3 (2C), 128.8, 127.9 (2C), 127.4 (2C), 119.1, 111.1; IR (ATR): ν = 2921, 2851, 2224, 1482 cm^{-1}; MS (EI) *m/z* (%): 180 (M^{+}+1, 32), 179 (M^{+}, 100), 178 (53), 177 (19), 152 (14), 151 (25).

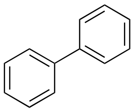

1,1'-biphenyl (9f):(Bandari et al., 2010) White solid; R_f = 0.53 (hexane); m.p. 68-70 °C; t_r = 10.0; ^{1}H NMR (300 MHz, $CDCl_3$): δ = 7.70-7.60 (m, 4H, ArH), 7.55-7.45 (m, 4H, ArH), 7.45-7.35 (m, 2H, ArH); ^{13}C NMR (75 MHz, $CDCl_3$): δ = 141.4 (2C), 128.9 (4C), 127.4 (2C), 127.3 (4C); IR (ATR): ν = 3033, 1477, 725 cm^{-1}; MS (EI) *m/z* (%): 155 (M^{+}+ 1, 14), 154 (M^{+}, 100), 153 (40), 152 (26), 76 (13).

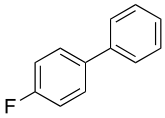

4-fluoro-1,1'-biphenyl (9g):(Iranpoor et al., 2016) White solid; R_f = 0.53 (hexane); m.p. 71-73 °C; t_r = 10.0; ^{1}H NMR (300 MHz, $CDCl_3$): δ = 7.60-7.55 (m, 4H, ArH), 7.50-7.45 (m, 2H, ArH), 7.40-7.35 (m, 1H, ArH), 7.20-7.10 (m, 2H, ArH); ^{13}C NMR (75 MHz, $CDCl_3$): δ = 162.6 (d, *J* = 246.3 Hz), 140.4, 137.5 (d, *J* = 3.2 Hz), 128.9 (2C), 128.8 (d, *J* = 8.0 Hz, 2C), 127.4, 127.2 (2C), 115.7 (d, *J* = 21.4 Hz, 2C); IR (ATR): ν = 3062, 1231, 756 cm^{-1}; MS (EI) *m/z* (%): 172 (M^{+}, 100), 171 (M^{+}-1, 35), 170 (25).

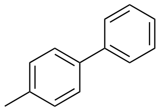

4-methyl-1,1'-biphenyl (9h):(Bandari et al., 2010) White solid; R_f = 0.73 (hexane); m.p. 43-45 °C; t_r = 10.9; ^{1}H NMR (300 MHz, $CDCl_3$): δ = 7.57 (ddd, *J* = 7.0, 4.1, 2.1 Hz, 2H, ArH), 7.55-7.45 (m, 2H, ArH), 7.45- 7.40 (m, 2H, ArH), 7.35-7.30 (m, 1H, ArH), 7.25-7.20 (m, 2H, ArH), 2.39 (s, 3H, $ArCH_3$); ^{13}C NMR (75 MHz, $CDCl_3$): δ = 141.3, 138.5, 137.2, 129.6 (2C), 128.9 (2C), 127.1 (5C), 21.2; IR (ATR): *ν* = 3059, 1481, 1379, 754 cm^{-1}; MS (EI) m/z (%): 169 (M^{+} + 1, 13), 168 (M^{+}, 100), 167 (66), 165 (25), 153 (15), 152 (20).

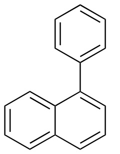

1-phenylnaphtalene (9i):(Zhang and Wang, 2006) White solid; R_f = 0.77 (hexane/ethyl acetate: 4/1); m.p. 43-45 °C; t_r = 17.86; ^{1}H NMR (300 MHz, $CDCl_3$): δ = 8.00-7.85 (m, 3H, ArH), 7.60-7.45 (m, 9H, ArH); ^{13}C NMR (75 MHz, $CDCl_3$): δ = 140.9, 140.4, 133.9, 131.8, 130.2, 128.4, 127.8, 127.4, 127.1, 126.2, 125.9, 125.5; IR (ATR): *ν* = 3055, 1395, 776 cm^{-1}; MS (EI) m/z (%): 169 (M^{+} + 1, 13), 168 (M^{+}, 100), 167 (66), 165 (25), 153 (15), 152 (20).

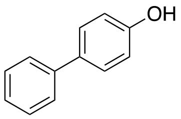

4-phenylphenol (9j):(Bai and Wang, 2008) White solid; R_f= 0.40 (Hexane/AcOEt: 4/1); m.p. 160-161 °C; t_r =14.60; ^{1}H NMR (300 MHz, $CDCl_3$): δ= 7.55-7.50 (m, 2H, ArH), 7.50-7.45 (m, 2H, ArH), 7.45-7.40 (m, 2H, ArH), 7.35-7.30 (m, 1H, ArH), 6.95-6.85 (m, 2H, ArH); ^{13}C NMR (75 MHz, $CDCl_3$): δ= 155.2, 140.9, 134.2, 128.9, 128.5, 126.8 (2C), 115.8; IR (ATR): *ν* = 3415, 3062, 1595, 1235, 756 cm^{-1}; MS (70 eV, EI): *m/z* (%): 170 (M^{+}, 100%), 141 (18), 115 (13).

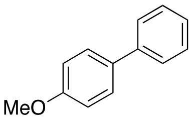

4-methoxy-1,1'-biphenyl (9k): (Iranpoor et al., 2016) White solid; R_f = 0.67 (hexane/ethyl acetate: 19/1); m.p. 76-78 °C; t_r = 12.3; ^{1}H NMR (300 MHz, $CDCl_3$): δ = 7.60-7.50 (m, 4H, ArH), 7.41 (t, *J* = 7.5 Hz, 2H, ArH), 7.30 (ddd, *J* = 7.3, 3.9, 1.3 Hz, 1H, ArH), 6.98 (d, *J* = 8.9 Hz, 2H, ArH), 3.85 (s, 3H, OCH_3); ^{13}C NMR (75 MHz, $CDCl_3$): δ = 159.3, 141.0, 133.9, 128.9 (2C), 128.3

(2C), 126.9 (2C), 126.8, 114.3 (2C), 55.5; IR (ATR): ν = 3070, 1604, 1523, 1271, 756 cm^{-1}; MS (EI) *m/z* (%): 185 (M^+ + 1, 14), 184 (M^+, 100), 169 (44), 141 (39), 115 (25).

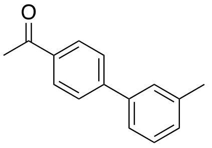

1-(3'-methyl-[1,1'-biphenyl]-4-yl)ethan-1-one (9l):(Li et al., 2014) White solid; R_f= 0.5 (Hexane/AcOEt 4/1); m.p. 89.0-90.3 ºC; t_r = 15.72 min; ^{1}H NMR (300 MHz, $CDCl_3$) δ= 8.09-7.90 (m, 2H, ArH), 7.73-7.61 (m, 2H, ArH), 7.43 (d, *J* = 7.3 Hz, 2H, ArH), 7.39-7.29 (m, 1H, ArH), 7.23 (dd, *J* = 7.7, 7.1 Hz, 1H, ArH), 2.64 (s, 3H, COCH_3), 2.44 (s, 3H, ArCH_3) ppm; ^{13}C NMR (101 MHz, $CDCl_3$) δ= 197.9, 146.1, 139.9, 138.7, 135.9, 129.1 (2C), 128.2, 127.4 (2C), 124.5, 26.8, 21.7 ppm; IR (ATR) ν = 2921, 2852, 1685, 1598, 1170 cm^{-1}; MS (70 eV, EI): m/z (%) 210 (M+, 48), 195 (100), 152 (29).

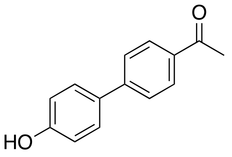

1-(4'-hydroxy-[1,1'-biphenyl]-4-yl)ethan-1-one (9m):(Monguchi et al., 2011) White solid; R_f= 0.2 (Hexane/AcOEt 2/1); m. p. 195-198 ºC; t_r = 17.32; ^{1}H NMR (300 MHz, $CDCl_3$) δ= 8.07-7.97 (m, 2H, ArH), 7.69-7.61 (m, 2H, ArH), 7.58-7.50 (m, 2H, ArH), 6.97-6.85 (m, 2H, ArH), 6.82-6.62 (m, 1H, -OH), 2.65 (s, 3H, COCH_3); ^{13}C NMR (101 MHz, MeOD) δ= 200.0, 159.1, 147.1, 136.0, 131.9, 129.9 (2C), 129.2 (2C), 127.2 (2C), 116.7, (2C), 26.5; IR (ATR) ν = 3286, 2922, 1653, 1598, 1295 cm^{-1}; MS (70 eV, EI): m/z (%) 212 (M^+, 63), 197 (100), 169 (11), 139 (15), 115 (12), 99 (4), 84 (7).

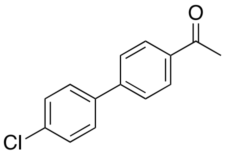

1-(4'-chloro-[1,1'-biphenyl]-4-yl)ethan-1-one (9n): (Kylmala et al., 2008) White solid; R_f = 0.43 (Hexane/AcOEt 4/1); m.p. 97-98 ºC; t_r = 15.95; ^{1}H NMR (300 MHz, $CDCl_3$) δ= 8.05-8.00 (m, 2H), 7.65-7.60 (m, 2H, ArH), 7.55-7.50 (m, 2H, ArH), 7.45-7.35 (m, 2H, ArH), 2.62 (s, 3H, CH_3); ^{13}C NMR (101 MHz, $CDCl_3$) δ = 197.6, 144.5, 138.4, 136.2, 134.5, 129.2 (2C), 129.1 (2C), 128.6 (2C), 127.1 (2C), 26.7; IR (ATR) ν = 1671, 1416, 1266 cm^{-1}; MS (70 eV, EI) m/z (%): 232 (M^+Cl^{37}, 17), 230 (M^+Cl^{35}, 50), 217 (33), 216 (15), 215 (100), 152 (62), 151 (14), 76 (12).

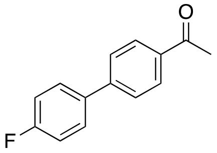

1-(4'-fluoro-[1,1'-biphenyl]-4-yl)ethan-1-one (9o):(Kienle and Knochel, 2010) Light brown solid; R_f = 0.5 (hexane/ethyl acetate: 4/1); m.p 95-97 ºC; t_r = 14.94; ^{1}H NMR (300 MHz, $CDCl_3$) δ= 8.05-7.99 (m, 2H, ArH), 7.67-7.61 (m, 2H, ArH), 7.60-7.55 (m, 2H, ArH), 7.19-7.10 (m, 2H, ArH), 2.63 (s, 3H, $COCH_3$); ^{13}C NMR (101 MHz, $CDCl_3$) δ= 197.8, 163.1 (d, *J* = 248.2 Hz) 144.8, 136.1 (d, *J* = 3.2 Hz), 136.0, 129.0 (d, *J* = 9.3 Hz), 127.2, 116.0 (d, *J* = 21.8 Hz), 26.8 ppm; IR (ATR) ν = 2923, 1681, 1599, 1494, 1161 cm^{-1}; MS (70 eV, EI): *m/z* (%) 214 (M^+, 53), 199 (100), 170 (48), 151 (5), 85 (9).

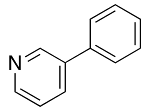

3-phenylpyridine (9p):(Ilie et al., 2017) Yellow oil; R_f = 0.47 (hexane/ethyl acetate: 1/1); t_r = 11.1; ^{1}H NMR (300 MHz, $CDCl_3$): δ = 8.85 (d, *J* = 1.7 Hz, 1H, ArH), 8.59 (dd, *J* = 4.9, 1.7 Hz, 1H, ArH), 7.91 (ddd, *J* = 7.9, 2.3, 1.7, 1H, ArH), 7.65-7.55 (m, 2H, ArH), 7.50-7.35 (m, 4H, ArH); ^{13}C NMR (75 MHz, $CDCl_3$): δ = 148.0, 147.9, 137.7, 137.0, 135.0, 129.3 (2C), 128.4, 127.3 (2C), 123.9; IR (ATR): ν = 3029, 1473, 710 cm^{-1}; MS (EI) *m/z* (%): 155 (M^+, 100), 154 (M^+-1, 52), 127 (13).

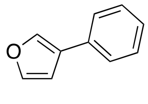

3-phenylfuran (9q):(Kumar Manian et al., 2010) White solid; R_f= 0.33 (hexane); m.p. 53-54 ºC; t_r =9.95; ^{1}H NMR (300 MHz, $CDCl_3$): δ= 7.75-7.70 (m, 1H, O-CH=C), 7.49 (dt, *J* = 7.3, 1.6 Hz, 3H, ArH), 7.40-7.35 (m, 2H, ArH), 7.30-7.20 (m, 1H, HC=C*H*-O) 6.71 (dd, *J* = 1.9, 0.9 Hz, 1H, *H*C=CH-O); ^{13}C NMR (75 MHz, $CDCl_3$): δ= 143.8, 138.6, 132.6, 128.9 (2C), 127.1, 126.6, 126.0 (2C), 109.0; IR (ATR): ν = 3127, 1605, 1510, 751 cm^{-1}; MS (70 eV, EI) *m/z* (%): 145 (M^+ + 1, 11), 144 (M^+, 100), 115 (84).

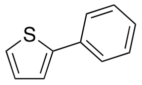

2-phenylthiophene (9r): (Lois et al., 2007) White solid; R_f = 0.4 (hexane); m. p. 30-32 ºC; t_r = 11.70; ^{1}H NMR (300 MHz, $CDCl_3$) δ= 7.65-7.60 (m, 2H, ArH), 7.50-7.35 (m, 2H, ArH), 7.35-

7.15 (m, 3H, ArH), 7.08 (dd, J = 5.1, 3.6 Hz, 1H) ppm; ^{13}C NMR (101 MHz, $CDCl_3$) δ= 144.6, 134.5, 129.0, 128.9, 128.1, 127.6, 127.3, 126.1, 124.9, 123.2 ppm; IR (ATR) ν = 2922, 1730, 1446, 1210, 826 cm^{-1}; MS (70 eV, EI) m/z (%) 160 (M^+, 100), 128 (8), 115 (25).

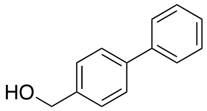

[1,1'-biphenyl]-4-ylmethanol (11a): (Furuyama et al., 2008) White solid; R_f = 0.2 (hexane/ethyl acetate: 4/1); m. p. 96-98 ºC; t_r = 14.92; ^{1}H NMR (300 MHz, $CDCl_3$) δ= 7.65-7.55 (m, 4H, ArH), 7.50-7.40 (m, 4H, ArH), 7.40-7.30 (m, 1H, ArH), 4.73 (s, 2H, CH_2OH), 1.78 (br s, 1H, CH_2OH);^{13}C NMR (75 MHz, $CDCl_3$) δ = 141.0, 140.8, 140.0, 128.9 (2C), 127.6 (2C), 127.5 (3C), 127.2 (2C); IR (ATR) ν = 3321, 1428, 1038, 751 cm^{-1}; MS (70 eV, EI) m/z (%): 184 (M^+, 88), 183 (37), 182 (97), 181 (100), 167 (19), 165 (24), 155 (60), 154 (60), 153 (51), 152 (85), 151 (24), 77 (20), 76 (17).

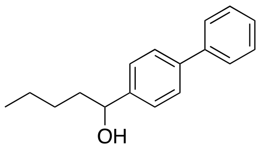

1-([1,1'-biphenyl]-4-yl)pentan-1-ol (11b): (Brekan et al., 2012) White solid; R_f = 0.40 (hexane/ethyl acetate: 4/1); m.p. 69-71 °C; t_r = 16.96; ^{1}H NMR (300 MHz, $CDCl_3$): δ = 7.65-7.60 (m, 4H, ArH),7.50-7.40 (m, 4H, ArH), 7.40-7.35 (m, 1H, ArH), 4.72 (dd, J = 7.2, 6.1 Hz, 1H, CHOH), 2.03 (br s, 1H, CHOH), 1.95-1.70 (m, 2H, CH_2CHOH), 1.50-1.25 (m, 4H, CH_3CH_2CH_2), 0.93 (t, J = 7.1 Hz, 3H, C$H_3$$CH_2$$CH_2$); ^{13}C NMR (101 MHz, $CDCl_3$): δ = 144.1, 141.0, 140.5, 128.9 (2C), 127.4, 127.3 (2C), 127.2 (2C), 126.5 (2C), 74.6, 38.9, 28.1, 22.8, 14.2; IR (ATR): ν = 3290, 2930, 2857, 1406, 1005 cm^{-1}; MS (EI) m/z (%): 240 (M^+, 6), 222 (32), 194 (17), 193 (100), 191 (14), 183 (60), 178 (82), 167 (14), 165 (33), 155 (20), 153 (11), 152 (21), 115 (12).

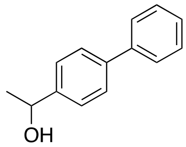

1-([1,1'-biphenyl]-4-yl)ethan-1-ol (11c): (Wang et al., 2007) White solid; R_f = 0.30 (hexane/ethyl acetate: 4/1); m.p. 57-59 °C; t_r = 15.02; ^{1}H NMR (300 MHz, $CDCl_3$): δ = 7.65-7.55 (m, 4H, ArH), 7.50-7.40 (m, 4H, ArH), 7.36 (ddd, J = 6.1, 3.8, 1.3 Hz, 1H, ArH), 4.96 (q, J = 6.4 Hz, 1H, CH_3CHOH), 2.11 (br s, 1H, CH_3CHOH), 1.55 (d, J = 6.4 Hz, 3H, CH_3CHOH); ^{13}C NMR (101 MHz, $CDCl_3$): δ = 144.9, 141.0, 140.5, 128.9 (2C), 127.4 (3C), 127.2 (2C), 126.0 (2C), 70.3, 25.2; IR (ATR): ν = 3313, 3040, 1343, 1085 cm^{-1}; MS (EI) m/z (%): 198 (M^+, 7), 183 (11), 181 (23), 180 (100), 179 (21), 178 (27), 165 (14), 155 (11), 152 (16).

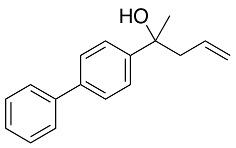

2-([1,1'-biphenyl]-4-yl)pent-4-en-2-ol (11d): (Li et al., 2011) yellowish oil; R_f = 0.30 (hexane/ethyl acetate: 4/1); t_r = 15.02; ^{1}H NMR (400 MHz, $CDCl_3$): δ = 7.65-7.55 (m, 4H, ArH), 7.55-7.50 (m, 2H, ArH), 7.50-7.40 (m, 2H, ArH), 7.40-7.30 (m, 1H, ArH), 5.68 (dddd, J = 16.7, 10.2, 8.3, 6.4 Hz, 1H, CH_2=CH), 5.20-5.10 (m, 2H, CH_2=CH), 2.74 (dd, J = 13.7, 6.4 Hz, 1H, CHHCH=CH_2), 2.55 (dd, J = 13.7, 8.3 Hz, 1H, CHHCH=CH_2), 2.09 (br s, 1H, OH), 1.59 (s, 3H, CCH_3); ^{13}C NMR (101 MHz, $CDCl_3$): δ = 146.9, 140.9, 139.6, 133.8, 128.9 (2C), 127.3, 127.2 (2C), 127.0 (2C), 125.4 (2C), 119.8, 73.7, 48.6, 30.1; IR (ATR): ν = 3443, 2976, 1486, 1006, 998, 915cm^{-1}; MS (EI) m/z (%): 220 (M^+-H_2O, 100), 219 (27), 206 (15), 205 (84), 203 (24), 202 (20), 198 (10), 197 (63), 196 (17), 181 (38), 179 (43), 178 (49), 165 (14), 153 (18), 152 (39), 151 (13), 128 (10).

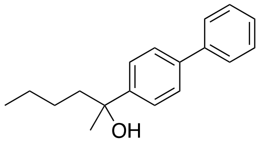

2-([1,1'-biphenyl]-4-yl)hexan-2-ol (11e): Colorless oil; R_f = 0.37 (hexane/ethyl acetate: 4/1); t_r = 16.97; ^{1}H NMR (400 MHz, $CDCl_3$): δ = 7.65-7.55 (m, 4H, ArH), 7.55-7.50 (m, 2H, ArH), 7.50-7.40 (m, 2H, ArH), 7.35-7.30 (m, 1H, ArH), 1.90-1.75 (m, 2H, HOCCH_2), 1.70 (br s, 1H, OH), 1.60 (s, 3H, CH_3COH), 1.35-1.15 (m, 4H, CH_3CH_2CH_2), 0.87 (t, J = 5.8 Hz, 3H, C$H_3$$CH_2$$CH_2$); ^{13}C NMR (101 MHz, $CDCl_3$): δ = 147.3, 141.0, 139.5, 128.9 (2C), 127.3, 127.2 (2C), 127.0 (2C), 125.4 (2C), 74.8, 44.1, 30.3, 26.3, 23.2, 14.2; IR (ATR): ν = 3404, 3028, 2931, 1486, 1006 cm^{-1}; MS (EI) m/z (%): 254 (M^+, 1), 237 (13), 236 (66), 221 (10), 207 (80), 197 (33),

195 (25), 194 (100), 192 (16), 191 (15), 179 (52), 178 (38), 165 (47); HRMS calcd. for $C_{18}H_{22}O$: 254.1671; found: 254.1678.

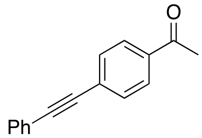

1-(4-(phenylethynyl)phenyl)ethanone (13a):(Gholap et al., 2005) White solid; R_f = 0.27 (hexane/ethyl acetate: 19/1); m.p. 95-97 °C; t_r = 15.7; ^{1}H NMR (300 MHz, $CDCl_3$): δ = 8.00-7.90 (m, 2H, ArH), 7.65-7.60 (m, 2H, ArH), 7.60-7.50 (m, 2H, ArH), 7.40-7.35 (m, 3H, ArH), 2.62 (s, 3H, $COCH_3$); ^{13}C NMR (75 MHz, $CDCl_3$): δ = 197.4, 136.3, 131.8 (4C), 128.9, 128.6 (2C), 128.4 (2C), 128.3, 122.8, 92.8, 88.7, 26.8; IR (ATR): ν = 1677, 1592, 1176, 833 cm^{-1}; MS (EI) *m/z* (%): 220 (M^+, 70), 207 (15), 206 (16), 205 (100), 177 (19), 176 (43), 151 (11).

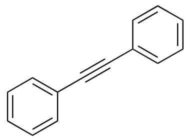

1,2-diphenylethyne (13b): (Bong Park and Alper, 2004) White solid; R_f = 0.5 (Hexane); m.p. 59-61 °C; t_R= 12.43 min; ^{1}H NMR (300 MHz, $CDCl_3$): *δ*= 7.55-7.50 (m, 4H, ArH), 7.40-7.30 (m, 6H, ArH) ppm; ^{13}C NMR (100 MHz, $CDCl_3$): *δ*= 131.8, 128.5, 128.4, 123.4, 89.5 ppm; IR (ATR): *ν* = 3063, 2921, 2851, 1950, 1882, 1806, 1758, 1674, 1599, 1492, 1442, 1312, 1280, 1156, 11069, 1025, 916 cm^{-1}; MS (EI, 70 eV): *m/z* (%): 179 (M^+ +1, 15%) 178 (M^+, 100%), 177 (10), 176 (22).

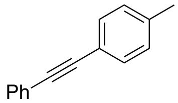

1-methyl-4-(phenylethynyl)benzene (13c):(Gholap et al., 2005) White solid; R_f = 0.4 (hexane); m.p. 73-75 °C; t_r = 13.6; ^{1}H NMR (300 MHz, $CDCl_3$): δ = 7.55-7.50 (m, 2H, ArH), 7.43 (dt, *J* = 7.9, 1.7 Hz, 2H, ArH), 7.35-7.30 (m, 3H, ArH), 7.15 (d, *J* = 7.9 Hz), 2.36 (s, 3H, CH_3); ^{13}C NMR (75 MHz, $CDCl_3$): δ = 138.5, 131.7, 131.6, 129.3, 128.5, 128.2, 123.6, 120.3, 89.7, 88.9; IR (ATR): ν = 3029, 2918, 1508, 1440, 753 cm^{-1}; MS (EI) *m/z* (%): 193 (M^++1, 16), 192 (M^+, 100), 191 (M^+- 1, 48), 190 (12), 189 (23), 165 (12).

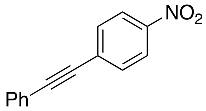

1-nitro-4-(phenylethynyl)benzene (13d):(Gholap et al., 2005) Yellow solid; R_f = 0.3 (hexane:ethyl acetate: 19/1); m.p. 111-112 °C; t_r = 15.8; ^{1}H NMR (300 MHz, $CDCl_3$): δ = 8.21 (d, *J* = 8.8 Hz, 2H, ArH), 7.66 (d, *J* = 8.8 Hz, 2H, ArH), 7.56 (dd, *J* = 6.6, 3.0 Hz, 2H, ArH), 7.45-7.30 (m, 3H, ArH); ^{13}C NMR (75 MHz, $CDCl_3$): δ = 147.1, 132.4 (2C), 132.0 (2C), 130.4, 129.4, 128.7 (2C), 123.7 (2C), 122.2, 94.8, 87.7; IR (ATR): ν = 1509, 1345, 686, 615 cm^{-1}; MS (EI) *m/z* (%): 224 (M^++1, 16), 223 (M^+, 100), 193 (26), 177 (11), 176 (46), 165 (16), 151 (17).

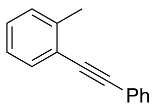

1-methyl-2-(phenylethynyl)benzene (13e):(Kakusawa et al., 2005) Colourless oil; R_f = 0.4 (hexane); t_r = 13.3; ^{1}H NMR (300 MHz, $CDCl_3$): δ = 7.55-7.45 (m, 3H, ArH), 7.40-7.30 (m, 3H, ArH), 7.25-7.20 (m, 2H, ArH), 7.20-7.10 (m, 1H, ArH), 2.52 (s, 3H, CH_3); ^{13}C NMR (75 MHz, $CDCl_3$): δ = 140.3, 131.9, 131.6 (2C), 129.6, 128.5 (2C), 128.4, 128.3, 125.7, 123.7, 123.1, 93.5, 88.5, 20.9; IR (ATR): ν = 3059, 1492, 751, 687 cm^{-1}; MS (EI) *m/z* (%): 193 (M^++1, 14), 192 (M^+, 100), 191 (M^+- 1, 94), 190 (12), 189 (31), 165 (19).

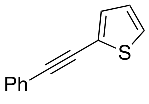

2-(phenylethynyl)thiophene (13f):(Kim and Lee, 2009) White solid; R_f = 0.6 (hexane/ethyl acetate: 19/1); m.p. 50-51 °C; t_r = 12.9; ^{1}H NMR (300 MHz, $CDCl_3$): δ = 7.55-7.45 (m, 2H, ArH), 7.40-7.30 (m, 3H, ArH), 7.30-7.25 (m, 2H, ArH), 7.05-6.95 (m, 1H, ArH); ^{13}C NMR (75 MHz, $CDCl_3$): δ = 132.0, 131.5 (2C), 128.6, 128.5 (2C), 127.4, 127.2, 123.4, 123.0, 93.2, 82.7; IR (ATR): ν = 1485, 851, 753, 687 cm^{-1}; MS (EI): *m/z* (%):185 (M^++1, 15), 184 (M^+, 100), 152 (12), 139 (14).

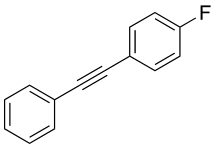

1-fluoro-4-(phenylethynyl)benzene (13g):(Döbele et al., 2010) White solid; R_f = 0.6 (Hexane); m.p. 103-106 °C; t_R= 13.55 min; ^{1}H NMR (300 MHz, $CDCl_3$): δ= 7.55-7.45 (m, 4H, ArH), 7.40-7.30 (m, 3H, ArH), 7.10-7.00 (m, 2H, ArH) ppm; ^{13}C NMR (100 MHz, $CDCl_3$): δ= 162.6 (d, J = 249.6 Hz), 161.4, 133.6 (d, J = 8.4 Hz), 131.7, 128.5 (2C), 123.4, 119.5 (d, J = 3.4 Hz), 115.8 (d, J = 22.1 Hz), 89.2, 88.4 ppm; IR (ATR): ν = 2926, 2858, 1590, 1214, 1158, 835, 756, 686 cm^{-1}; MS (EI, 70 eV): *m/z* 197 (M^++1, 15%), 196 (M^+, 100%), 194 (14), 175 (10).

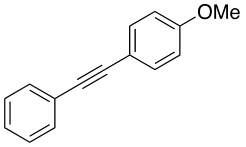

1-methoxy-4-(phenylethynyl)benzene (13h):(Bong Park and Alper, 2004) White solid; R_f = 0.47 (Hexane/AcOEt 98/2); m.p. 61-62.5 °C; t_R= 17.64 min; ^{1}H NMR (300 MHz, $CDCl_3$): δ= 7.55-7.45 (m, 4H, ArH), 7.35-7.30 (m, 3H, ArH), 6.85-6.80 (m, 2H, ArH), 3.85 (s, 3H, OCH_3) ppm; ^{13}C NMR (100 MHz, $CDCl_3$): δ= 159.7, 133.2, 131.6, 128.4, 128.0, 123.7, 115.5, 114.1, 89.5, 88.2, 55.4 ppm; IR (ATR): ν = 2919, 2852, 2213, 1603, 1594, 1506, 1244, 1024, 835, 753, 688 cm^{-1}; MS (EI, 70 eV): *m/z* 209 (M^++1, 16%), 208 (M^+, 100%), 193 (42), 165 (30), 164 (11).

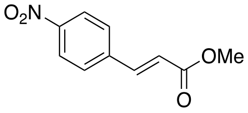

(*E*)-methyl 3-(4-nitrophenyl)acrylate (15a):(Evangelisti et al., 2009) Off-white solid; R_f = 0.33 (hexane/ethyl acetate: 4/1); m.p. 131-133 °C; t_r = 13.9; ^{1}H NMR (300 MHz, $CDCl_3$): δ = 8.30-8.15 (m, 2H, ArH), 7.73 (d, J = 16.1 Hz, 1H, *H*C=CH), 7.70-7.65 (m, 2H, ArH), 6.57 (d, J = 16.1 Hz, 1H, *H*C=CH), 3.84 (s, 3H, CO_2CH_3); ^{13}C NMR (75 MHz, $CDCl_3$): δ = 166.5, 148.6, 142.0, 140.6, 128.7 (2C), 124.3 (2C), 122.2, 52.2; IR (ATR): ν = 1718, 1509, 1333, 846 cm^{-1}; MS (EI) *m*/*z* (%): 207 (M^+, 51), 176 (100), 146 (17), 130 (14), 129 (14), 118 (13), 102 (29), 90 (14), 51 (11).

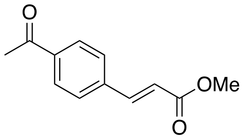

Methyl (*E*)-3-(4-acetylphenyl)acrylate (15b):(Bernini et al., 2008) White solid, R_f= 0.25 (Hexane/AcOEt 4/1); m.p. 104.4-104.7 °C; t_r =15.21 min; ^{1}H NMR (300 MHz, $CDCl_3$): δ= 8.00-7.95 (m, 2H, ArH), 7.71 (d, J = 16.1 Hz, 1H, *H*C=CH), 7.65-7.60 (m, 2H, ArH), 6.53 (d, J = 16.1 Hz, 1H, HC=C*H*), 3.83 (s, 3H, OCH_3), 2.62 (s, 3H, $COCH_3$) ppm; ^{13}C NMR (75 MHz, $CDCl_3$): δ= 197.4, 167.1, 143.4, 138.8, 138.2, 129.0, 128.3, 120.5, 52.1, 26.8 ppm; IR (ATR): ν = 2959, 2922, 2852, 1707, 1680, 1638, 1429, 1325, 1314, 1205, 1172, 988, 824 cm^{-1}; MS (70 eV, EI): *m*/*z* (%):204 (M^+, 31%), 190 (12), 189 (100), 102 (10).

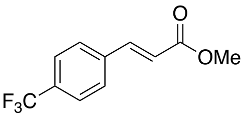

Methyl (*E*)-3-(4-(trifluoromethyl)phenyl)acrylate (15c):(O'Brien et al., 2009) White solid, R_f= 0.50 (Hexane/AcOEt 4/1); m.p. 73.1-73.4 °C; t_r =11.43 min; ^{1}H NMR (300 MHz, $CDCl_3$): δ= 7.71 (d, J = 16.0 Hz, 1H, *H*C=CH), 7.65-7.60 (m, 4H), 6.52 (d, J = 16.0 Hz, 1H, HC=C*H*), 3.83 (s, 3H, OCH_3) ppm; ^{13}C NMR (75 MHz, $CDCl_3$): δ= 170.0, 143.1, 137.9, 132.4-131.4 (q, J = 32.4 Hz), 128.3, 126.1-125.3 (q, J = 3.8 Hz), 128.0-119.9 (q, J = 271.8 Hz), 120.5, 52.1 ppm; IR (ATR): ν = 2957, 2923, 2850, 1708, 1639, 1316, 1159, 1107, 1064, 834 cm^{-1}; MS (70 eV, EI): *m*/*z* (%):230 (M^+, 39%), 229 (15), 220 (11), 199 (100), 171 (34), 151 (38).

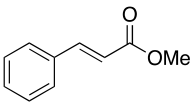

Methyl cinnamate (15d):(Yang et al., 2015) White solid, R_f= 0.50 (Hexane/AcOEt 4/1); m.p. 33-35 °C; t_r =11.54 min; ^{1}H NMR (300 MHz, $CDCl_3$): δ= 7.70 (d, J = 16.0 Hz, 1H, *H*C=CH), 7.55-7.50 (m, 2H, ArH), 7.40-7.35 (m, 3H, ArH), 6.45 (d, J = 16.0 Hz, 1H, HC=C*H*), 3.81 (s, 3H, OCH_3) ppm; ^{13}C NMR (75 MHz, $CDCl_3$): δ= 167.6, 145.0, 134.5, 130.4, 129.0, 128.2, 117.9, 51.8 ppm; IR (ATR): ν = 2944, 1711, 1636, 771 cm^{-1}; MS (70 eV, EI): *m*/*z* (%): 162 (M^+, 55%), 161 (M^+-1, 29), 131 (100), 103 (57), 102 (15), 77 (30), 51 (15).

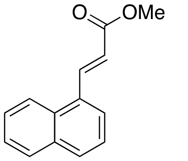

(*E*)-methyl 3-(naphthalen-1-yl)acrylate (15e):(Yang et al., 2015) Yellow oil; R_f = 0.47 (hexane/ethyl acetate: 4/1) ; t_r = 14.6; ^{1}H NMR (300 MHz, $CDCl_3$): δ = 8.55 (d, *J* = 15.8 Hz, 1H, *H*C=CH), 8.2 (d, *J* = 8.2 Hz, 1H, ArH), 7.95-7.85 (m, 2H, ArH), 7.75 (d, *J* = 7.2 Hz, 1H, ArH), 7.65-7.40 (m, 3H, ArH), 6.54 (d, *J* = 15.8 Hz, 1H, *H*C=CH), 3.87 (s, 3H, CO_2CH_3); ^{13}C NMR (75 MHz, $CDCl_3$): δ = 167.4, 141.9, 133.7, 131.8, 131.5, 130.6, 128.8, 126.9, 126.3, 125.5, 125.1, 123.4, 120.5, 51.9; IR (ATR): *ν* = 2947, 1709, 1630, 1165, 799 cm^{-1}; MS (EI) *m*/*z* (%): 212 (M$^+$, 29), 181 (14), 154 (13), 153 (100), 152 (76), 151 (17), 76 (13).

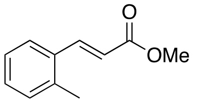

(*E*)-methyl 3-(*o*-tolyl)acrylate (15f):(Yang et al., 2015) Yellow oil; R_f = 0.57 (hexane/ethyl acetate: 4/1); t_r = 11.2; ^{1}H NMR (300 MHz, $CDCl_3$): δ = 7.99 (d, *J* = 15.9 Hz, 1H, *H*C=CH), 7.70-7.45 (m, 1H, ArH), 7.40-7.10 (m, 3H, ArH), 6.36 (d, *J* = 15.9 Hz, 1H, *H*C=CH), 3.81 (s, 3H, CO_2CH_3), 2.44 (s, 3H, Ar-CH_3); ^{13}C NMR (75 MHz, $CDCl_3$): δ = 166.5, 148.6, 142.0, 140.6, 128.7 (2C), 124.3 (2C), 122.2, 52.2; IR (ATR): *ν* 2948, 1714, 1433, 1168, 761 cm^{-1}; MS (EI) *m*/*z* (%): 176 (M$^+$, 34), 161 (29), 146 (13), 145 (100), 144 (29), 117 (46), 116 (86), 115 (95), 91 (26), 65 (10).

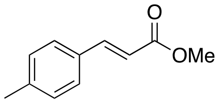

(*E*)-methyl 3-(*p*-tolyl)acrylate (15g):(Yang et al., 2015) White solid; R_f = 0.57 (hexane/ethyl acetate: 4/1); m.p. 54-55 °C; t_r =11.5; ^{1}H NMR (300 MHz, $CDCl_3$): δ = 7.67 (d, *J* = 16.0 Hz, 1H, *H*C=CH), 7.41 (d, *J* = 8.1 Hz, 2H, ArH), 7.18 (d, *J* = 8.0 Hz, 2H, ArH), 6.39 (d, *J* = 16.0 Hz, 1H, *H*C=CH), 3.79 (s, 3H, CO_2CH_3), 2.36 (s, 3H, Ar-CH_3); ^{13}C NMR (75 MHz, $CDCl_3$): δ = 167.5, 144.8, 140.6, 131.6, 129.6 (2C), 128.0 (2C), 116.6, 51.5, 21.4; IR (ATR): ν 2945, 1703, 1604, 815 cm^{-1}; MS (EI) *m*/*z* (%): 176 (M$^+$, 65), 175 (M$^+$-1, 15), 146 (11), 145 (100), 117 (28), 116 (16), 115 (51), 91 (18).

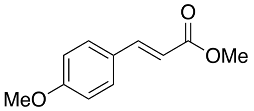

(*E*)-methyl 3-(4-methoxyphenyl)acrylate (15h):(Yang et al., 2015) White solid; R_f = 0.47 (hexane/ethyl acetate: 4/1); m.p. 83-84 °C; t_r = 12.9; ^{1}H NMR (300 MHz, $CDCl_3$): δ = 7.65 (d, *J* = 16.0 Hz, 1H, *H*C=CH), 7.55-7.40 (m, 2H, ArH), 7.00-6.80 (m, 2H, ArH), 6.31 (d, *J* = 16.0 Hz, 1H, *H*C=CH), 3.84 (s, 3H, CO_2CH_3), 3.79 (s, 3H, CO_2CH_3); ^{13}C NMR (75 MHz, $CDCl_3$): δ = 167.6, 161.3, 144.4, 129.7 (2C), 127.0, 115.1, 114.2 (2C), 55.2, 51.5; IR (ATR): ν = 2038, 1711, 1600, 819 cm^{-1}; MS (EI) *m*/*z* (%): 192 (M$^+$, 74), 162 (11), 161 (100), 134 (13), 133 (28), 118 (12), 90 (11), 89 (16).

4 References

Bai, L., and Wang, J.-X. (2008). Reusable, polymer-supported, palladium-catalyzed, atom-efficient coupling reaction of aryl halides with sodium tetraphenylborate in water by focused microwave irradiation. *Adv. Synth. Catal.* 350(2)**,** 315-320. doi: 10.1002/adsc.200700361.

Bandari, R., Hoeche, T., Prager, A., Dirnberger, K., and Buchmeiser, M.R. (2010). Ring-Opening Metathesis Polymerization Based Pore-Size-Selective Functionalization of Glycidyl Methacrylate Based Monolithic Media: Access to Size-Stable Nanoparticles for Ligand-Free Metal Catalysis. *Chem. - Eur. J.* 16(15)**,** 4650-4658. doi: 10.1002/chem.200902654.

Bernini, R., Cacchi, S., Fabrizi, G., Forte, G., Niembro, S., Petrucci, F., et al. (2008). Phosphine-Free Perfluoro-Tagged Palladium Nanoparticles Supported on Fluorous Silica Gel: Application to the Heck Reaction. *Organic Letters* 10(4)**,** 561-564. doi: 10.1021/ol7024845.

Bong Park, S., and Alper, H. (2004). Recyclable Sonogashira coupling reactions in an ionic liquid, effected in the absence of both a copper salt and a phosphine. *Chemical Communications* (11)**,** 1306-1307. doi: 10.1039/B402477J.

Brekan, J.A., Chernyak, D., White, K.L., and Scheidt, K.A. (2012). Lewis base-promoted carbon-carbon sp3-sp3 coupling reactions of α-silyl silylethers. *Chem. Sci.* 3(4)**,** 1205-1210. doi: 10.1039/c2sc00581f.

Bunda, S., Udvardy, A., Voronova, K., and Joo, F. (2018). Organic Solvent-Free, Pd(II)-Salan Complex-Catalyzed Synthesis of Biaryls via Suzuki-Miyaura Cross-Coupling in Water and Air. *J. Org. Chem.* 83(24)**,** 15486-15492. doi: 10.1021/acs.joc.8b02340.

Döbele, M., Vanderheiden, S., Jung, N., and Bräse, S. (2010). Synthesis of Aryl Fluorides on a Solid Support and in Solution by Utilizing a Fluorinated Solvent. *Angewandte Chemie International Edition* 49(34)**,** 5986-5988. doi: doi:10.1002/anie.201001507.

Evangelisti, C., Panziera, N., Pertici, P., Vitulli, G., Salvadori, P., Battocchio, C., et al. (2009). Palladium nanoparticles supported on polyvinylpyridine: Catalytic activity in Heck-type reactions and XPS structural studies. *J. Catal.* 262(2)**,** 287-293. doi: 10.1016/j.jcat.2009.01.005.

Fairlamb, I.J.S., Kapdi, A.R., and Lee, A.F. (2004). η2-dba Complexes of Pd(0): The substituent effect in Suzuki-Miyaura coupling. *Org. Lett.* 6(24)**,** 4435-4438. doi: 10.1021/ol048413i.

Furuyama, T., Yonehara, M., Arimoto, S., Kobayashi, M., Matsumoto, Y., and Uchiyama, M. (2008). Development of highly chemoselective bulky zincate complex, tBu4ZnLi2: design, structure, and practical applications in small-/macromolecular synthesis. *Chem. - Eur. J.* 14(33)**,** 10348-10356. doi: 10.1002/chem.200800536.

Gholap, A.R., Venkatesan, K., Pasricha, R., Daniel, T., Lahoti, R.J., and Srinivasan, K.V. (2005). Copper- and Ligand-Free Sonogashira Reaction Catalyzed by Pd(0) Nanoparticles at Ambient Conditions under Ultrasound Irradiation. *J. Org. Chem.* 70(12)**,** 4869-4872. doi: 10.1021/jo0503815.

Ilie, A., Roiban, G.-D., and Reetz, M.T. (2017). Di-tert-butyl N,N-diethylphosphoramidite as an Air Stable Ligand for Suzuki-Miyaura and Buchwald-Hartwig Reactions. *ChemistrySelect* 2(4)**,** 1392-1397. doi: 10.1002/slct.201700086.

Iranpoor, N., Rahimi, S., and Panahi, F. (2016). In situ generated and stabilized Pd nanoparticles by N2,N4,N6-tridodecyl-1,3,5-triazine-2,4,6-triamine (TDTAT) as a reactive and efficient catalyst for the Suzuki-Miyaura reaction in water. *RSC Adv.* 6(4)**,** 3084-3090. doi: 10.1039/C5RA24120K.

Kakusawa, N., Yamaguchi, K., and Kurita, J. (2005). Palladium-catalyzed cross-coupling reaction of ethynylstibines with organic halides. *J. Organomet. Chem.* 690(12)**,** 2956-2966. doi: 10.1016/j.jorganchem.2005.03.021.

Karimi, B., Tavakolian, M., Mansouri, F., and Vali, H. (2019). Nanopalladium on Magnetic Ionic Nanoparticle Network (MINN) as an Efficient and Recyclable Catalyst with High Ionic Density and Dispersibility. *ACS Sustainable Chem. Eng.* 7(4)**,** 3811-3823. doi: 10.1021/acssuschemeng.8b04566.

Kienle, M., and Knochel, P. (2010). i-PrI Acceleration of Negishi Cross-Coupling Reactions. *Org. Lett.* 12(12)**,** 2702-2705. doi: 10.1021/ol1007026.

Kim, H., and Lee, P.H. (2009). Palladium-Catalyzed Decarboxylative sp-sp2 Cross-Coupling Reactions of Aryl and Vinyl Halides and Triflates with α,β-Ynoic Acids using Silver Oxide. *Adv. Synth. Catal.* 351(17)**,** 2827-2832. doi: 10.1002/adsc.200900502.

Kumar Manian, R., Park, K., and Lee, S. (2010). Synthesis of Amido-N-imidazolium Salts and their Applications as Ligands in Suzuki–Miyaura Reactions: Coupling of Hetero- aromatic Halides and the Synthesis of Milrinone and Irbesartan. *Adv. Synth. Catal.* 352(18)**,** 3255-3266. doi: 10.1002/adsc.201000592.

Kylmala, T., Kuuloja, N., Xu, Y., Rissanen, K., and Franzen, R. (2008). Synthesis of chlorinated biphenyls by Suzuki cross-coupling using diamine or diimine-palladium complexes. *Eur. J. Org. Chem.* (23)**,** 4019-4024. doi: 10.1002/ejoc.200800119.

Li, Q., Zhang, L.-M., Bao, J.-J., Li, H.-X., Xie, J.-B., and Lang, J.-P. (2014). Suzuki–Miyaura reactions promoted by a PdCl2/sulfonate-tagged phenanthroline precatalyst in water. *Applied Organometallic Chemistry* 28(12)**,** 861-867. doi: doi:10.1002/aoc.3227.

Li, S., Wang, J.-X., Wen, X., and Ma, X. (2011). Mild and efficient Barbier allylation reaction mediated by magnesium powder under solvent-free conditions. *Tetrahedron* 67(5)**,** 849-855. doi: 10.1016/j.tet.2010.12.035.

Lois, S., Flores, J.-C., Lere-Porte, J.-P., Serein-Spirau, F., Moreau, J.J.E., Miqueu, K., et al. (2007). How to build fully π-conjugated architectures with thienylene and phenylene fragments. *Eur. J. Org. Chem.* (24)**,** 4019-4031. doi: 10.1002/ejoc.200601114.

Mathew, P., Neels, A., and Albrecht, M. (2008). 1,2,3-Triazolylidenes as versatile abnormal carbene ligands for late transition metals. *J. Am. Chem. Soc.* 130(41)**,** 13534-13535. doi: 10.1021/ja805781s.

Monguchi, Y., Fujita, Y., Hashimoto, S., Ina, M., Takahashi, T., Ito, R., et al. (2011). Palladium on carbon-catalyzed solvent-free and solid-phase hydrogenation and Suzuki–Miyaura reaction. *Tetrahedron* 67(45)**,** 8628-8634. doi: https://doi.org/10.1016/j.tet.2011.09.043.

O'Brien, C.J., Tellez, J.L., Nixon, Z.S., Kang, L.J., Carter, A.L., Kunkel, S.R., et al. (2009). Recycling the Waste: The Development of a Catalytic Wittig Reaction. *Angew. Chem. Int. Ed.* 48(37)**,** 6836-6839. doi: doi:10.1002/anie.200902525.

Sharghi, H., Khalifeh, R., and Doroodmand, M.M. (2009). Copper nanoparticles on charcoal for multicomponent catalytic synthesis of 1,2,3-triazole derivatives from benzyl halides or alkyl halides, terminal alkynes and sodium azide in water as a "green" solvent. *Adv. Synth. Catal.* 351(1+2)**,** 207-218. doi: 10.1002/adsc.200800612.

Stevens, P.D., Fan, J., Gardimalla, H.M.R., Yen, M., and Gao, Y. (2005). Superparamagnetic Nanoparticle-Supported Catalysis of Suzuki Cross-Coupling Reactions. *Org. Lett.* 7(11)**,** 2085-2088. doi: 10.1021/ol050218w.

Wang, C., Luo, Q., Sun, H., Guo, X., and Xi, Z. (2007). Lithio Siloles: Facile Synthesis and Applications. *J. Am. Chem. Soc.* 129(11)**,** 3094-3095. doi: 10.1021/ja070404s.

Yang, L., Zhang, X., Mao, P., Xiao, Y., Bian, H., Yuan, J., et al. (2015). NCN pincer palladium complexes based on 1,3-dipicolyl-3,4,5,6-tetrahydropyrimidin-2-ylidenes: synthesis, characterization and catalytic activities. *RSC Advances* 5(33)**,** 25723-25729. doi: 10.1039/C5RA01706H.

Zhang, Z., and Wang, Z. (2006). Diatomite-Supported Pd Nanoparticles: An Efficient Catalyst for Heck and Suzuki Reactions. *J. Org. Chem.* 71(19)**,** 7485-7487. doi: 10.1021/jo061179k.

5 Copies of 1H and 13C NMR

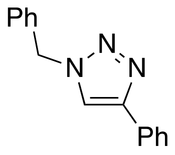

4

^{1}H NMR (300 MHz, $CDCl_3$)

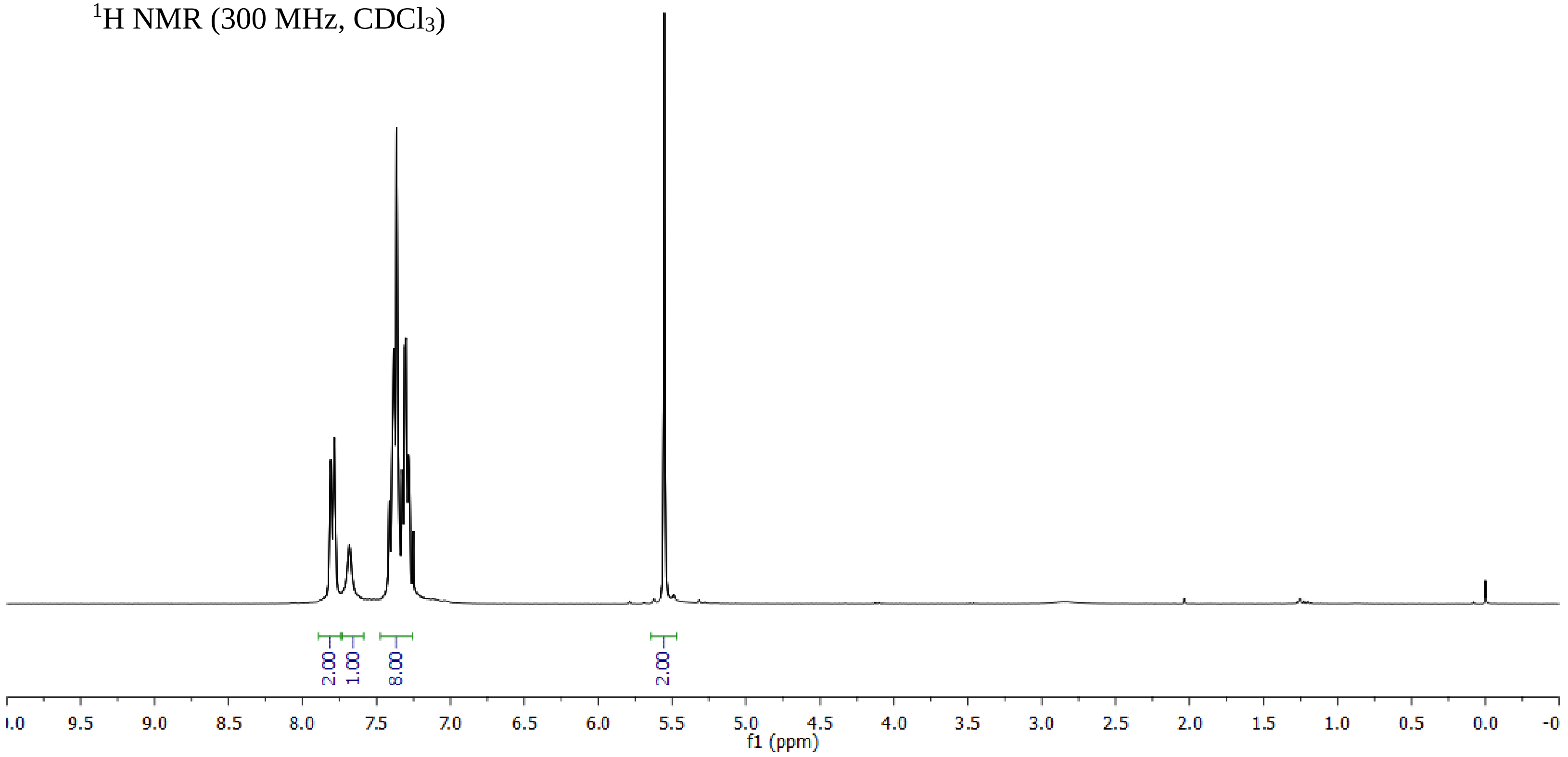

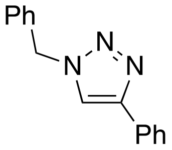

4

^{13}C NMR (101 MHz, $CDCl_3$)

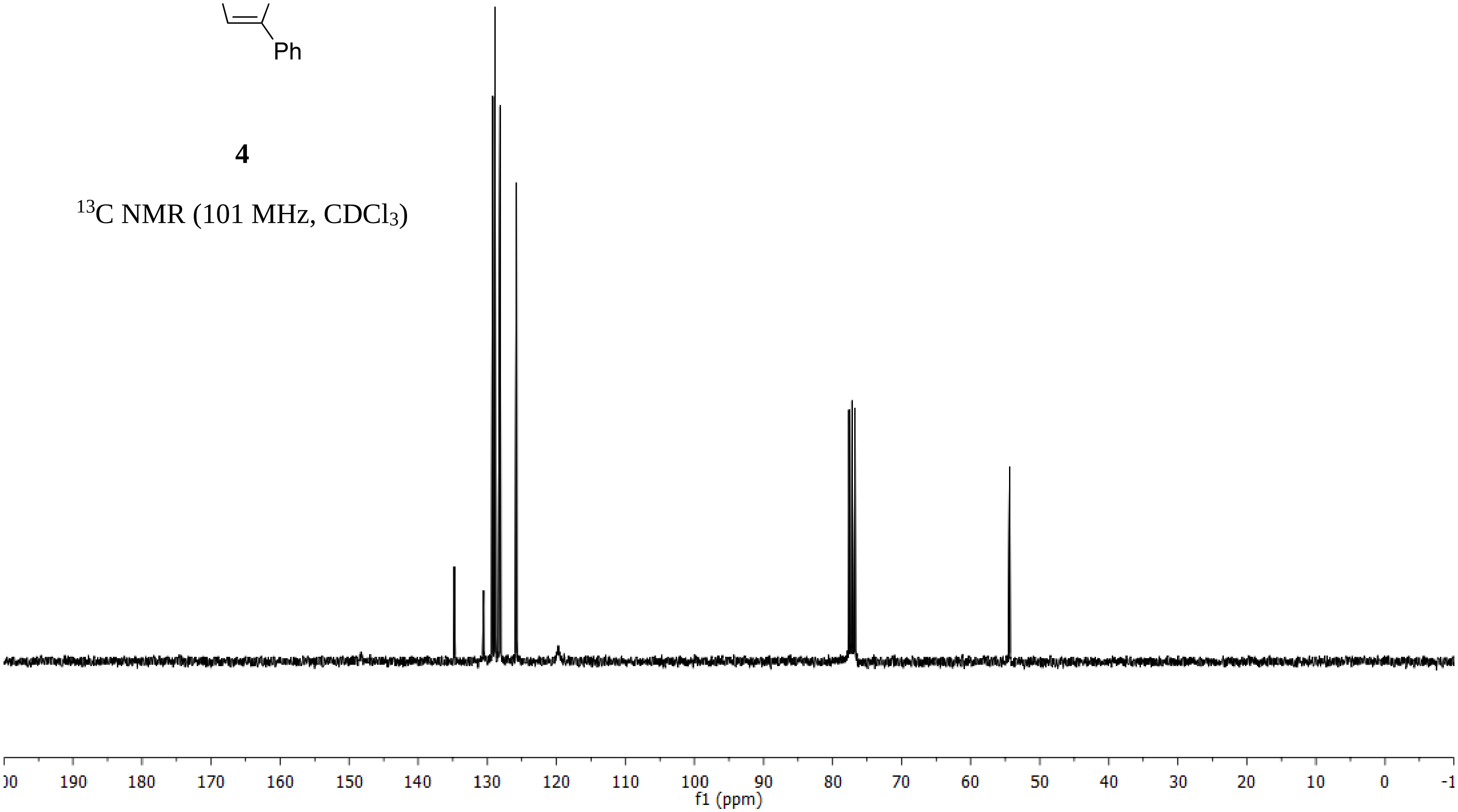

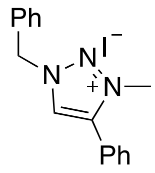

5

^{1}H NMR (300 MHz, $CDCl_3$)

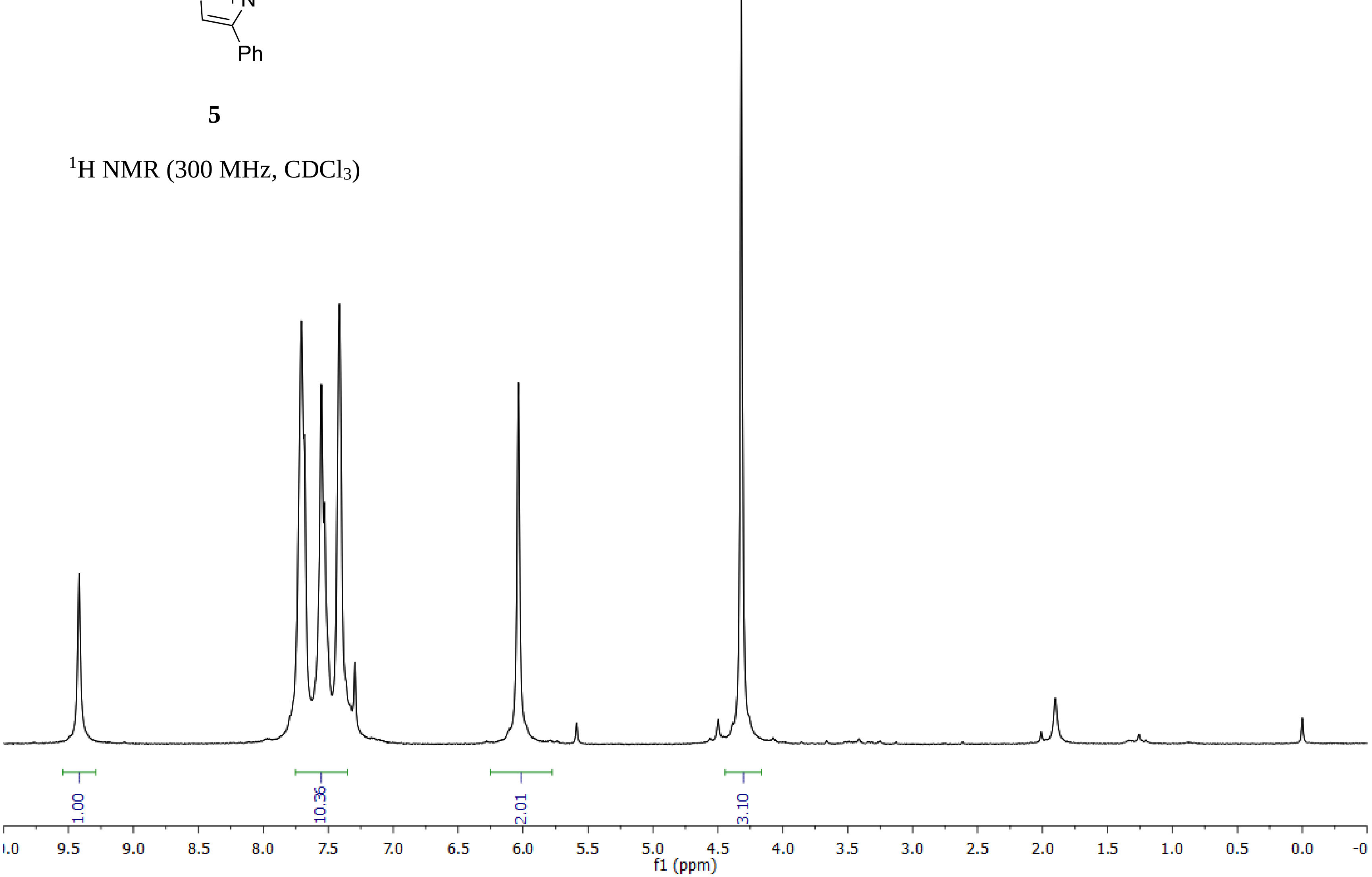

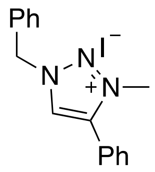

5

^{13}C NMR (101 MHz, $CDCl_3$)

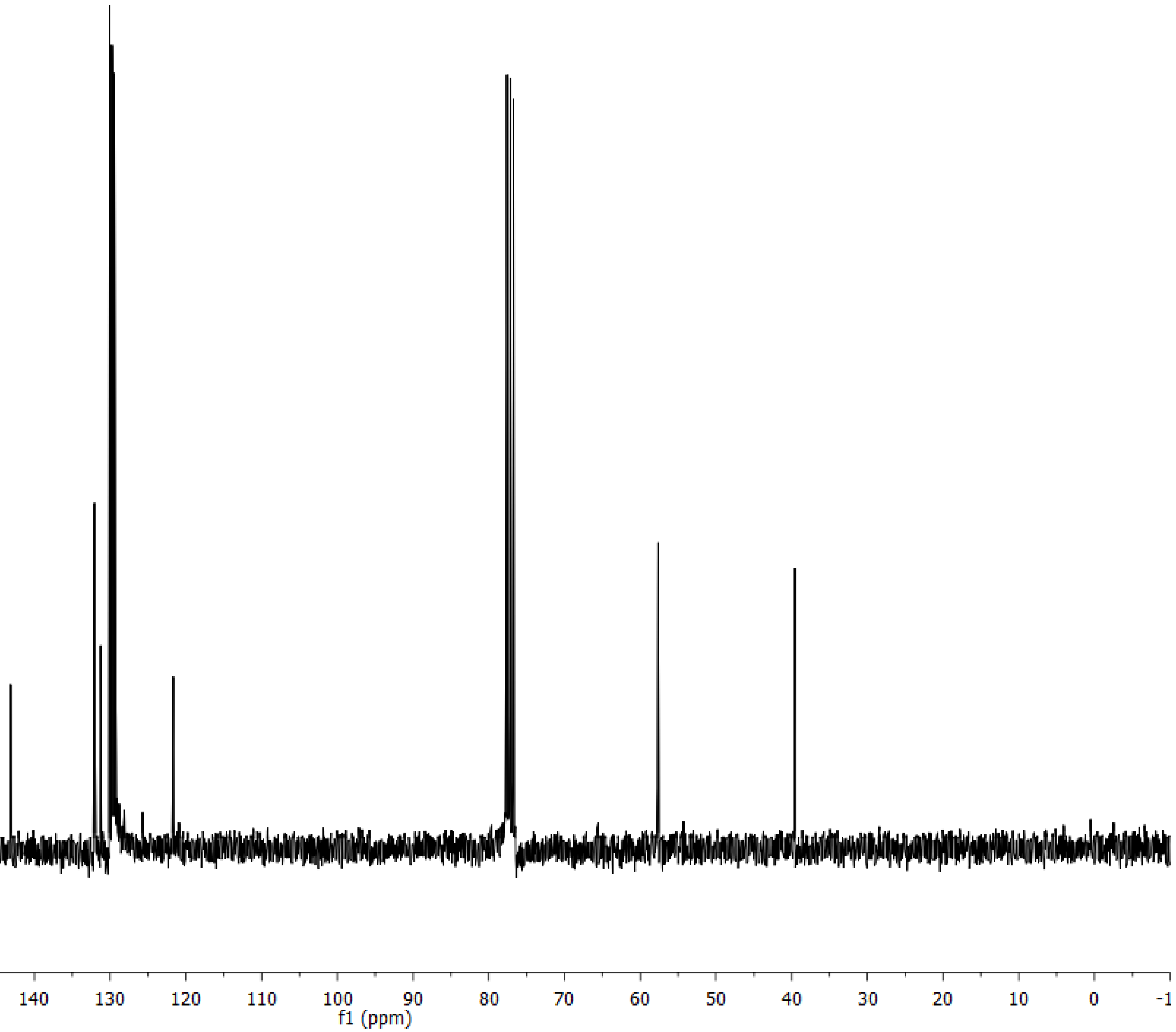

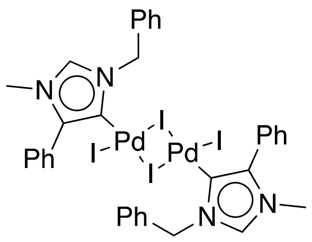

6

^{1}H NMR (300 MHz, DMSO-d$_6$)

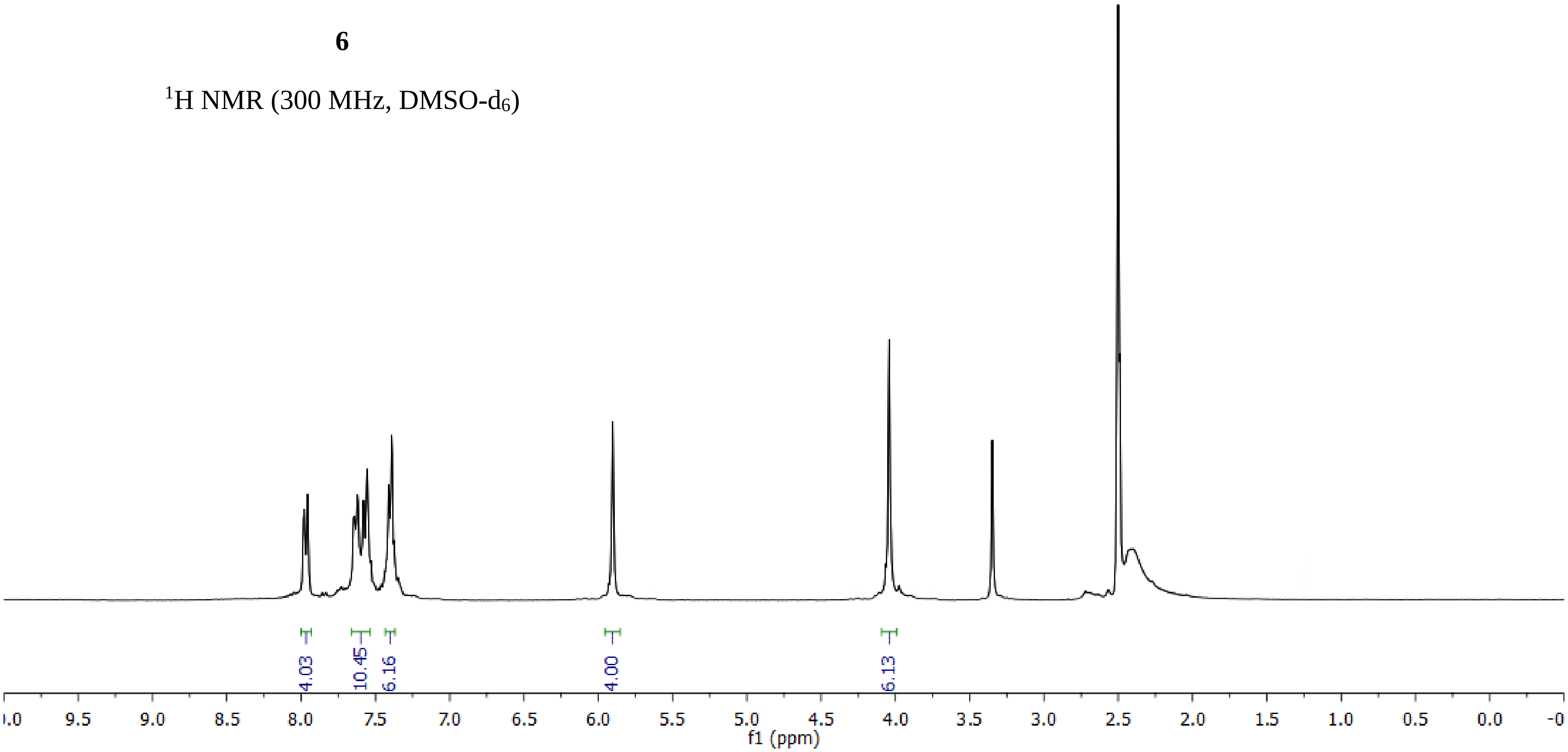

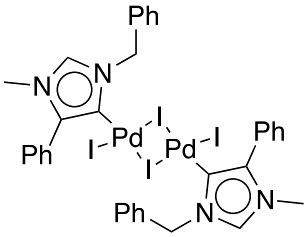

6

^{13}C NMR (101 MHz, DMSO-d_6)

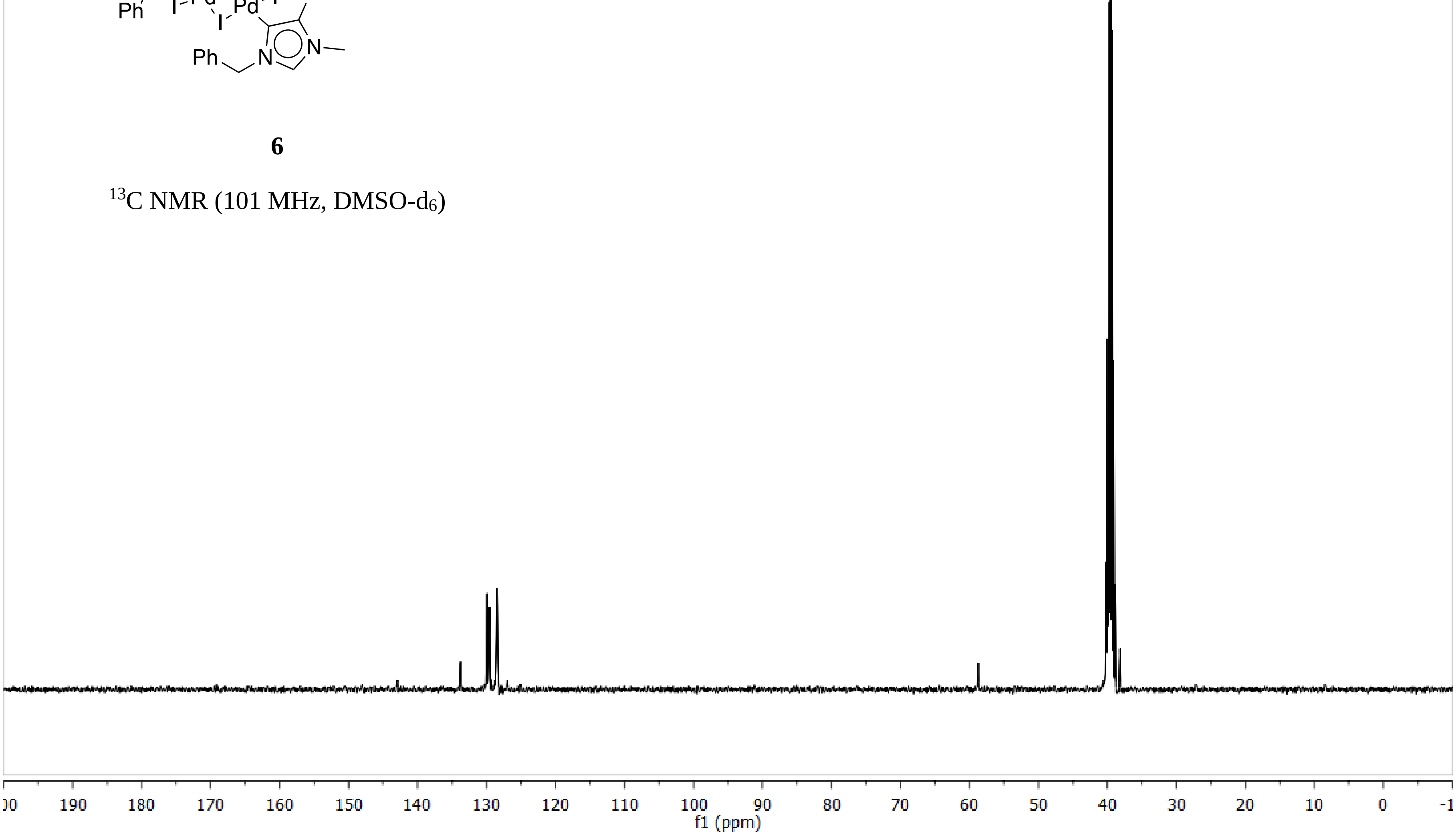

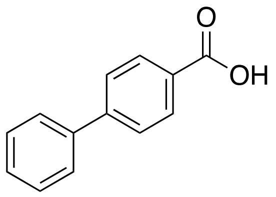

9a

^{1}H NMR (300 MHz, DMSO-d_6)

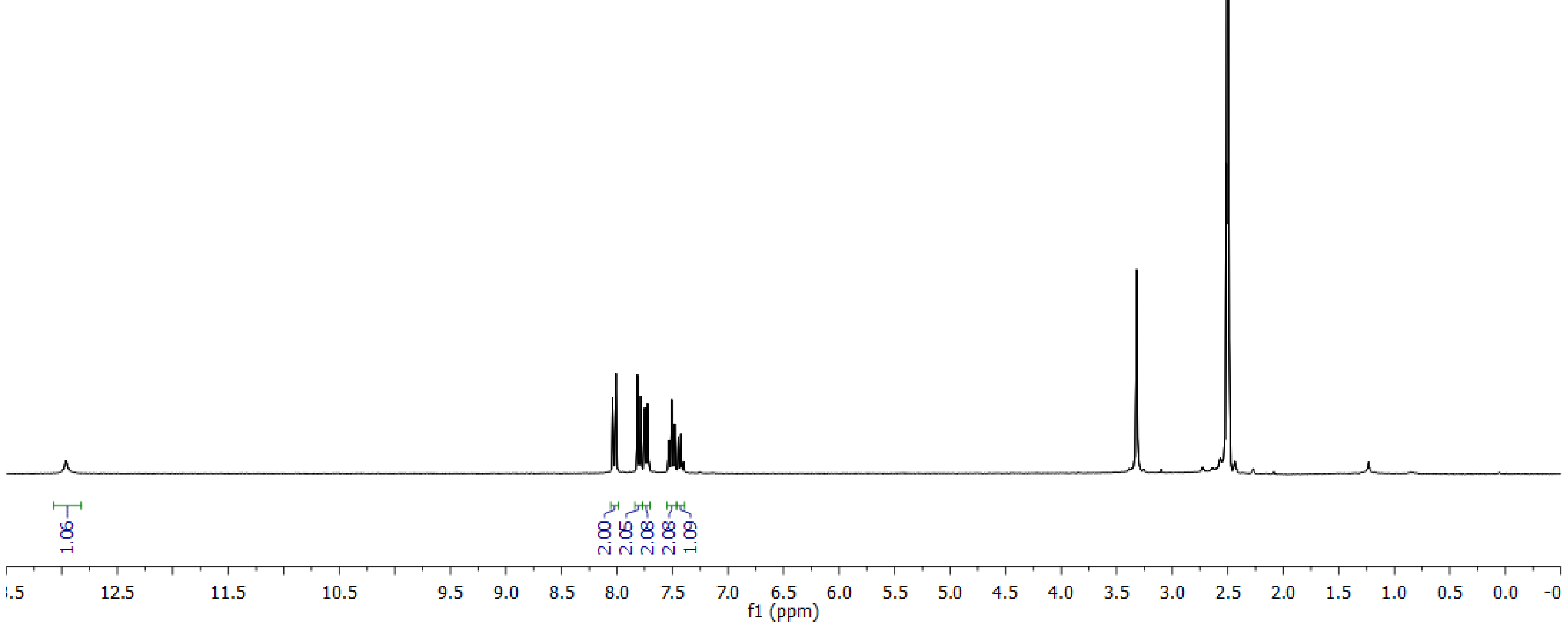

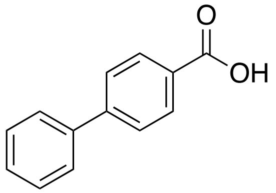

9a

^{13}C NMR (101 MHz, DMSO-d_6)

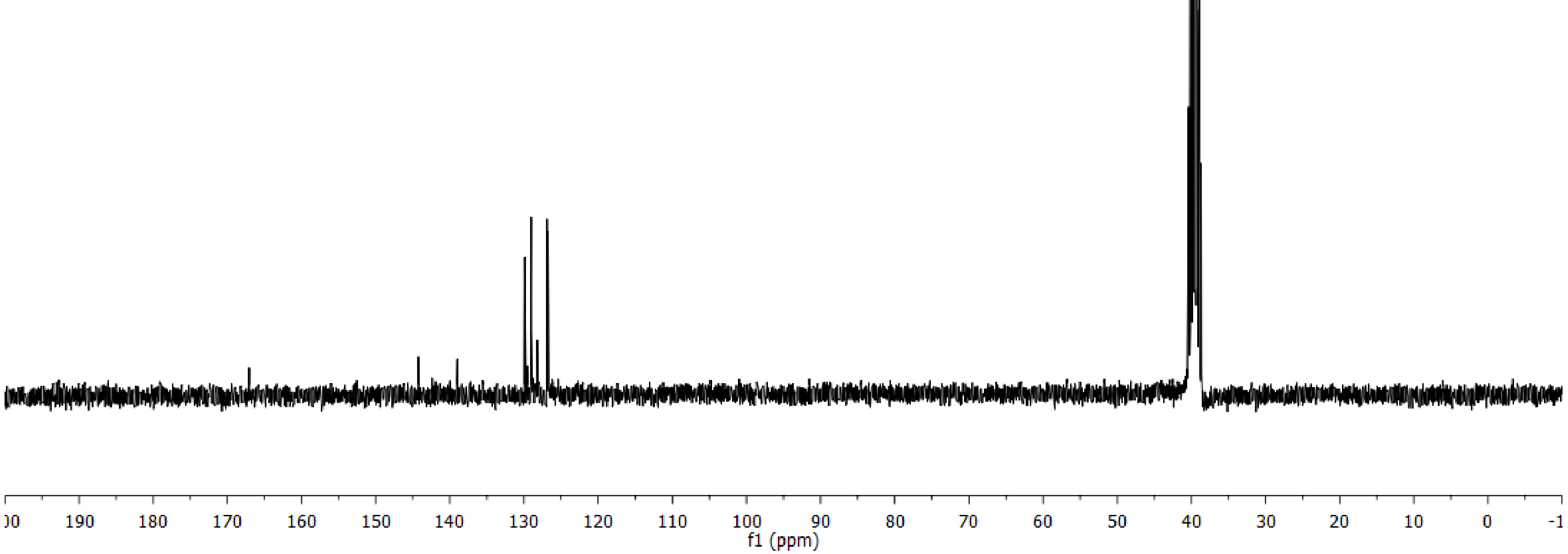

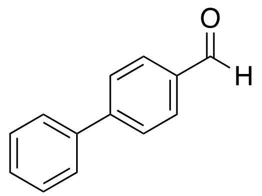

9b

^{1}H NMR (300 MHz, $CDCl_3$)

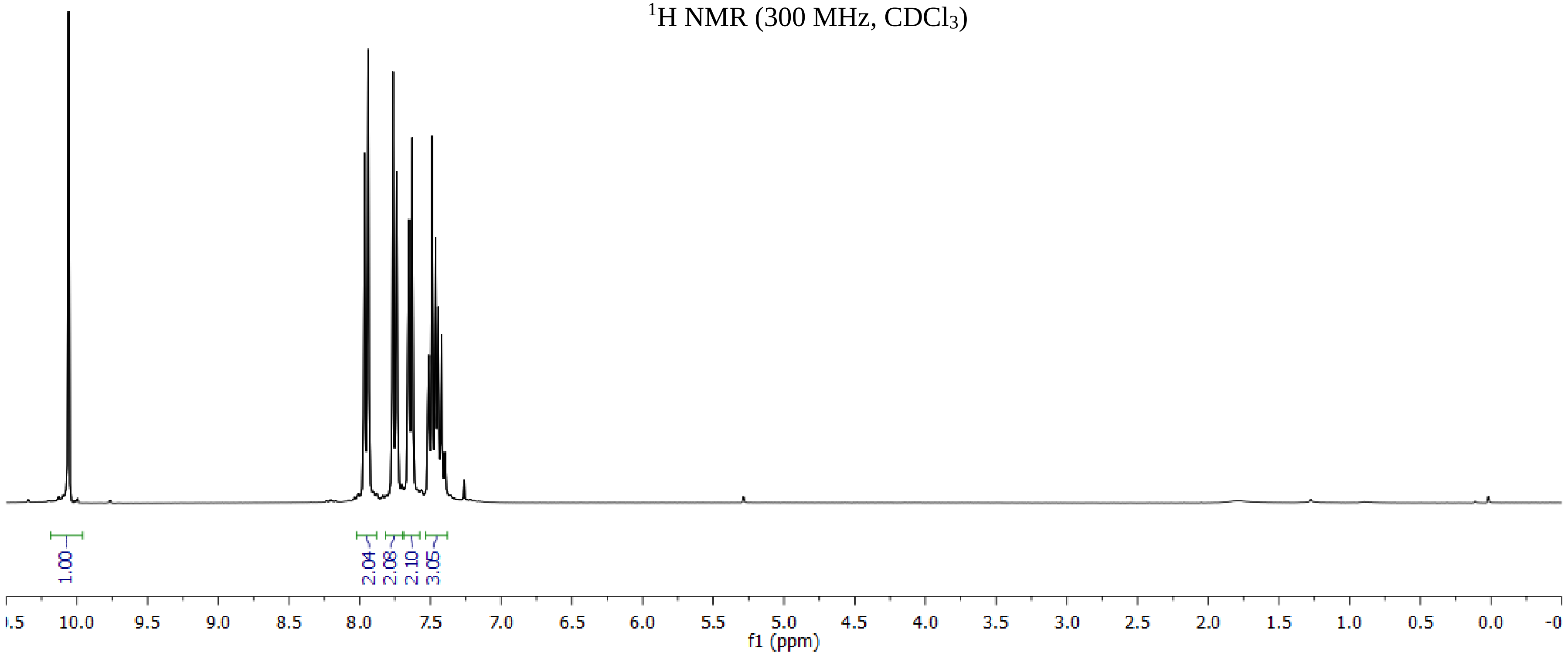

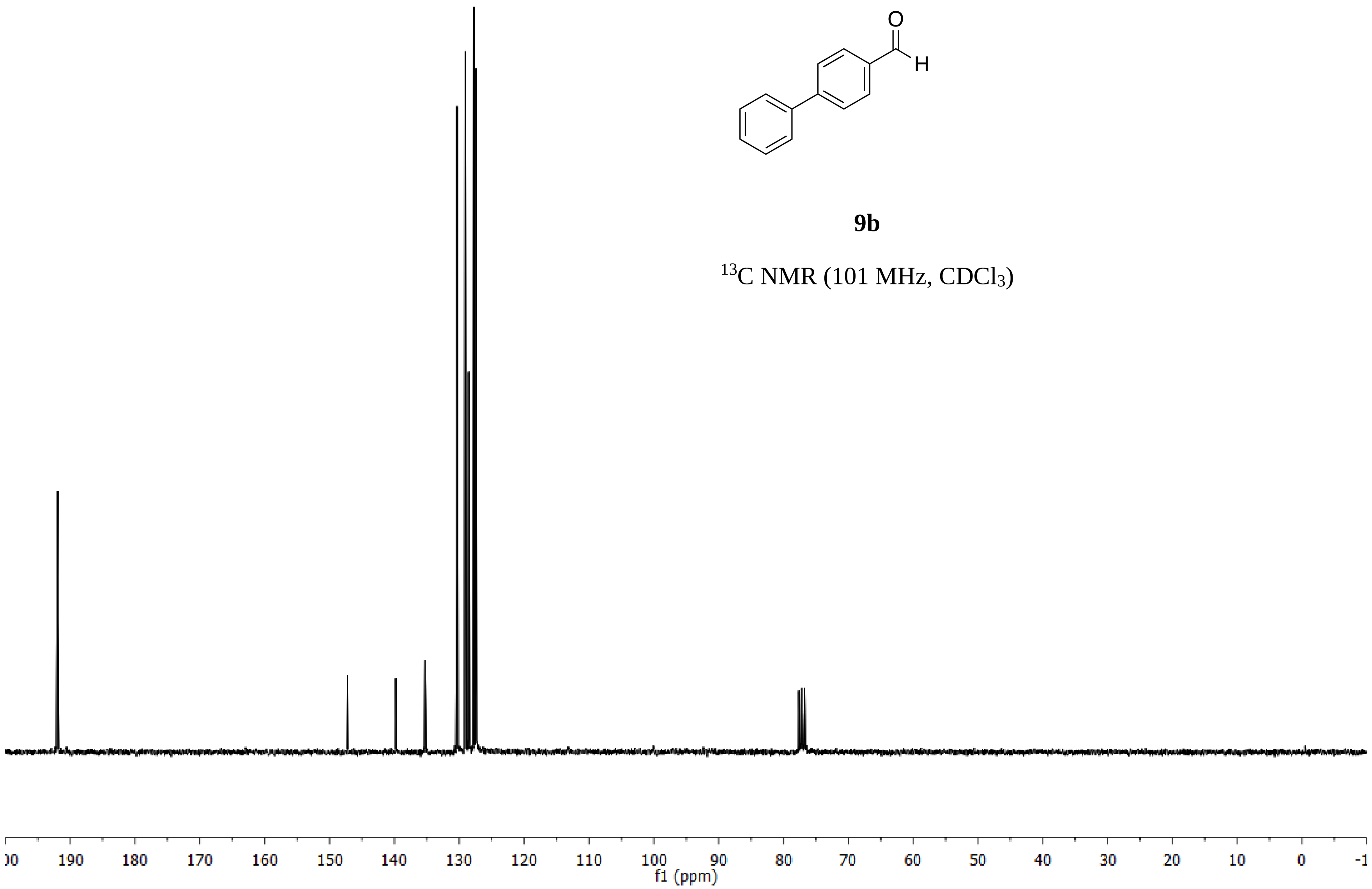

O
H
9b
^{13}C NMR (101 MHz, CDCl$_3$)
190
180
170
160
150
140
130
120
110
100
90
80
70
60
50
40
30
20
10
0
f1 (ppm)

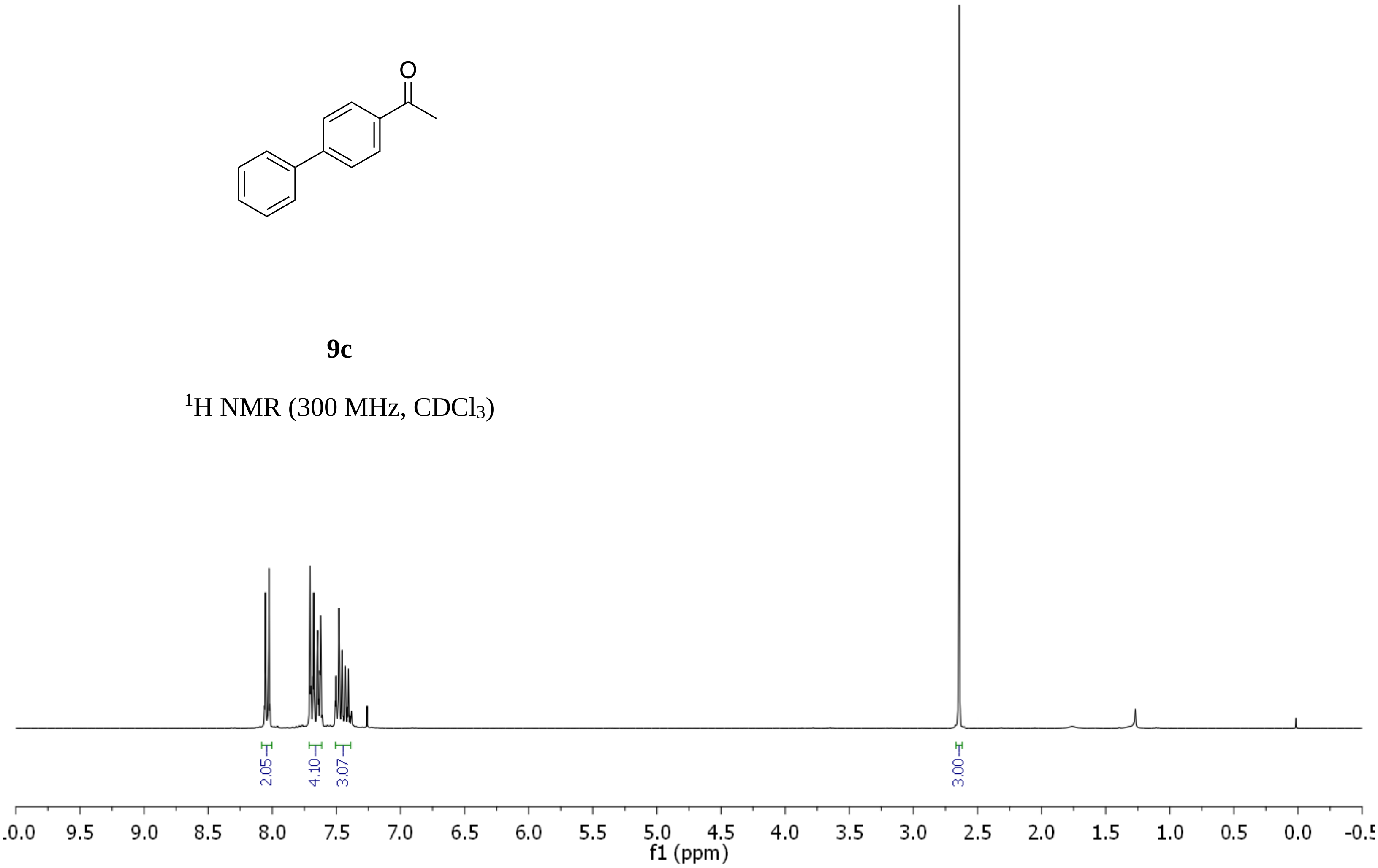

9c

^{1}H NMR (300 MHz, $CDCl_3$)

9c

^{13}C NMR (75 MHz, $CDCl_3$)

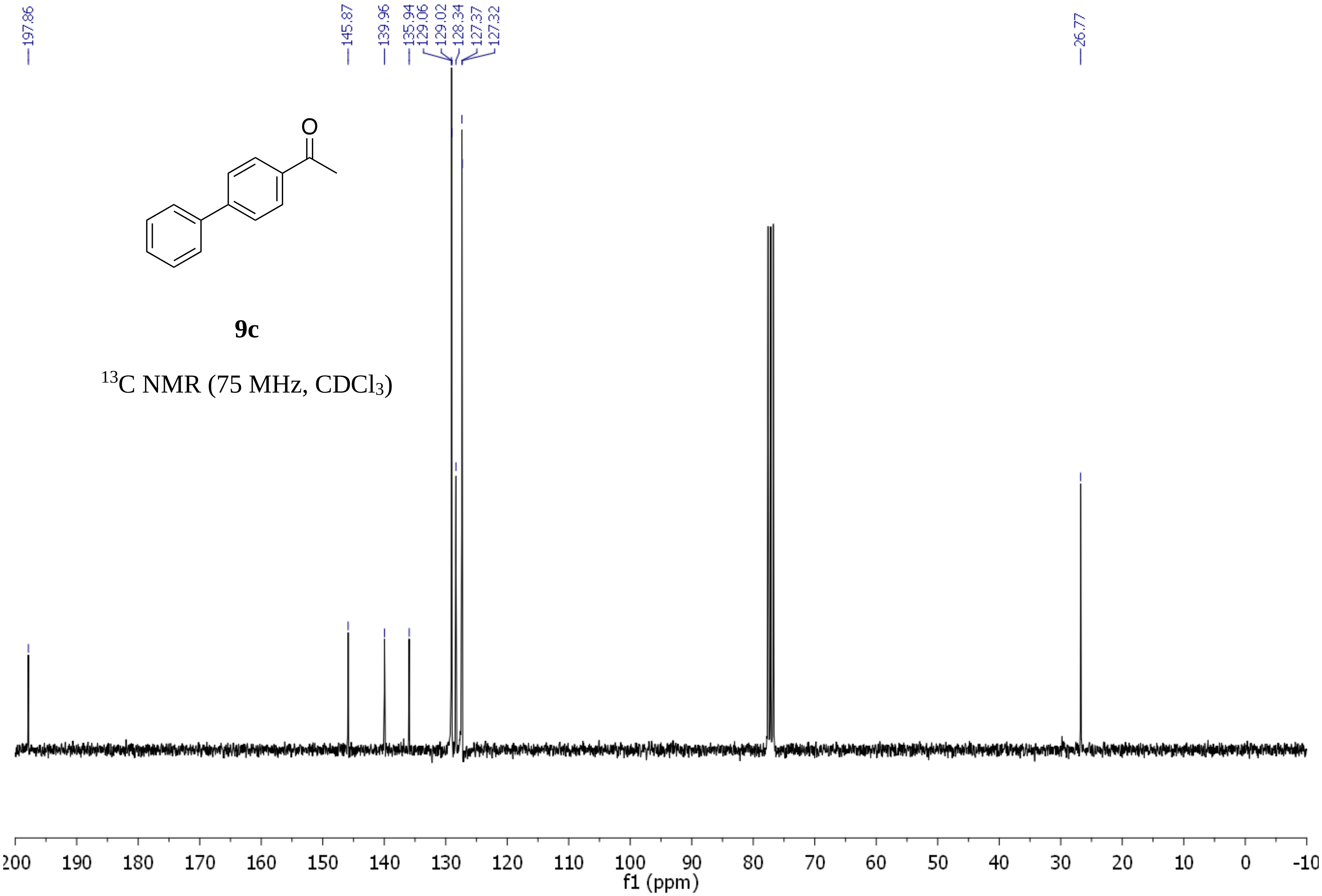

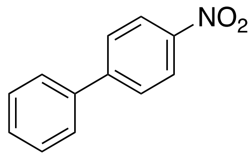

9d

^{1}H NMR (300 MHz, $CDCl_3$)

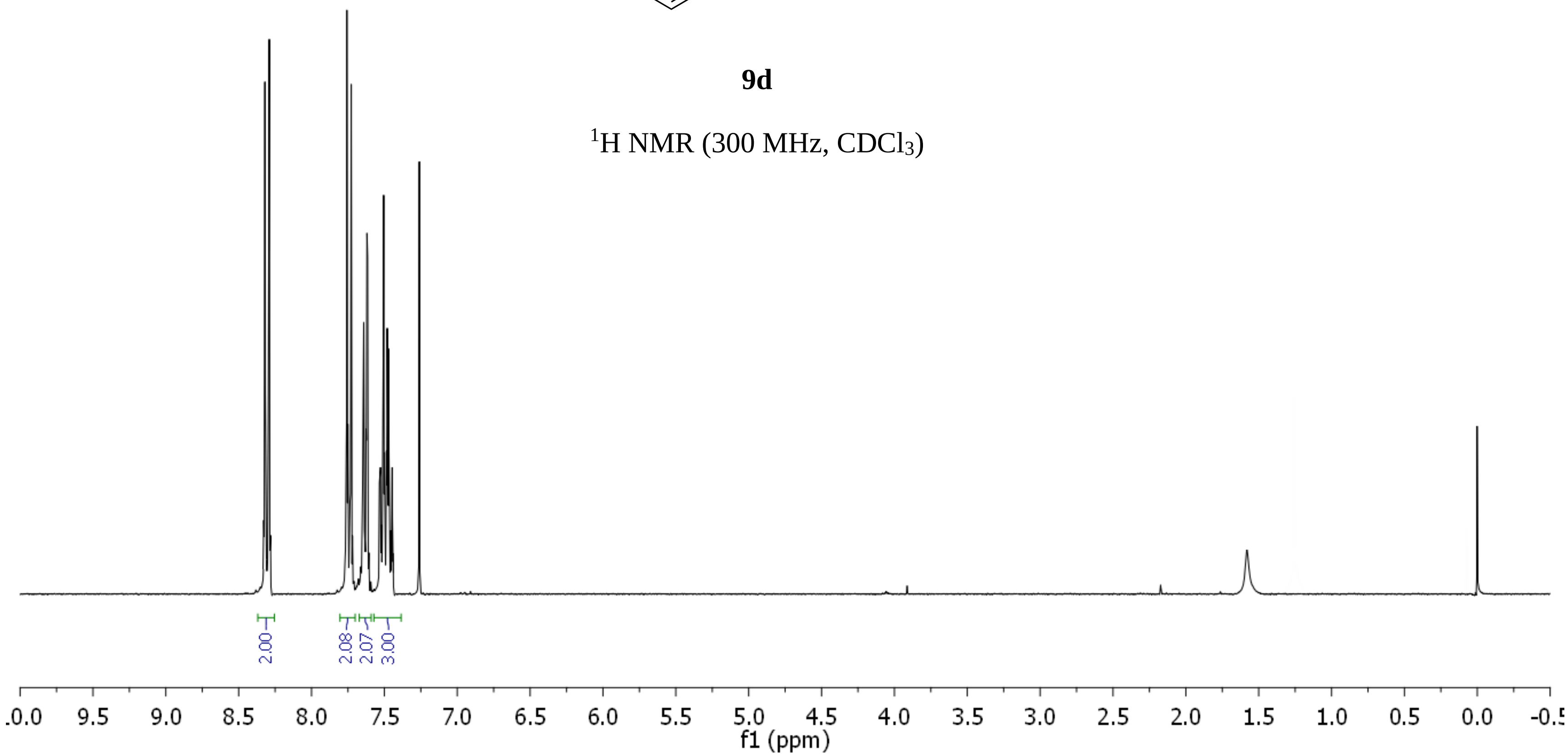

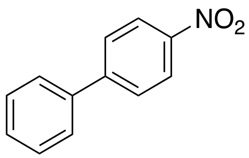

9d

^{13}C NMR (75 MHz, $CDCl_3$)

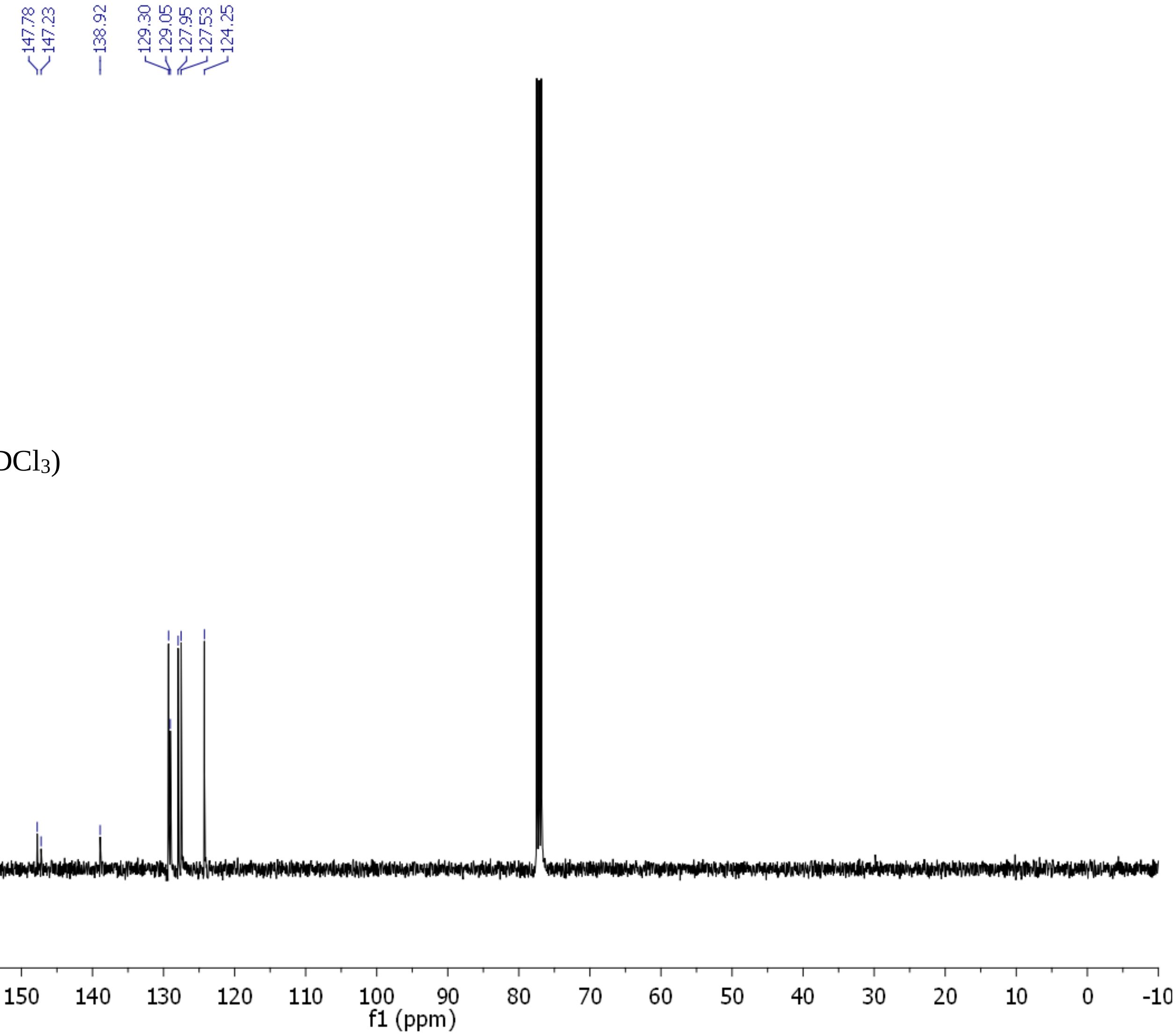

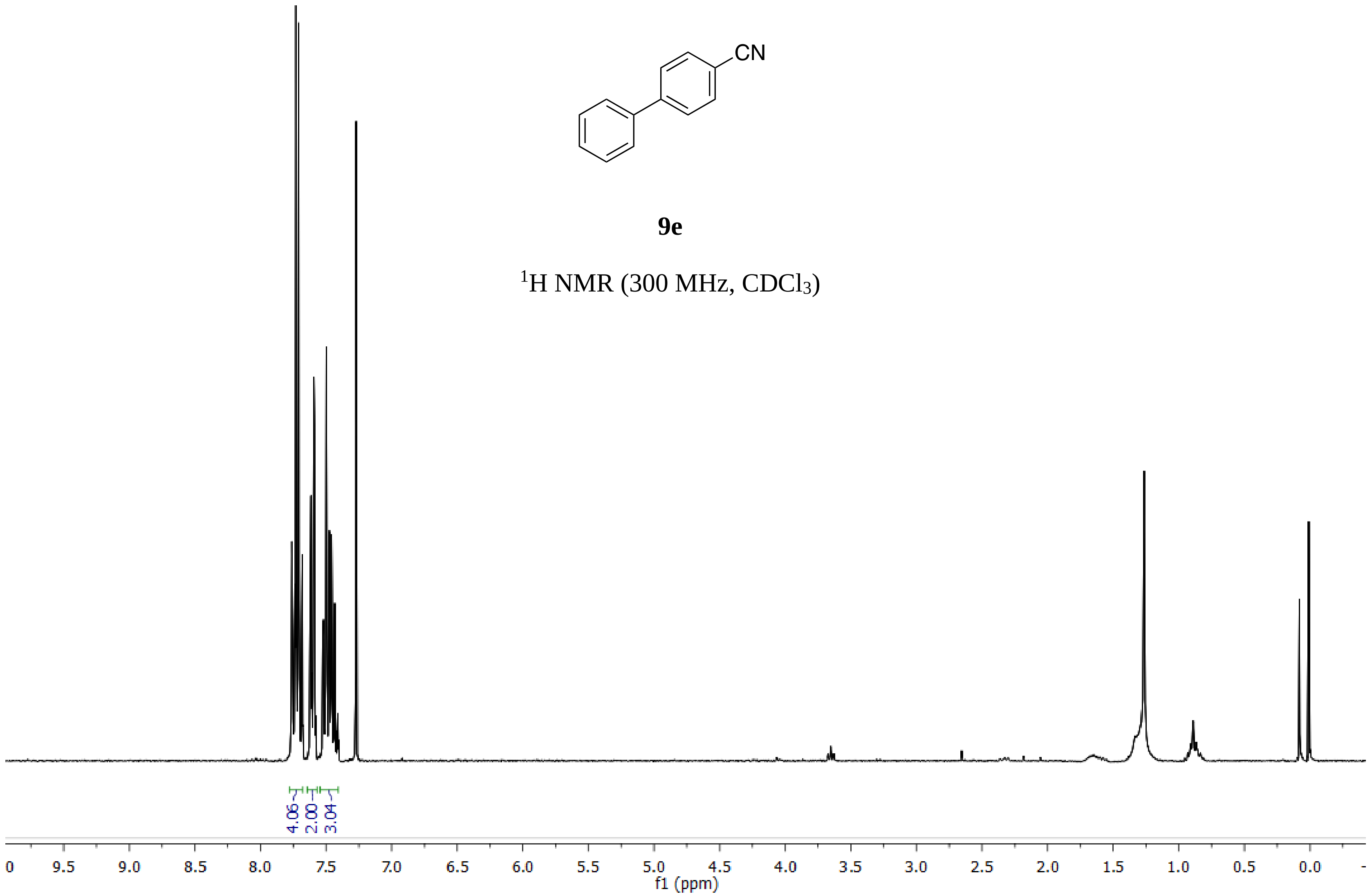

9e

^{1}H NMR (300 MHz, $CDCl_3$)

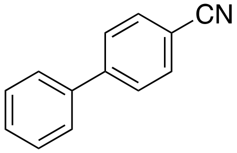

9e

^{13}C NMR (75 MHz, $CDCl_3$)

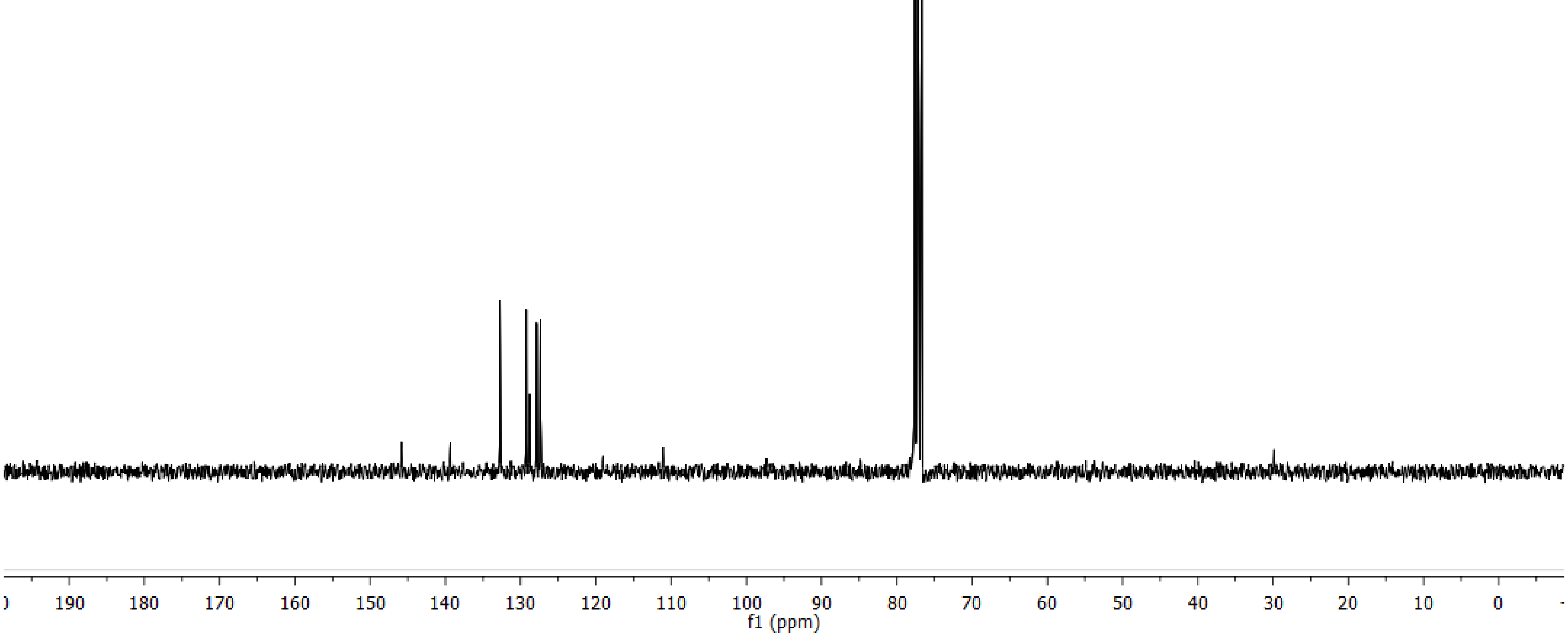

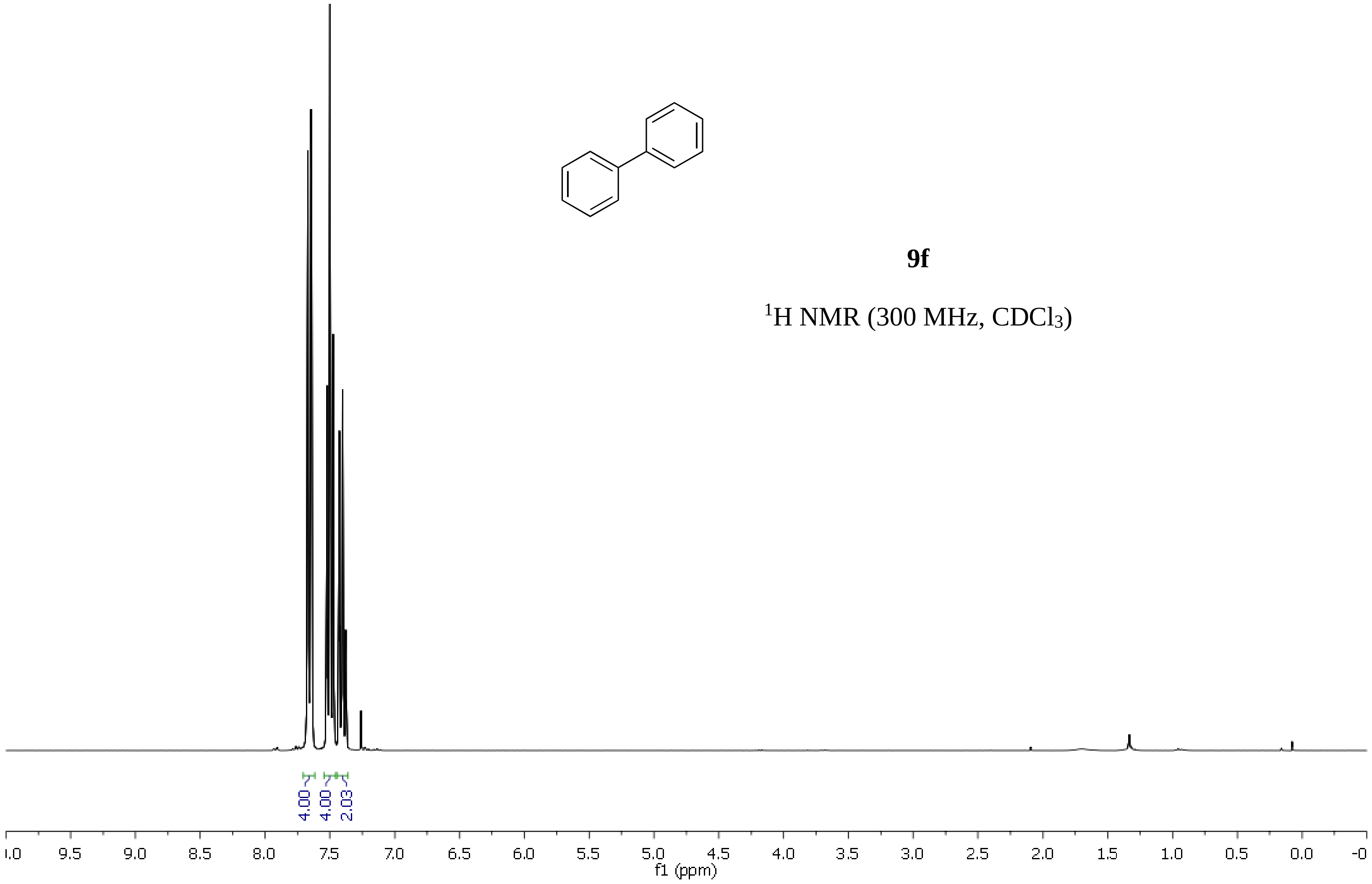

9f

^{1}H NMR (300 MHz, $CDCl_3$)

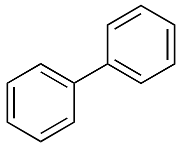

9f

^{13}C NMR (75 MHz, $CDCl_3$)

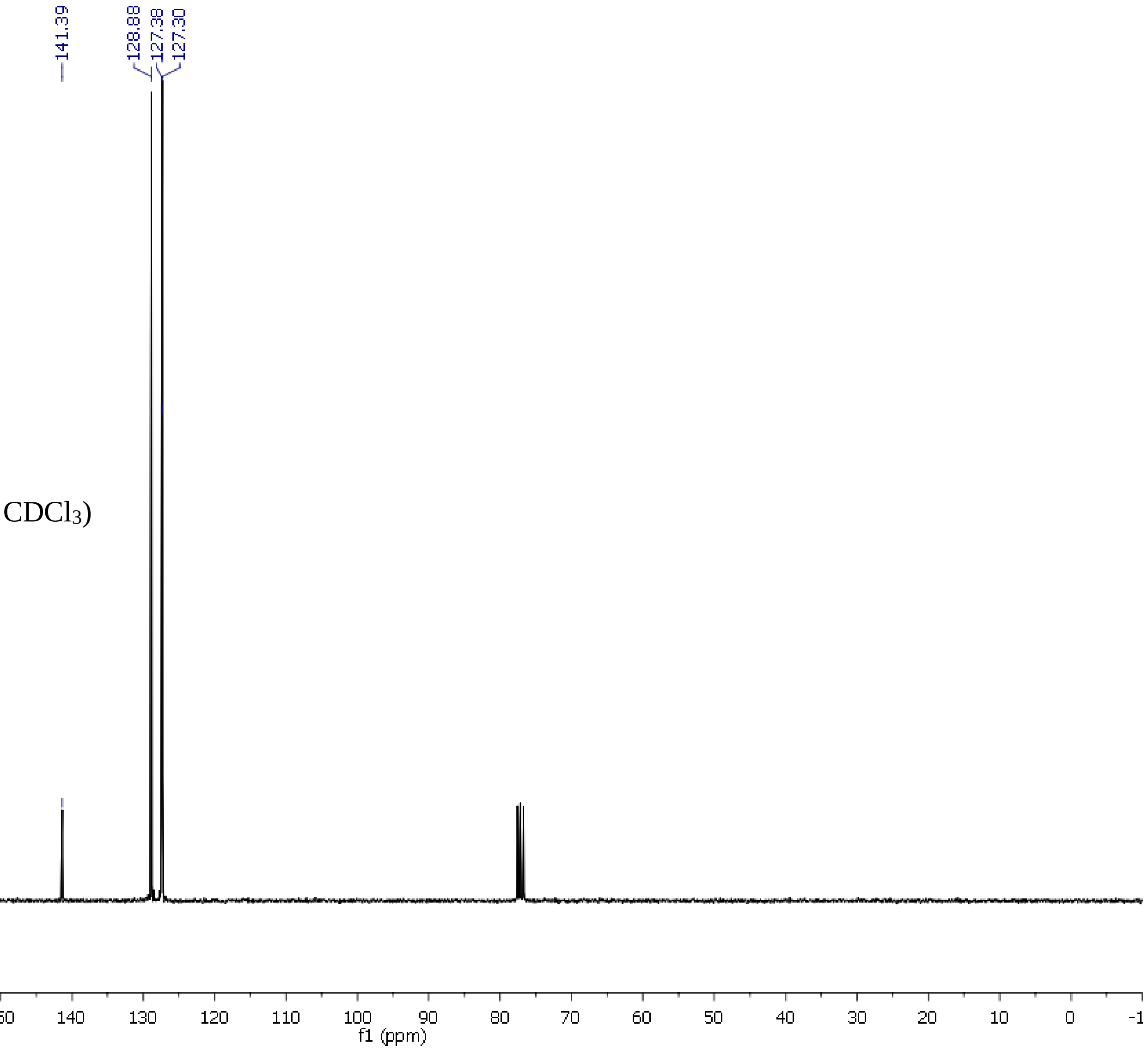

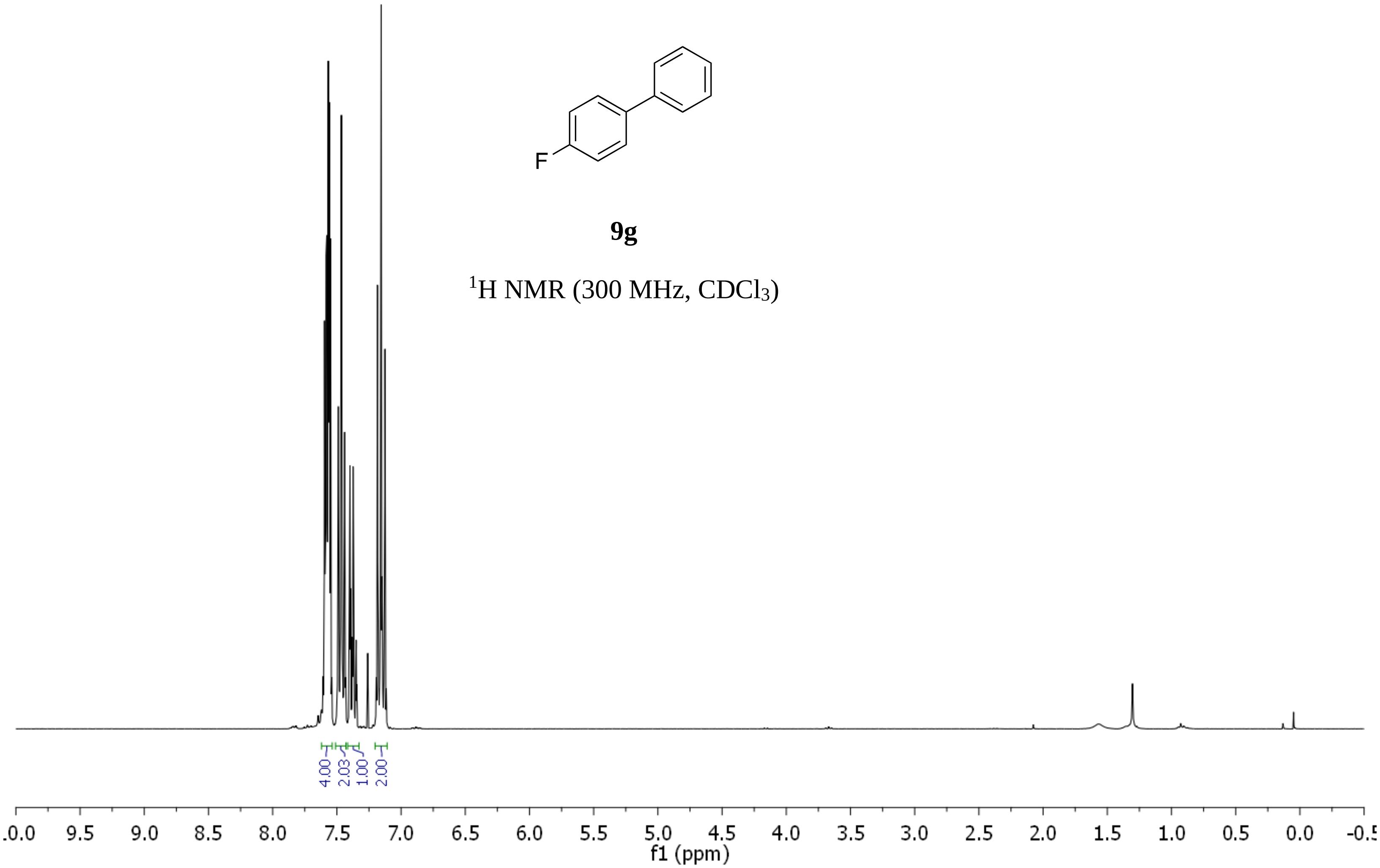

9g

^{1}H NMR (300 MHz, $CDCl_3$)

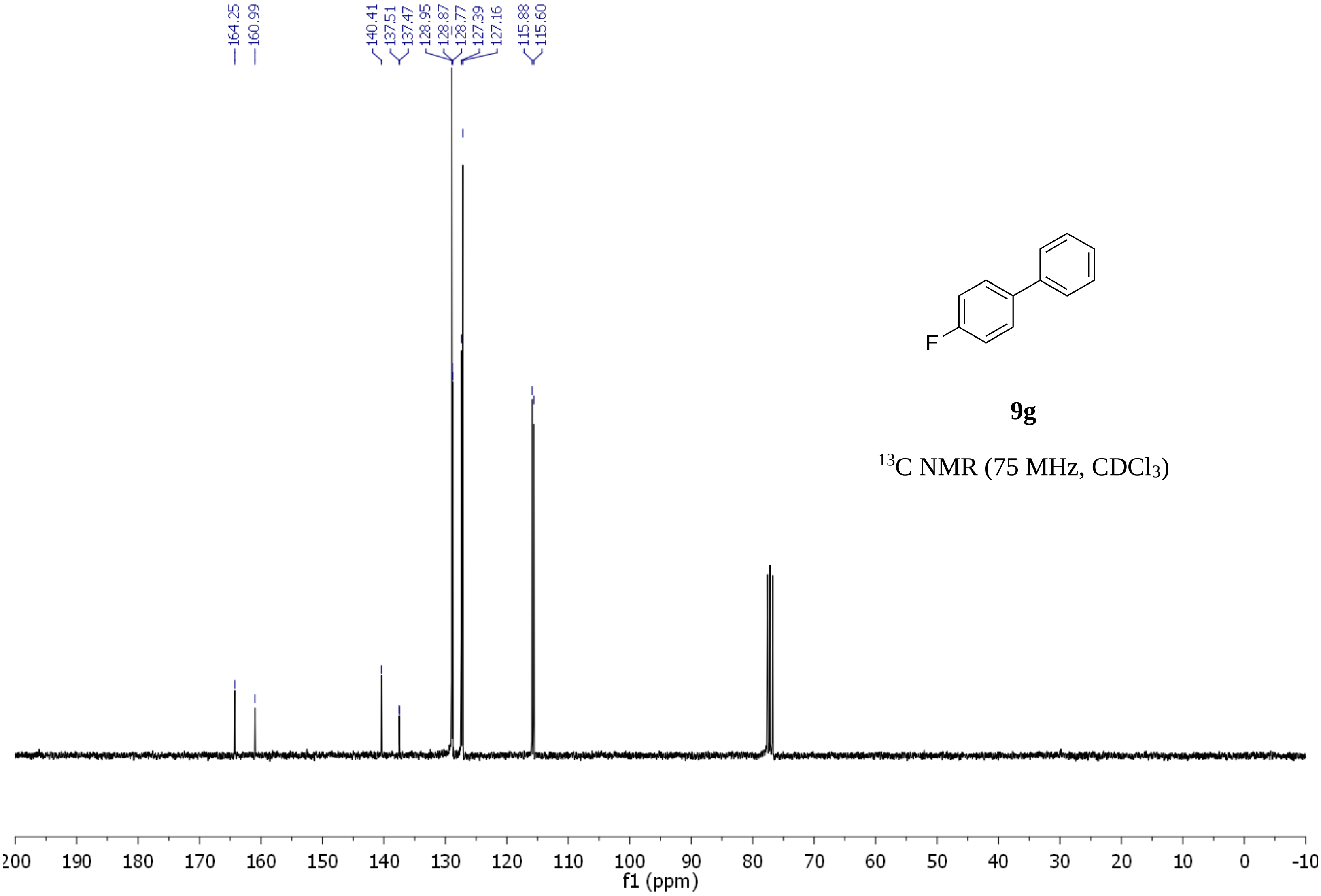

9g

^{13}C NMR (75 MHz, $CDCl_3$)

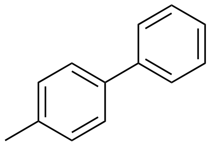

9h

^{1}H NMR (300 MHz, $CDCl_3$)

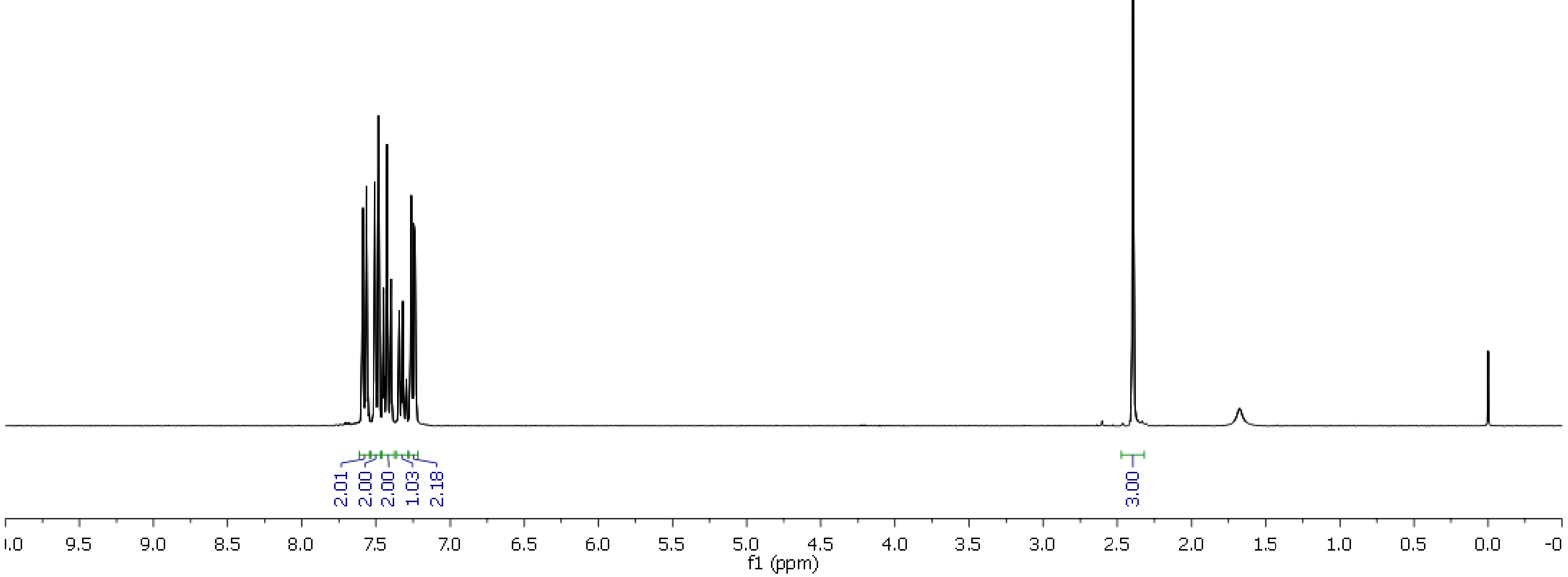

9h

^{13}C NMR (75 MHz, $CDCl_3$)

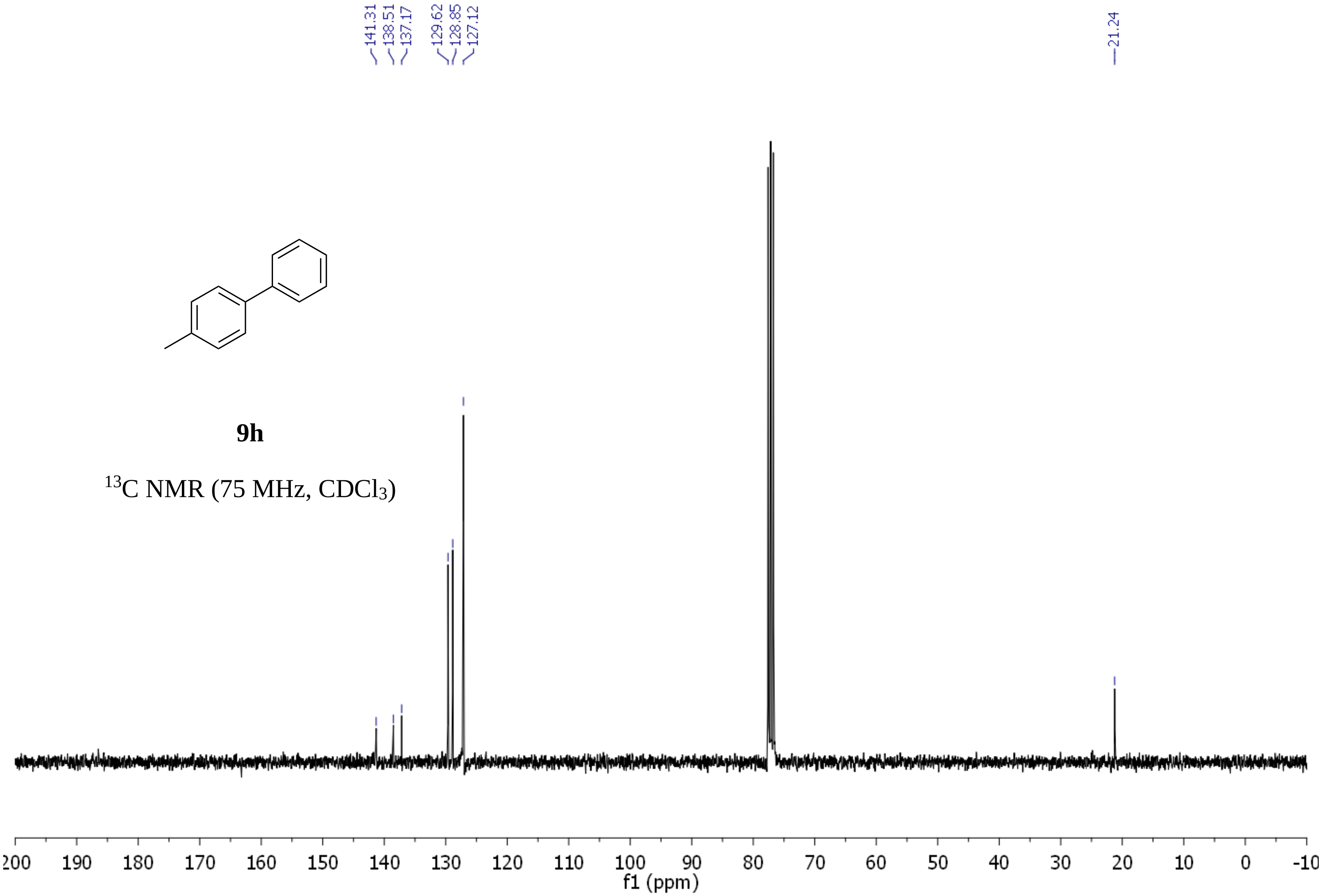

9i

^{1}H NMR (300 MHz, $CDCl_3$)

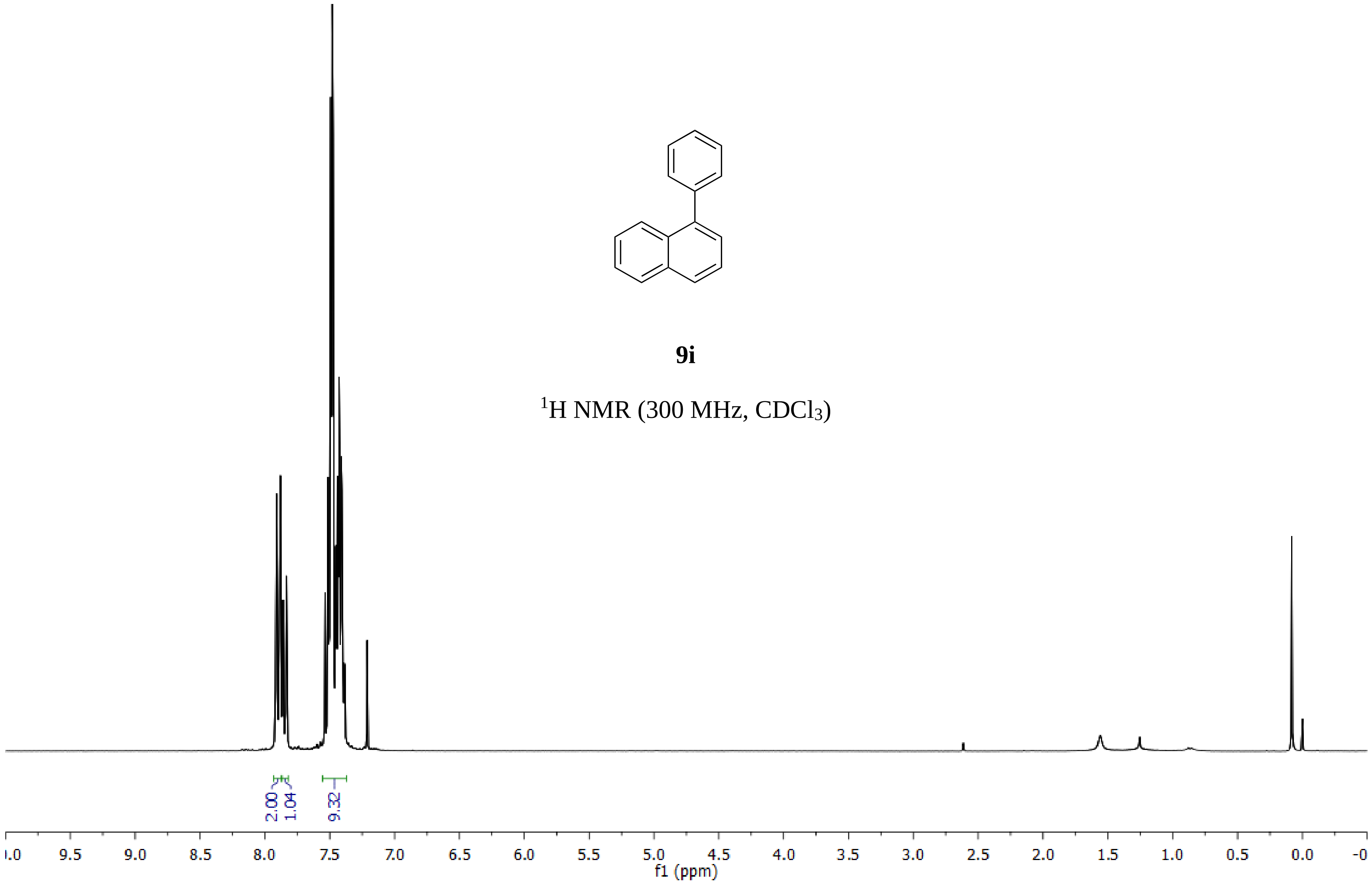

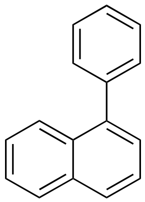

9i

^{13}C NMR (75 MHz, $CDCl_3$)

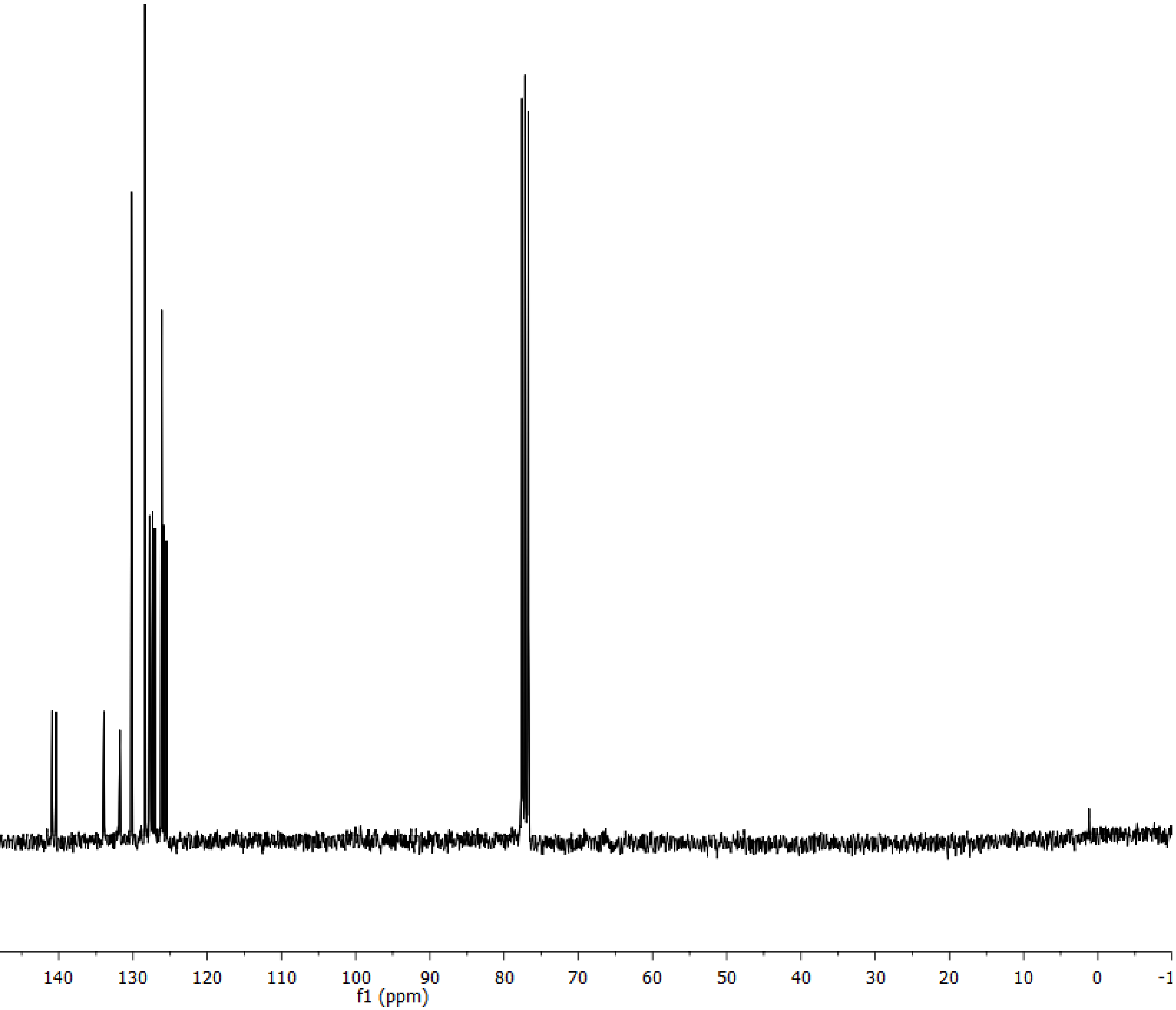

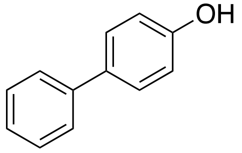

9j

^{1}H NMR (300 MHz, $CDCl_3$)

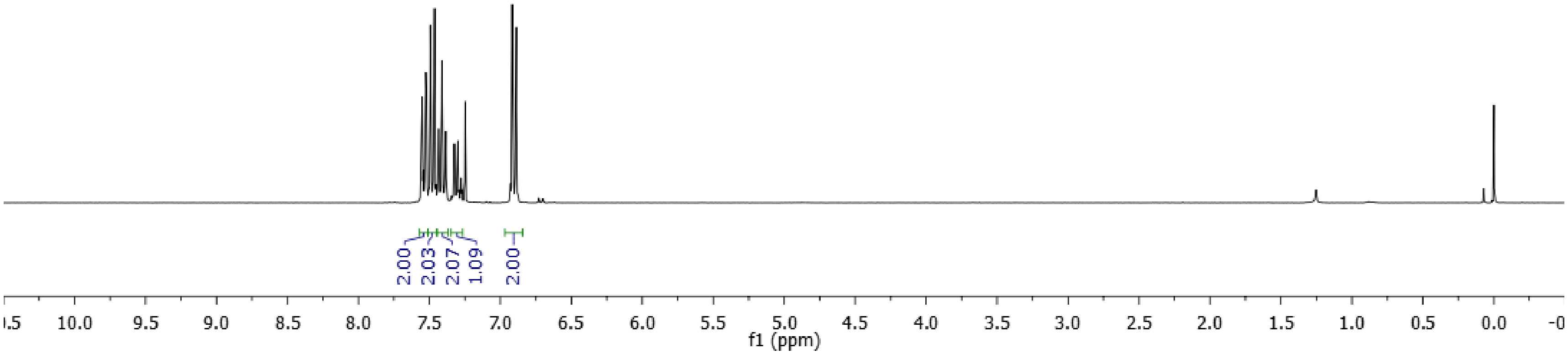

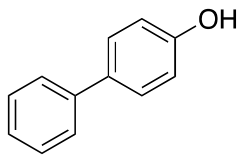

9j

^{13}C NMR (75 MHz, $CDCl_3$)

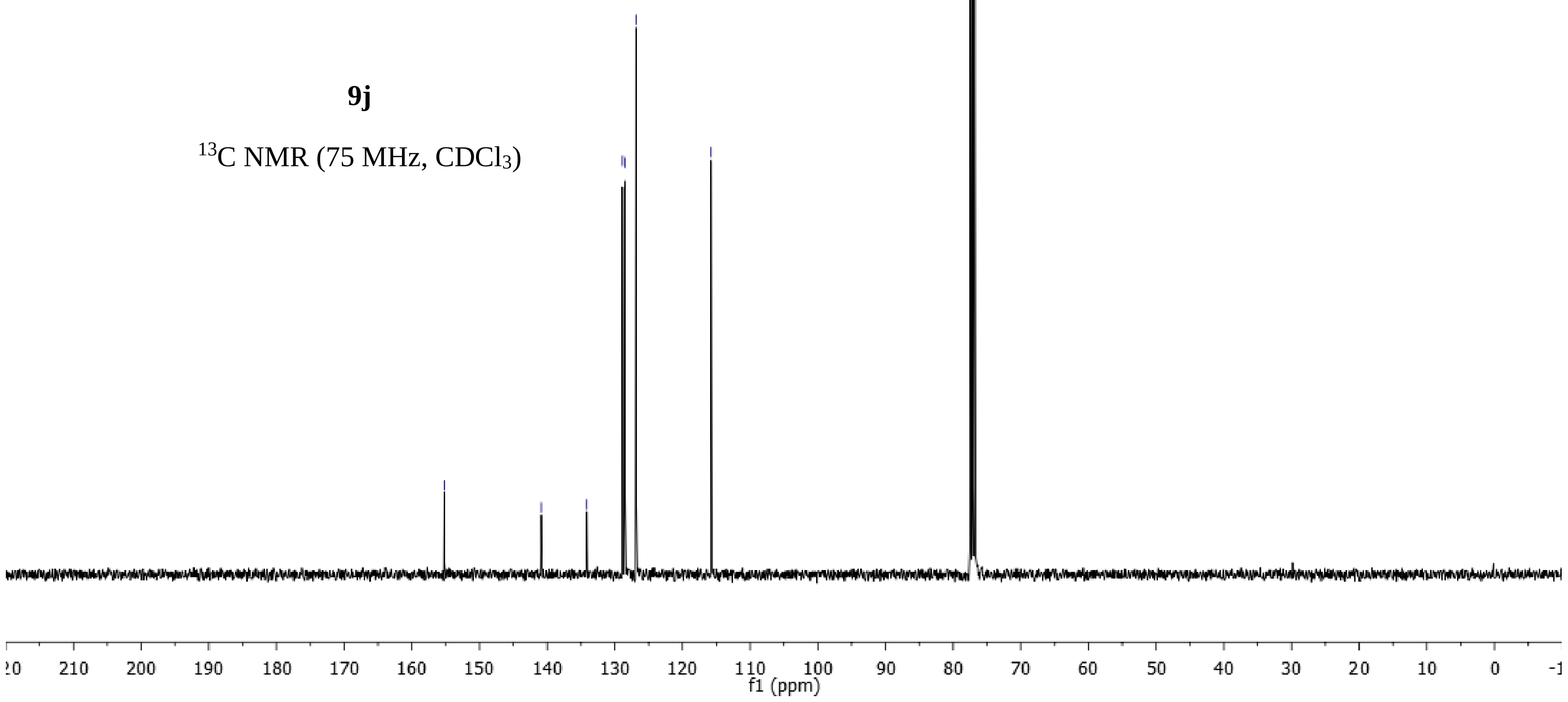

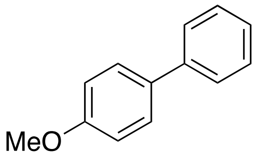

9k

^{1}H NMR (300 MHz, $CDCl_3$)

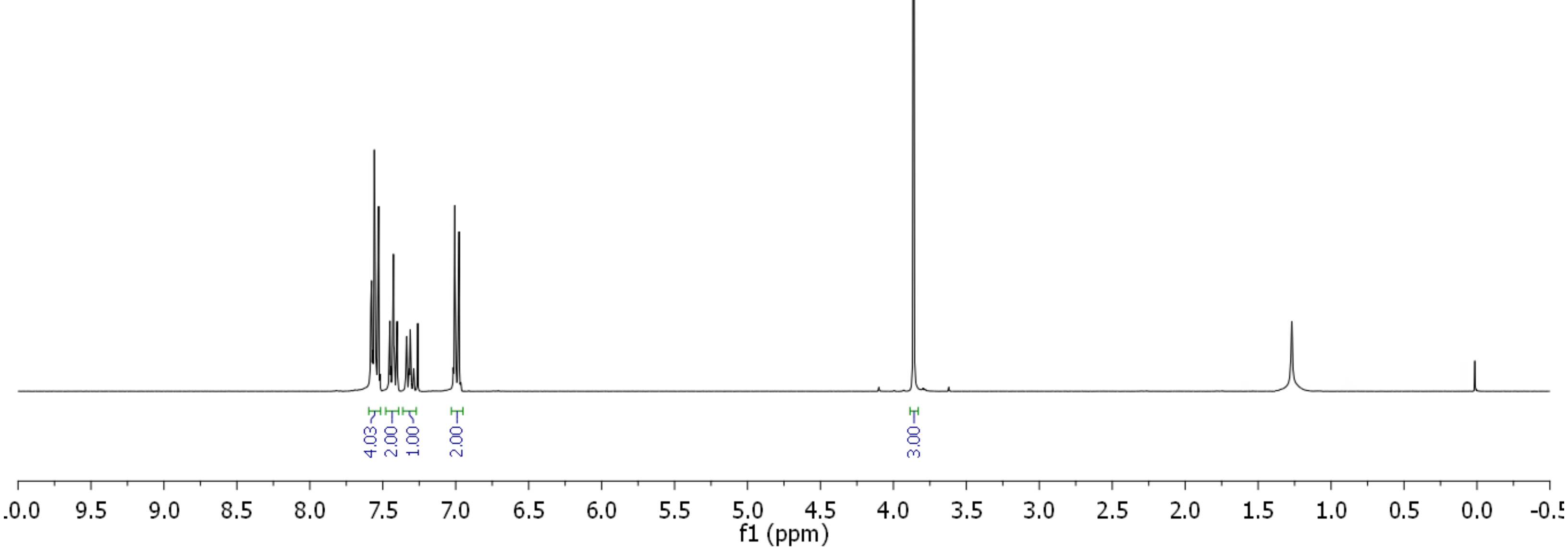

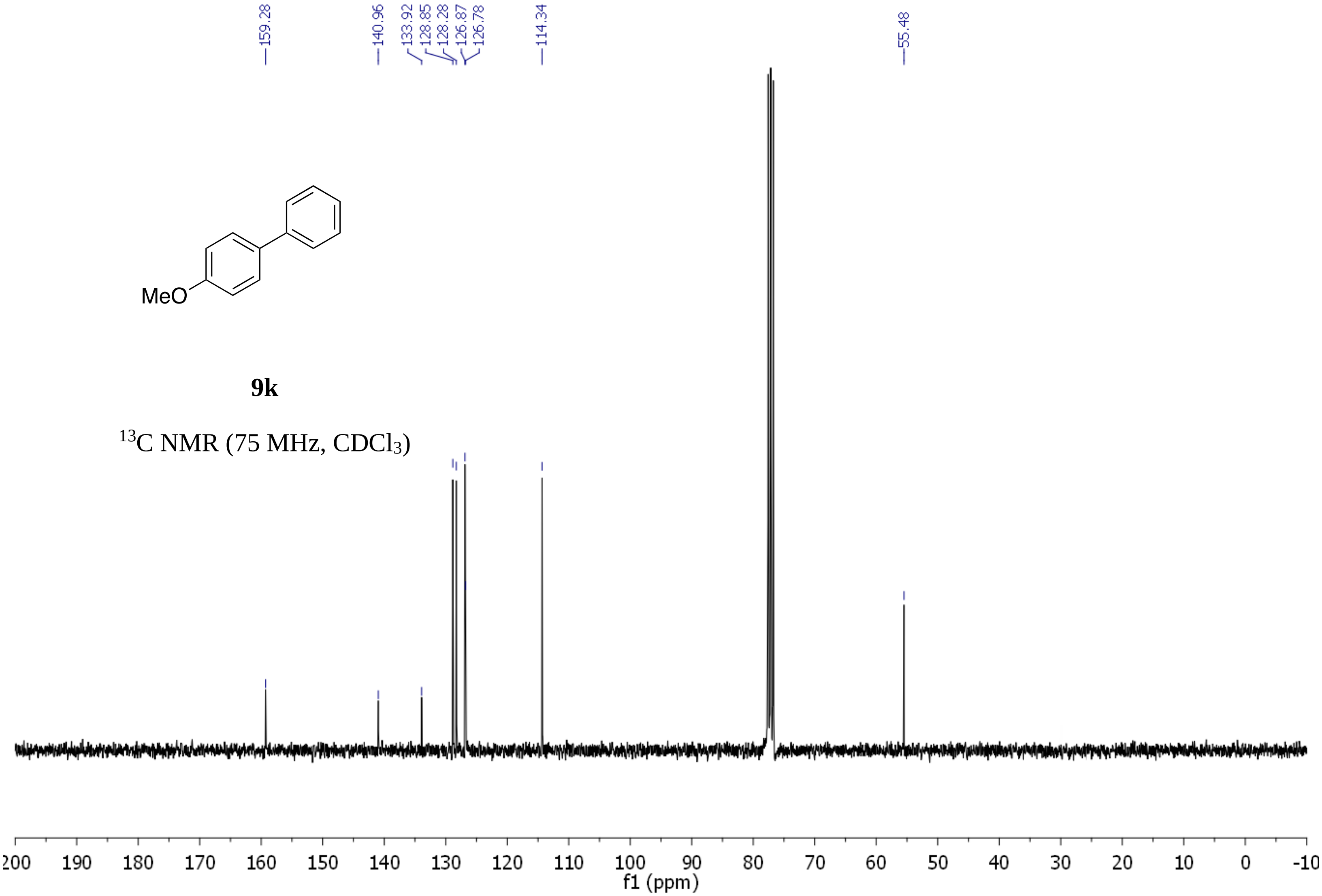

9k

^{13}C NMR (75 MHz, $CDCl_3$)

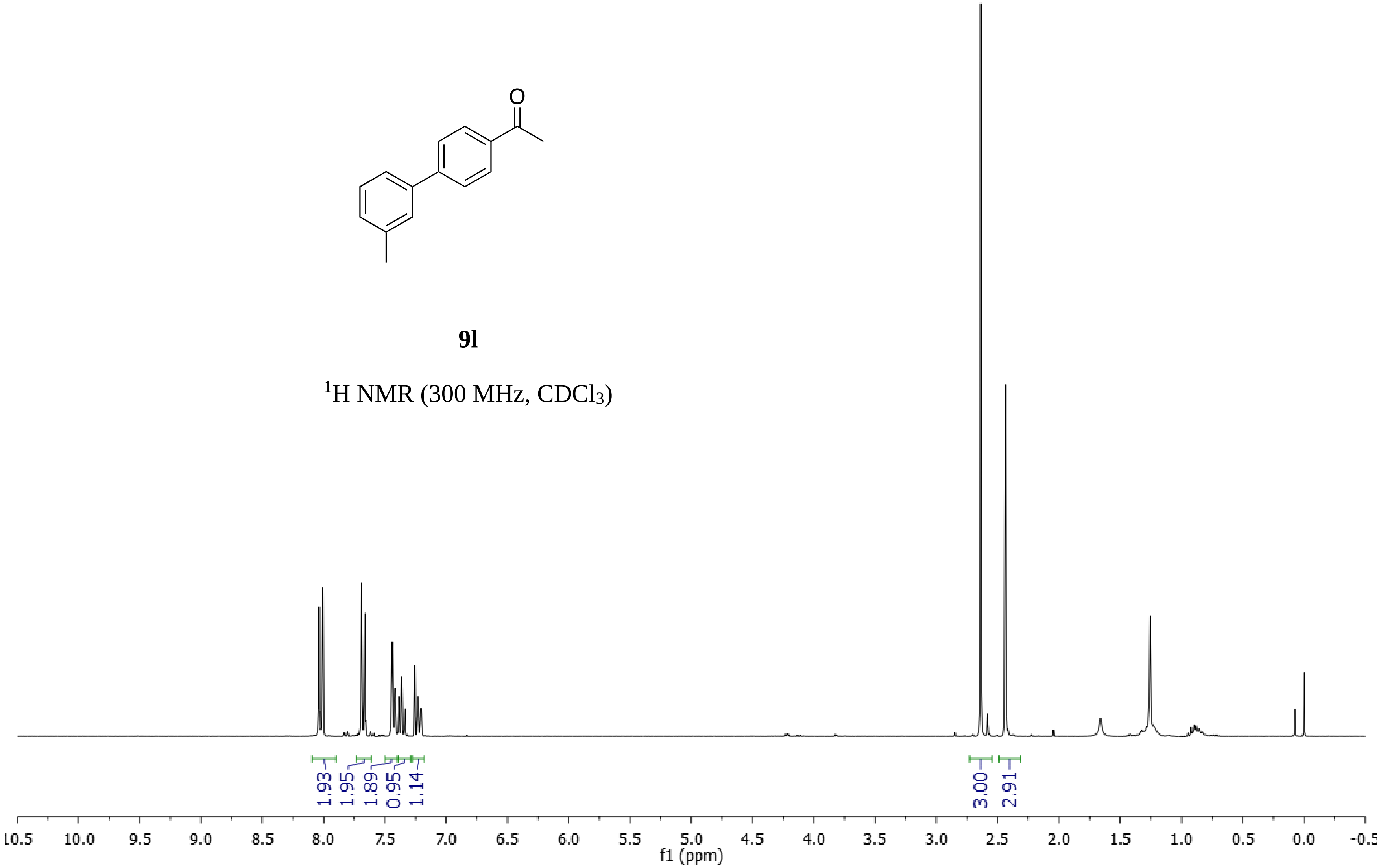

9l

^{1}H NMR (300 MHz, $CDCl_3$)

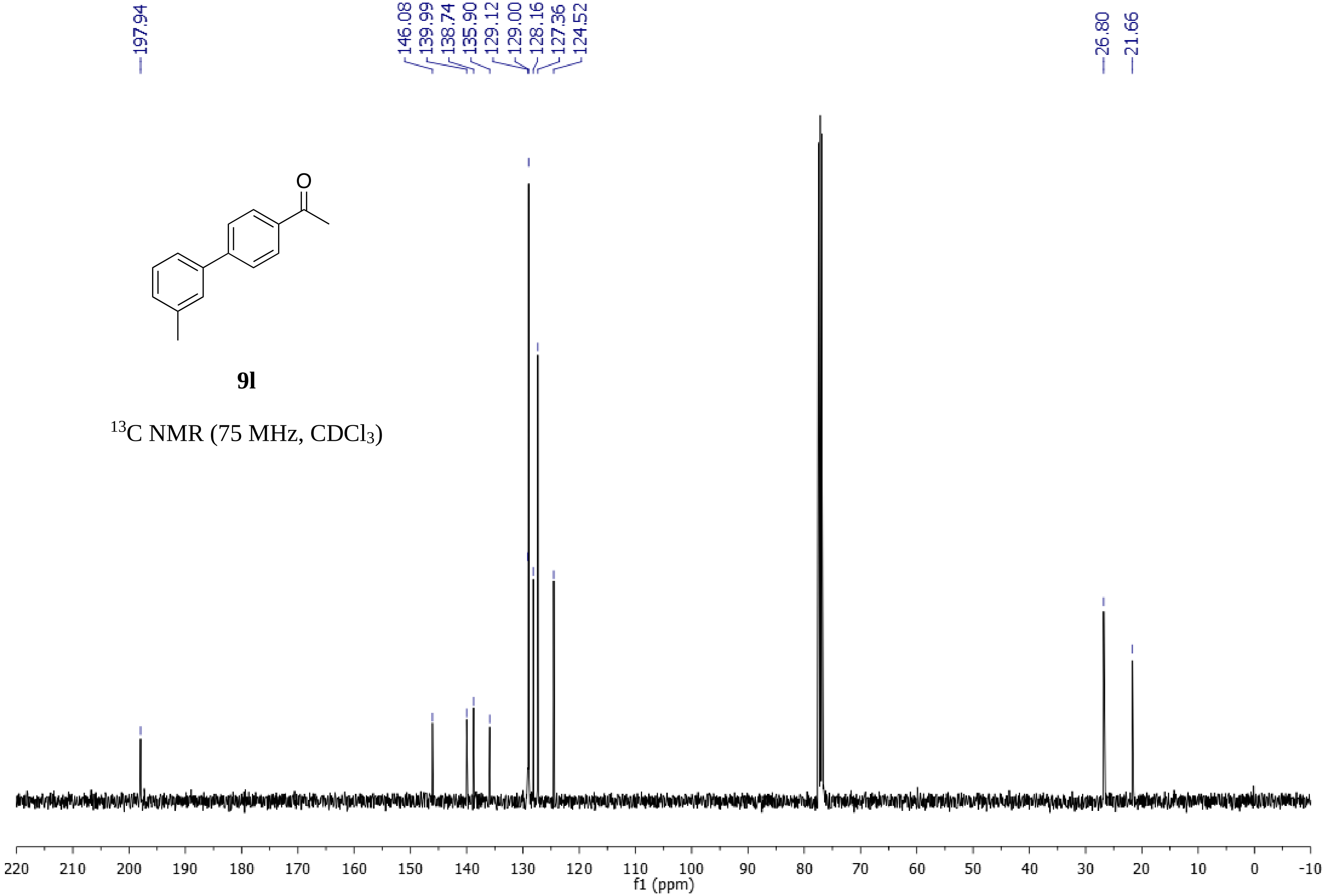
197.94
146.08
139.99
138.74
135.90
129.12
129.00
128.16
127.36
124.52
26.80
21.66
9l
13C NMR (75 MHz, CDCl3)
220 210 200 190 180 170 160 150 140 130 120 110 100 90 80 70 60 50 40 30 20 10 0 -10
f1 (ppm)

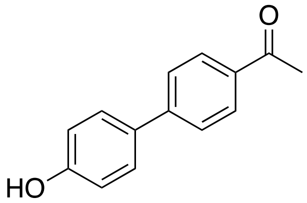

9m

^{1}H NMR (300 MHz, $CDCl_3$)

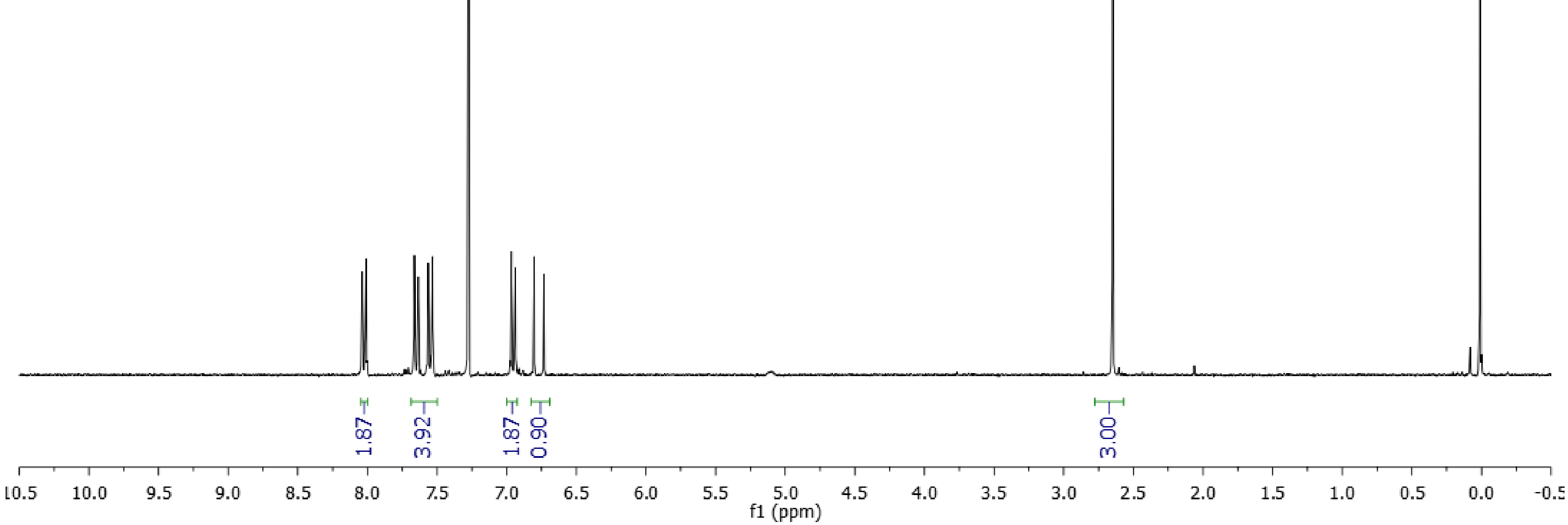

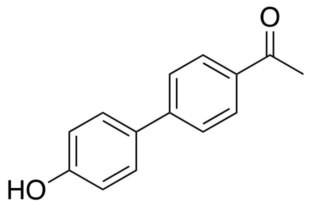

9m

^{13}C NMR (75 MHz, MeOD)

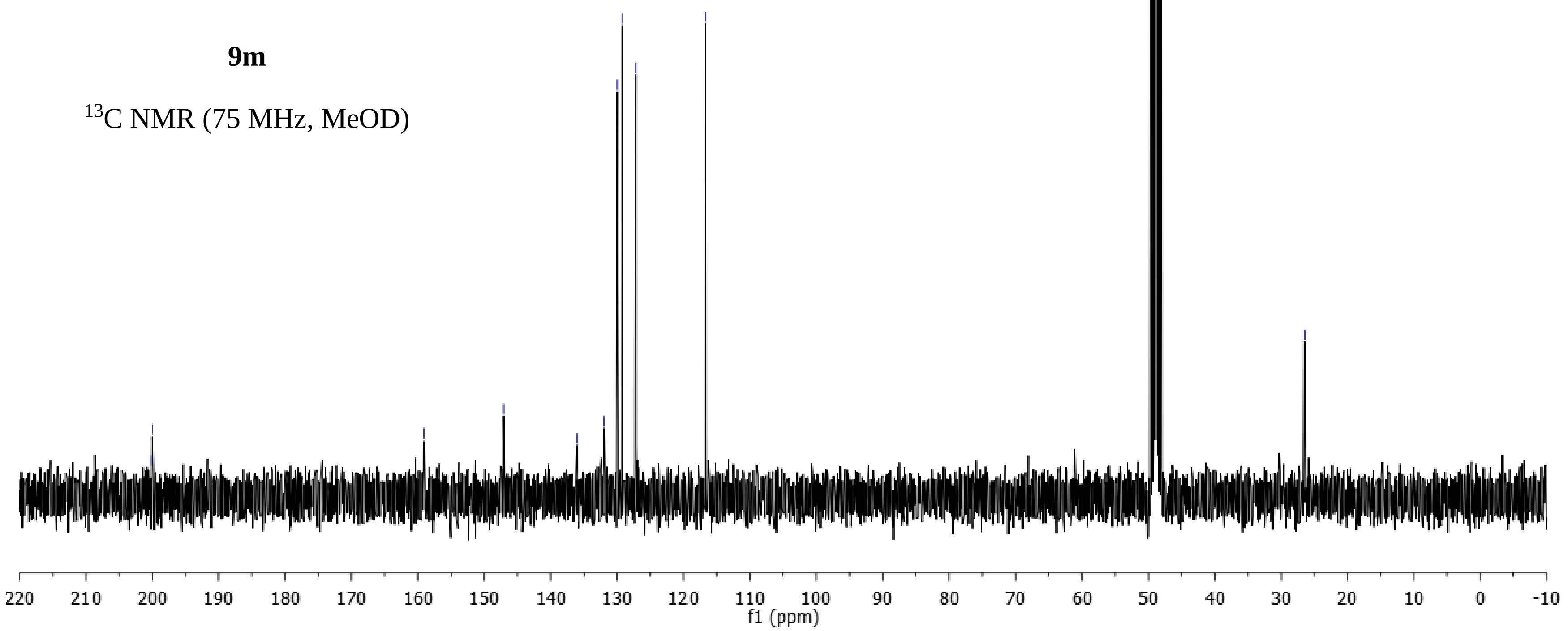

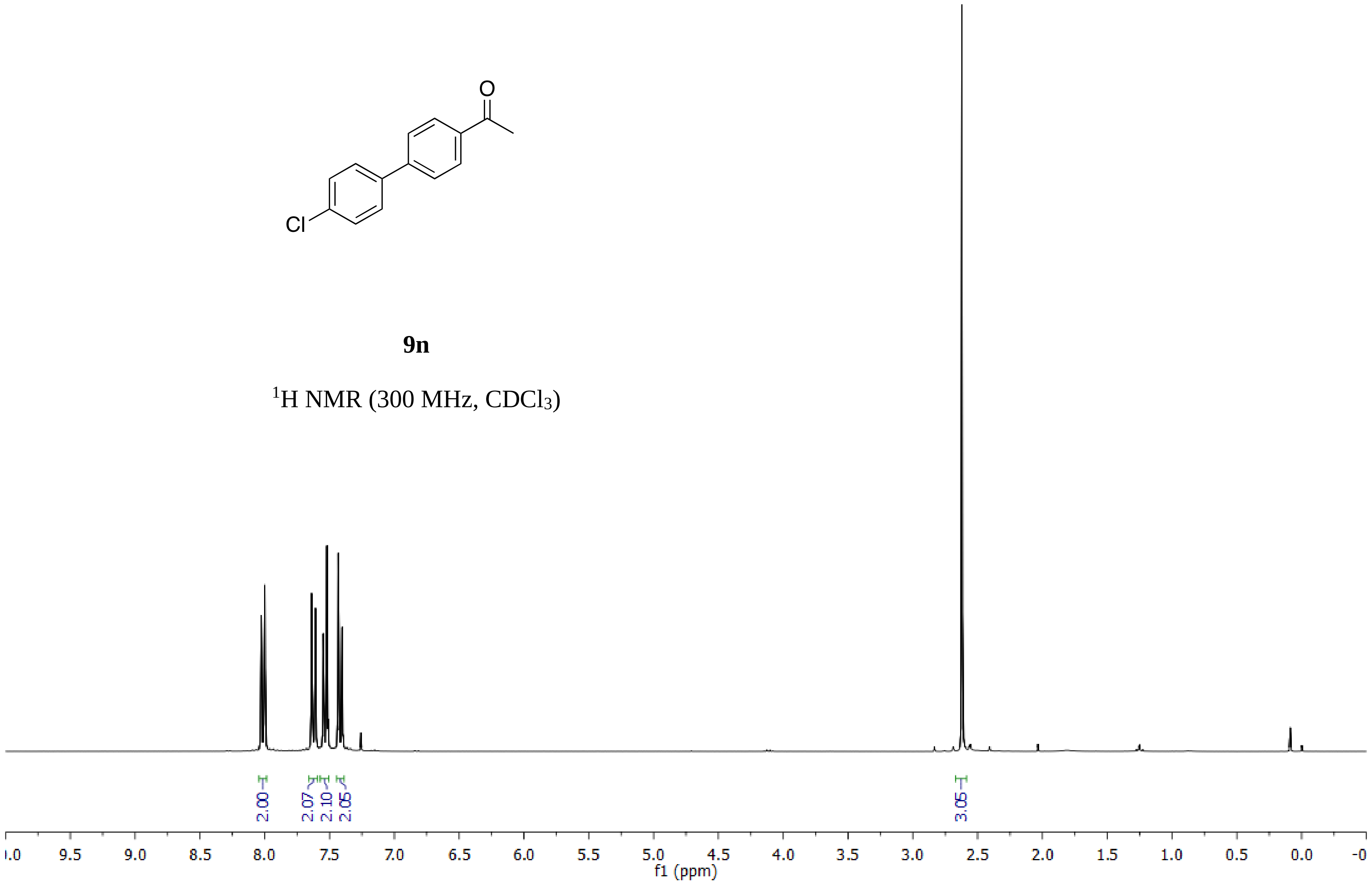

9n

^{1}H NMR (300 MHz, $CDCl_3$)

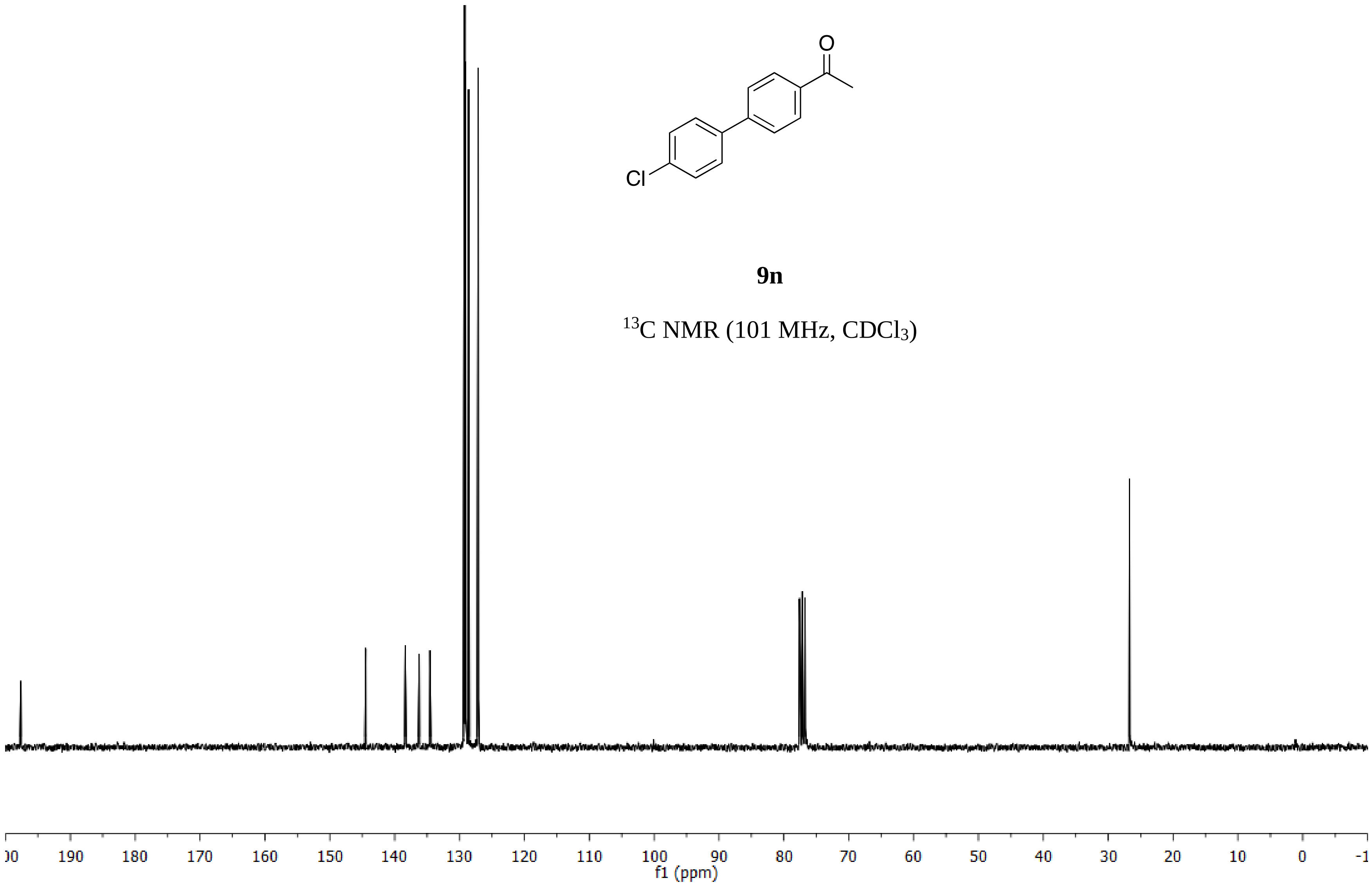

9n

^{13}C NMR (101 MHz, $CDCl_3$)

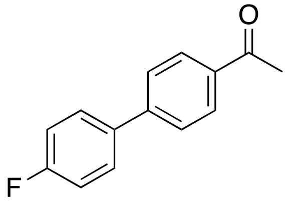

9o

^{1}H NMR (300 MHz, $CDCl_3$)

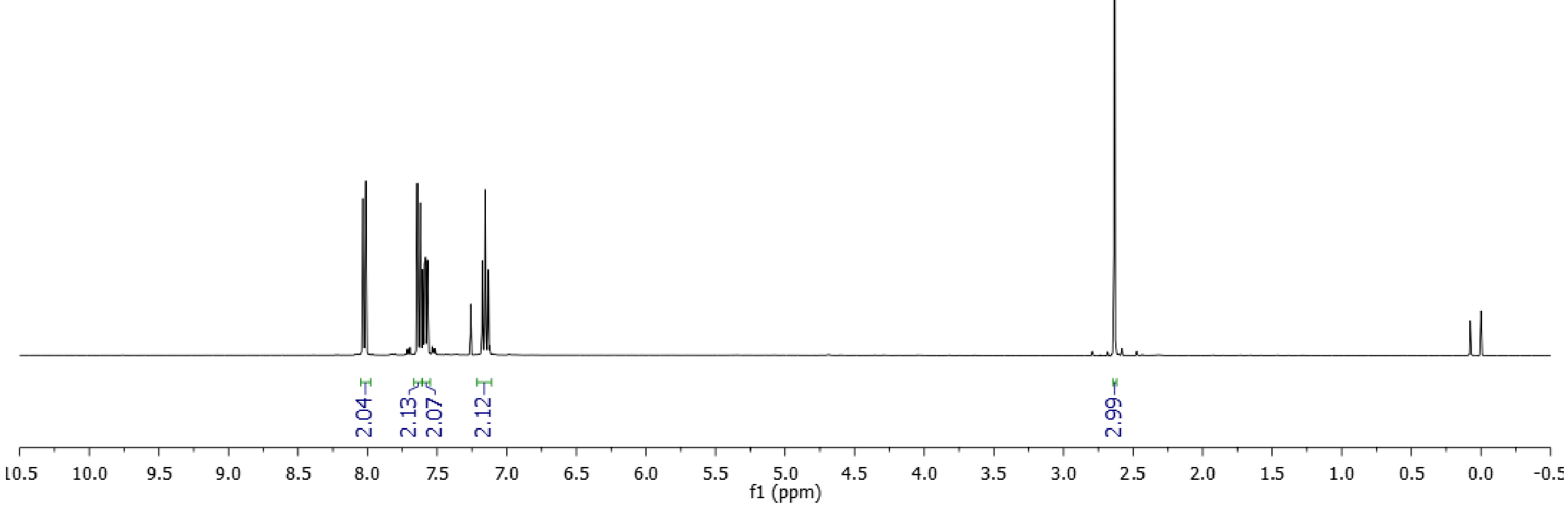

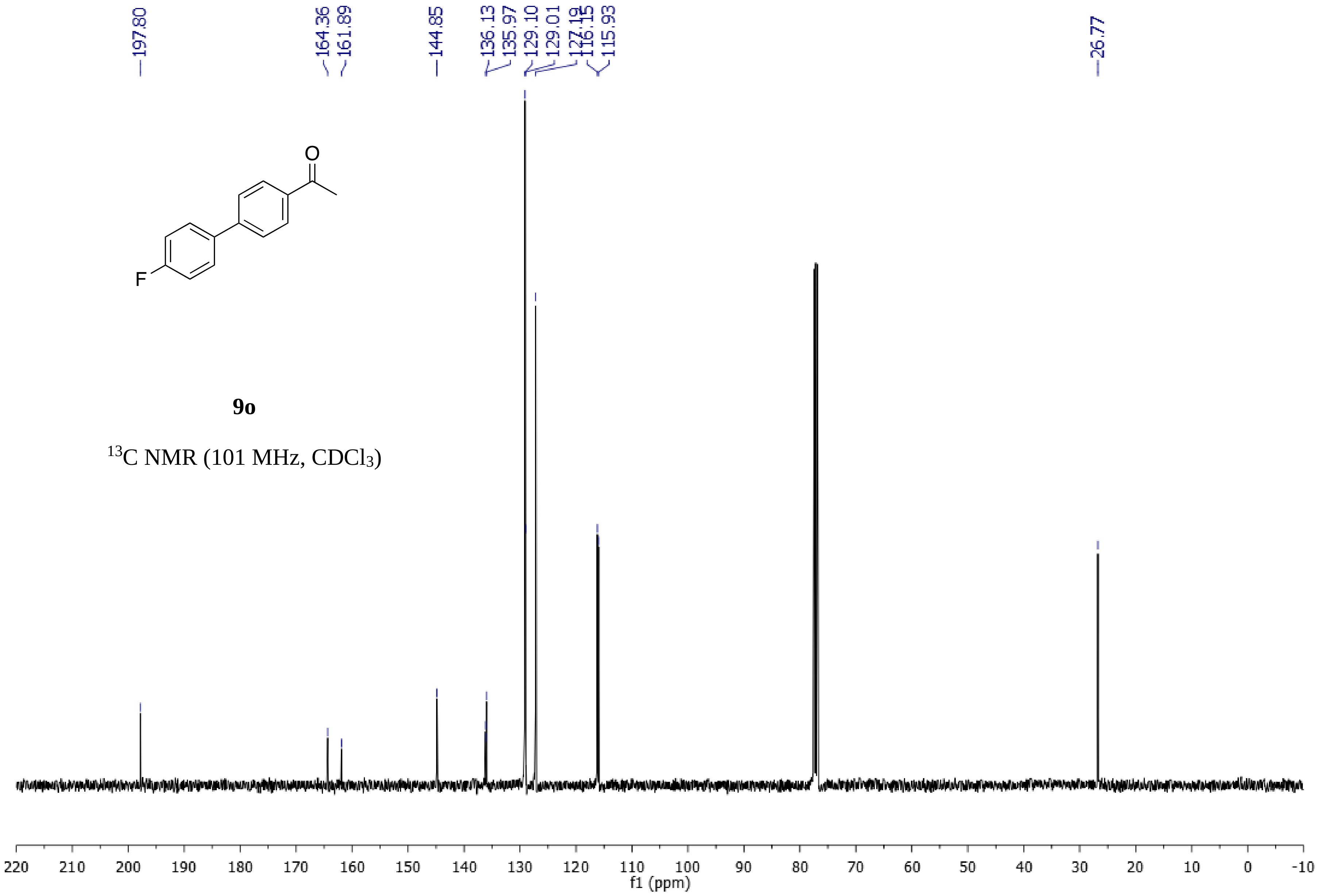

9o

^{13}C NMR (101 MHz, CDCl$_3$)

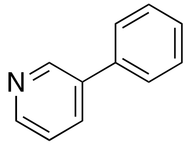

9p

^{1}H NMR (300 MHz, $CDCl_3$)

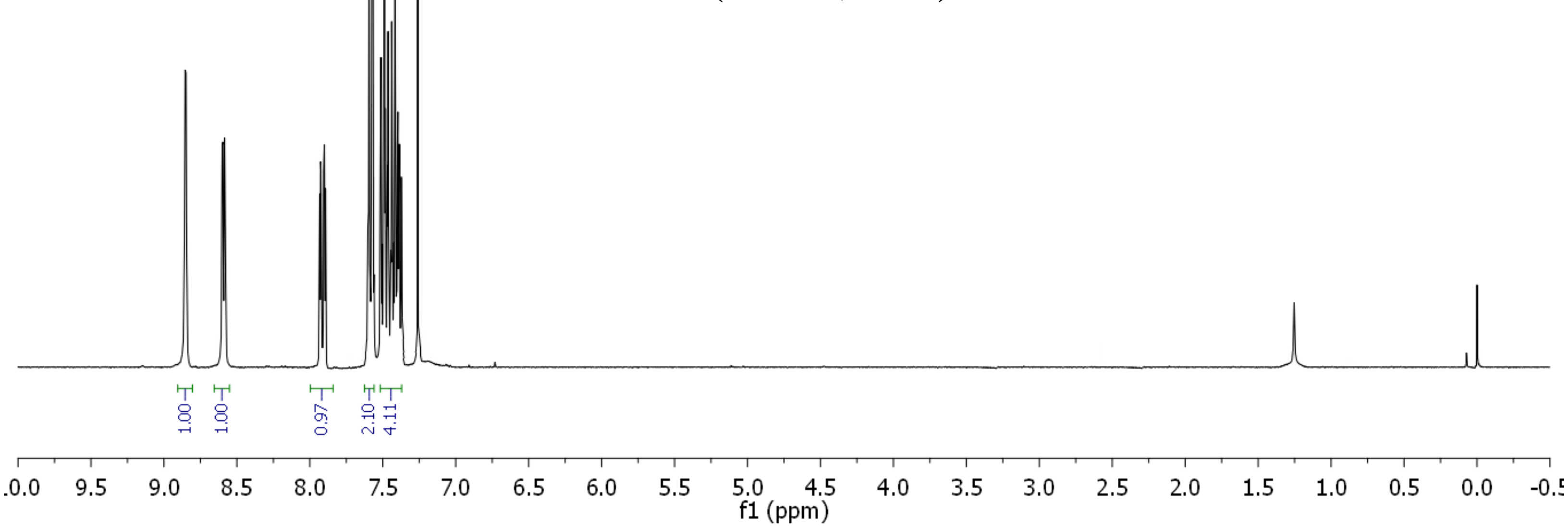

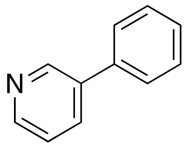

9p

^{13}C NMR (75 MHz, $CDCl_3$)

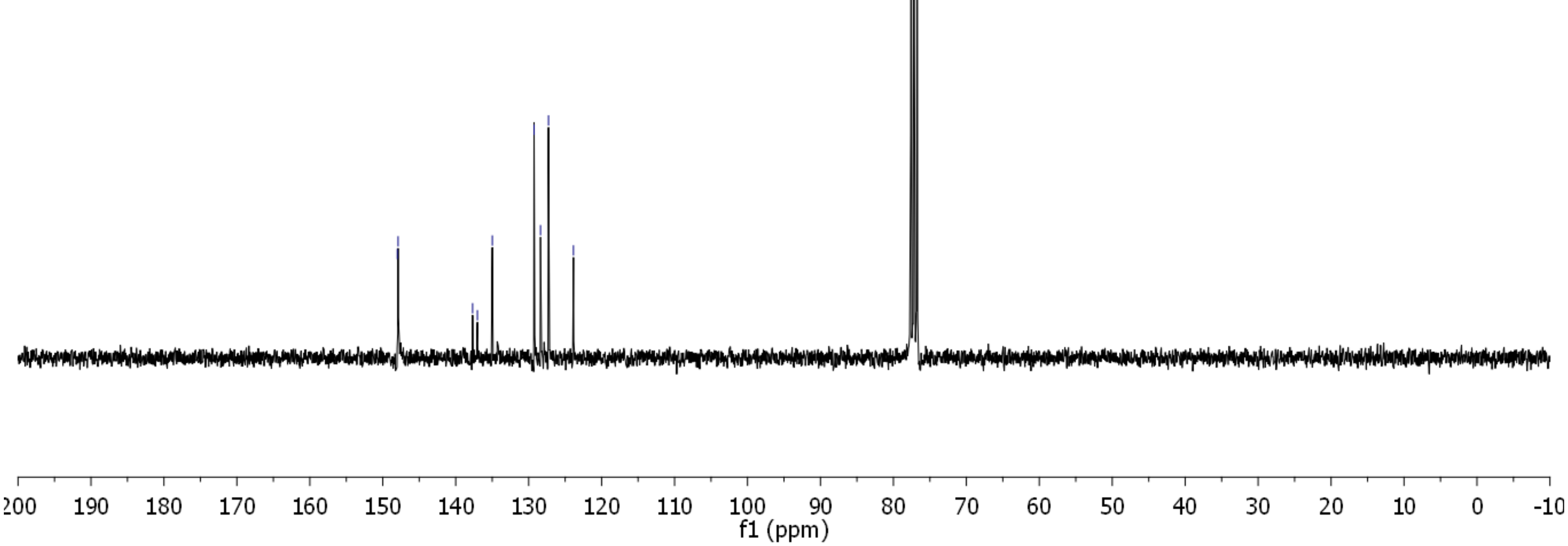

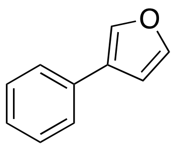

9q

^{1}H NMR (300 MHz, $CDCl_3$)

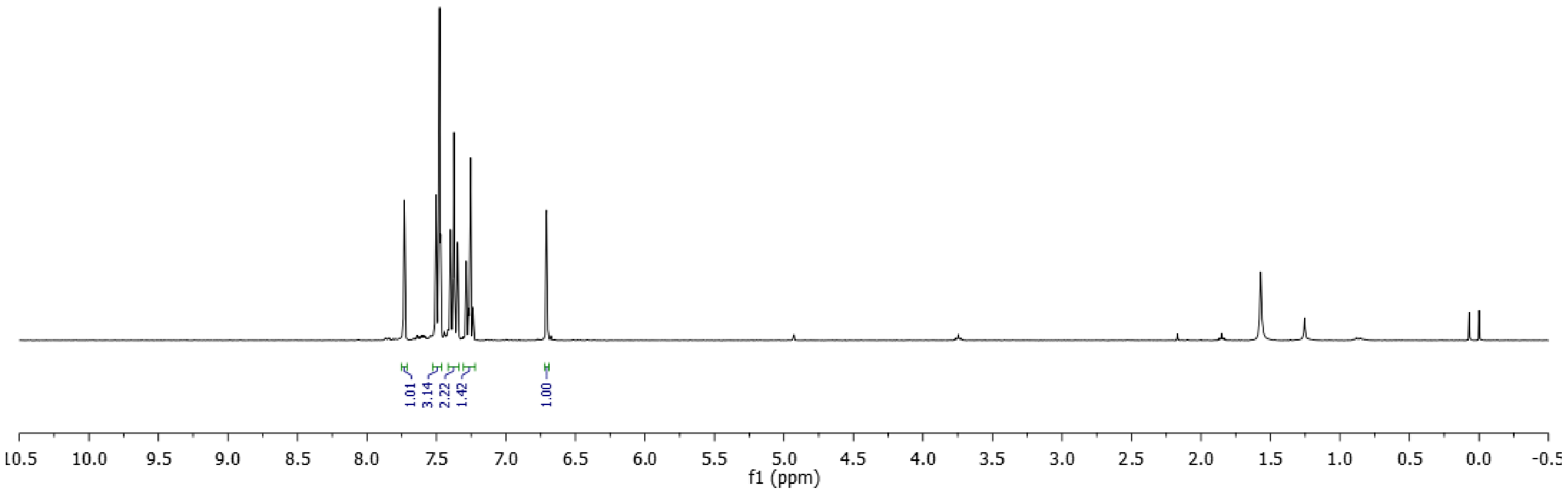

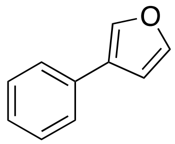

9q

^{13}C NMR (75 MHz, $CDCl_3$)

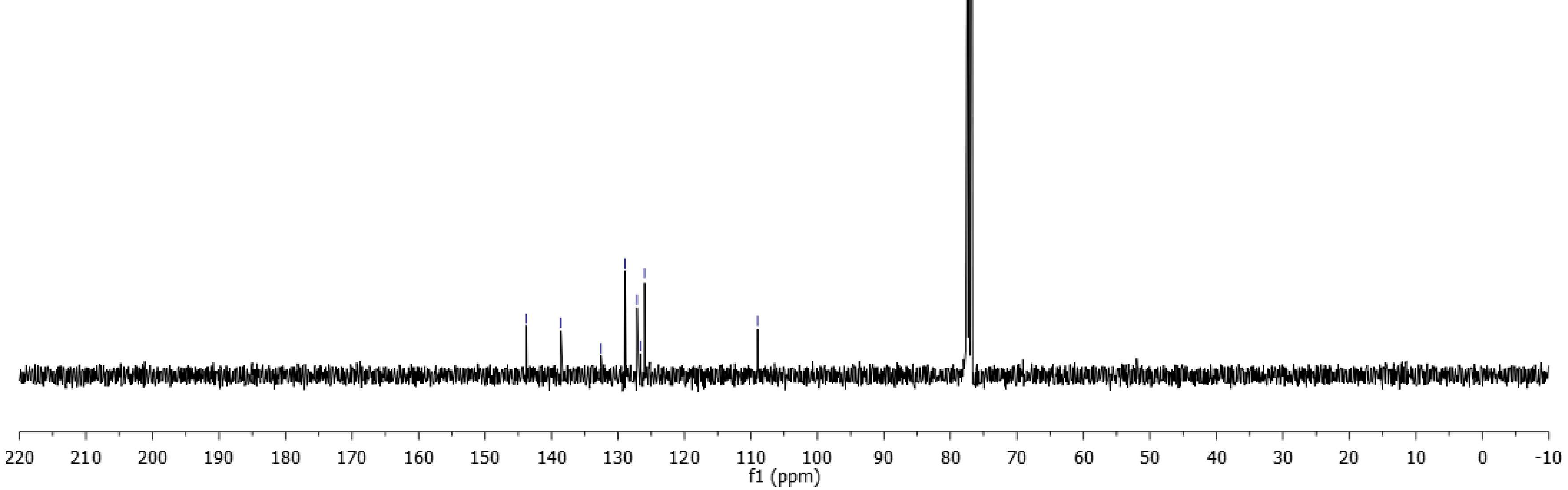

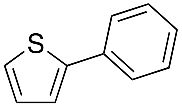

9r

^{1}H NMR (300 MHz, $CDCl_3$)

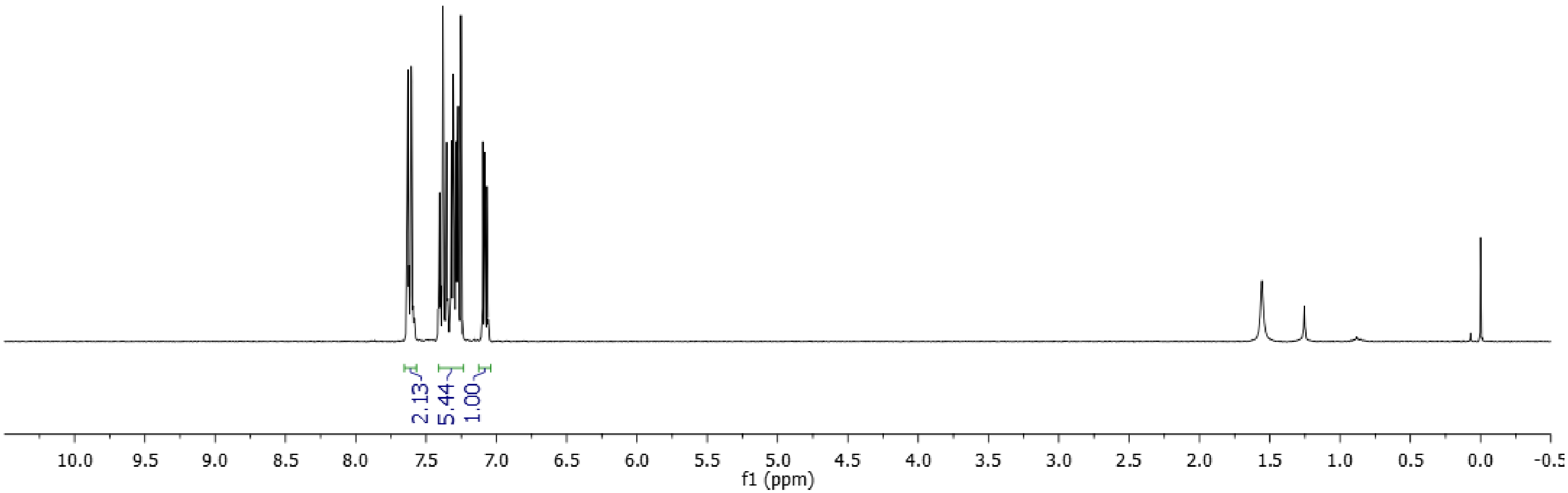

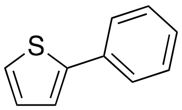

9r

^{13}C NMR (101 MHz, $CDCl_3$)

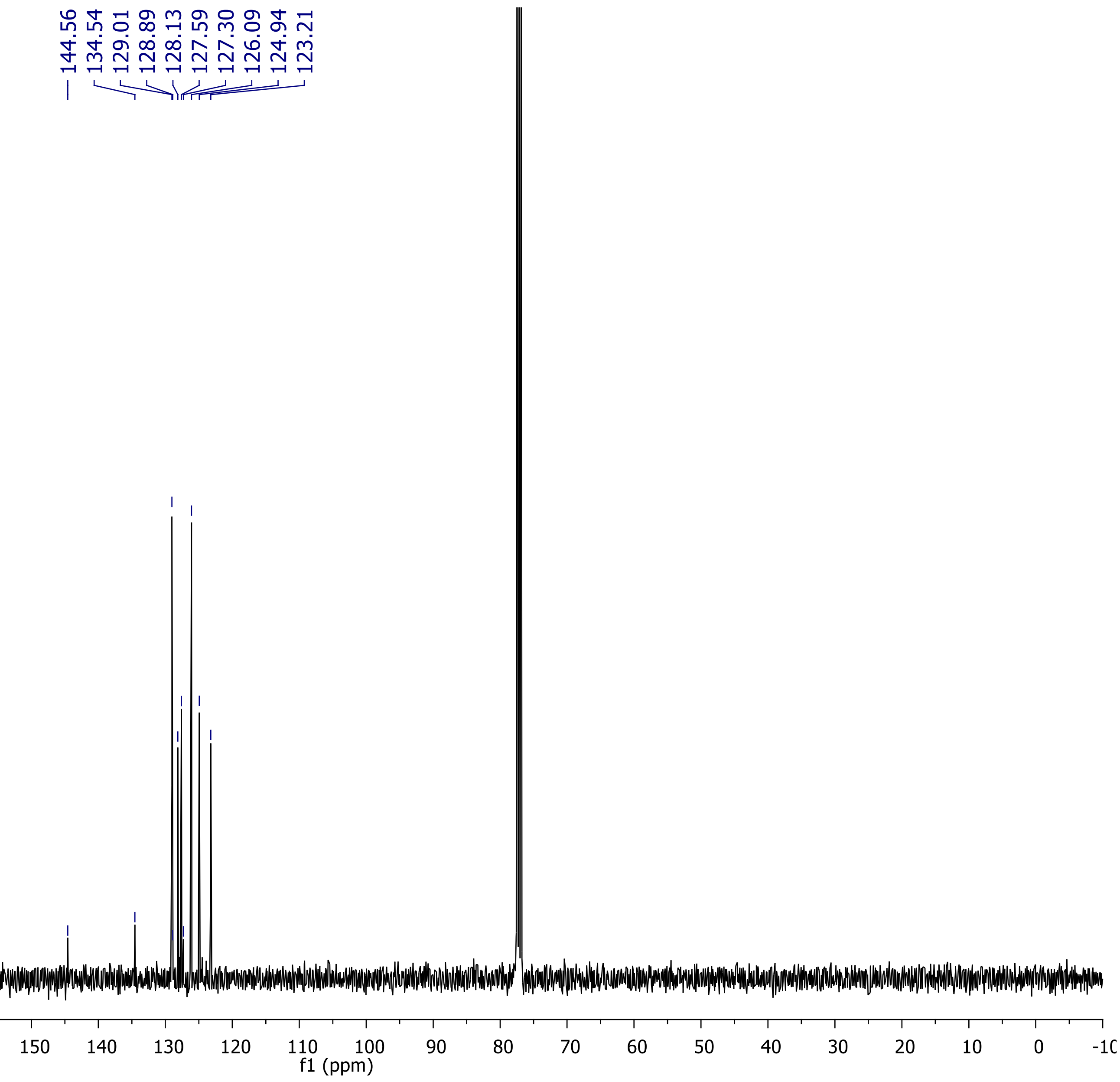

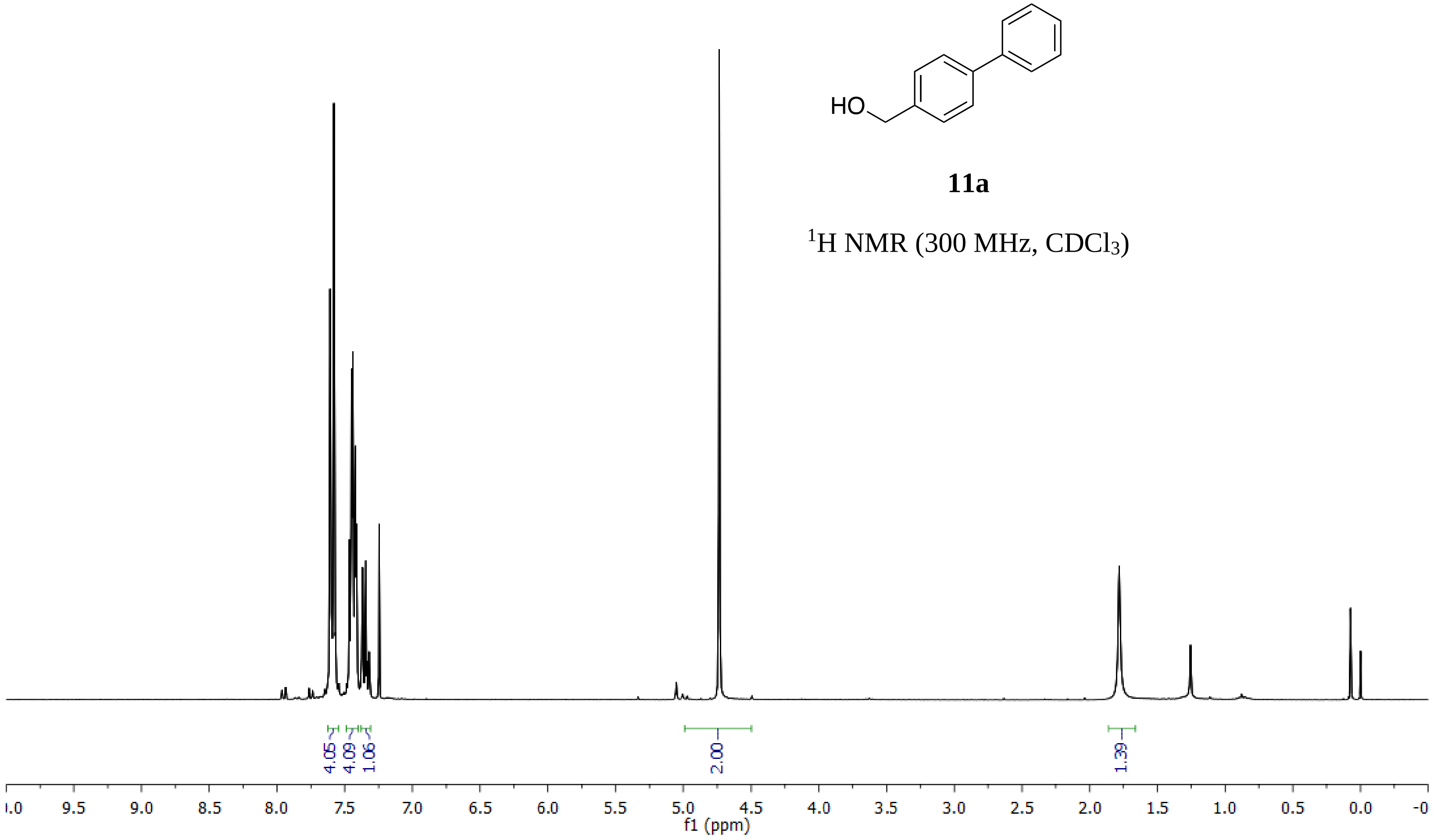
HO
11a
^{1}H NMR (300 MHz, $CDCl_3$)
4.05
4.09
1.06
2.00
1.39
9.5 9.0 8.5 8.0 7.5 7.0 6.5 6.0 5.5 5.0 4.5 4.0 3.5 3.0 2.5 2.0 1.5 1.0 0.5 0.0
f1 (ppm)

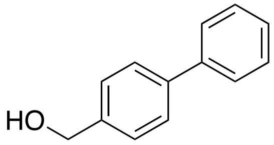

11a

^{13}C NMR (75 MHz, $CDCl_3$)

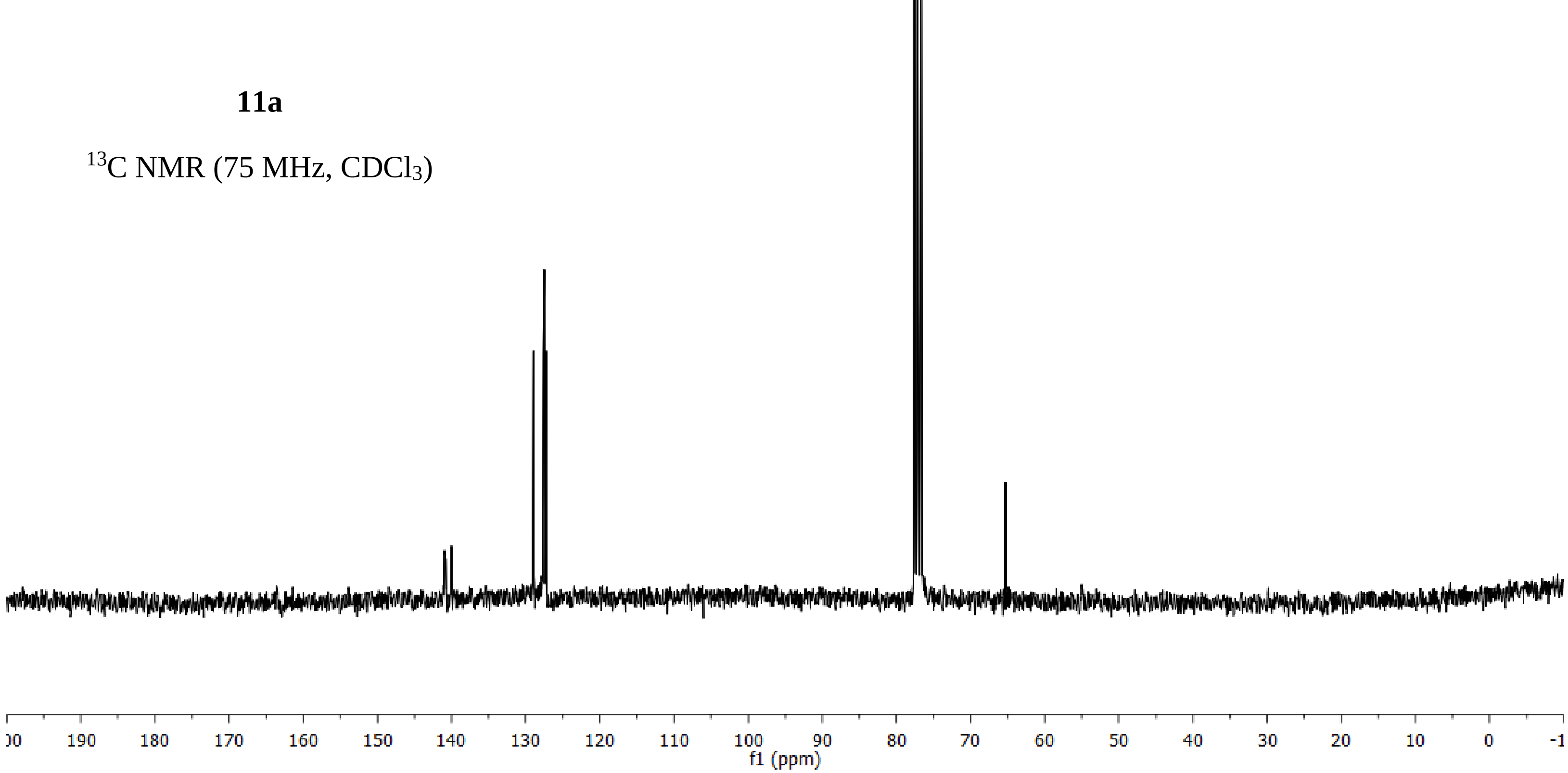

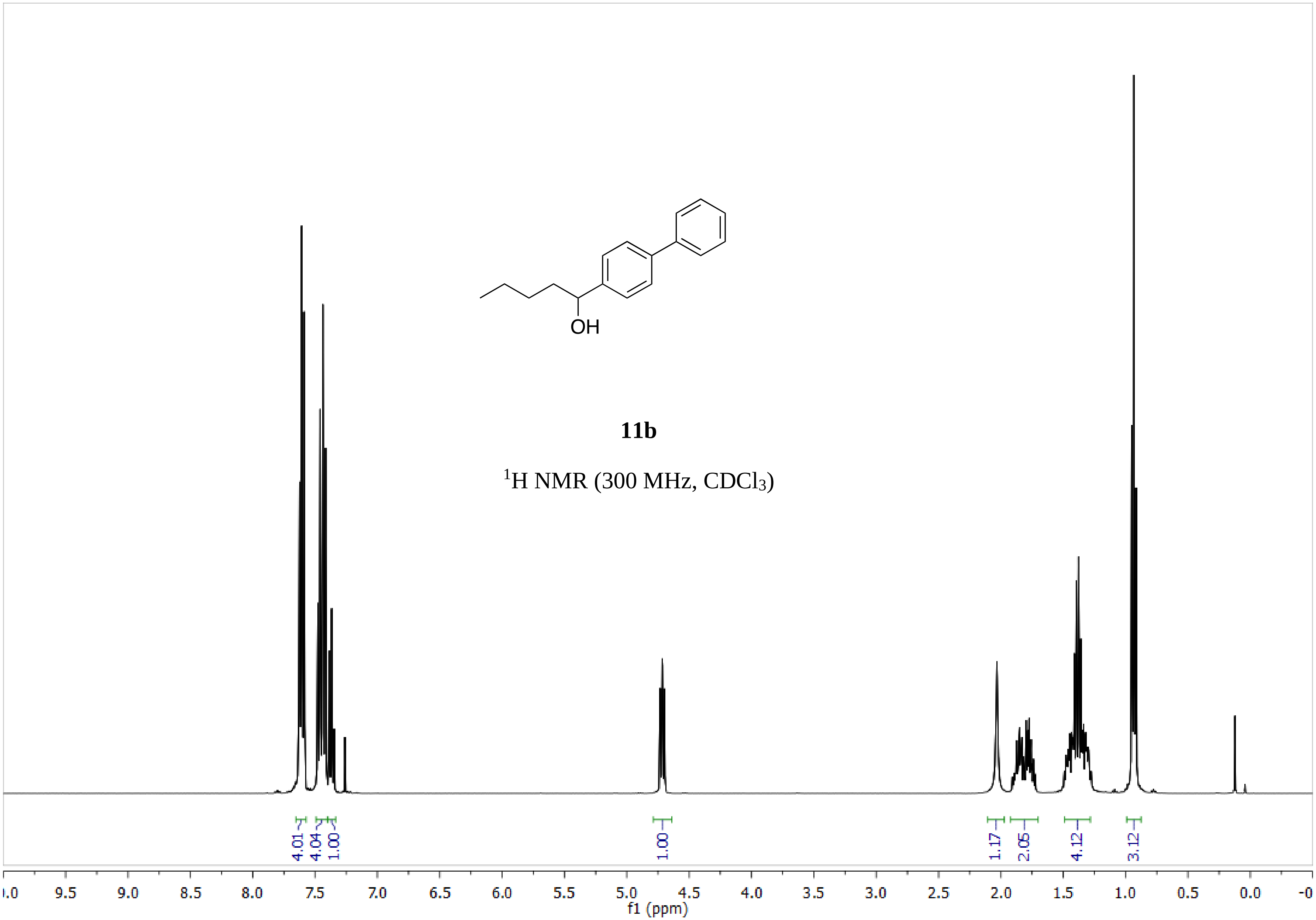

11b

^{1}H NMR (300 MHz, $CDCl_3$)

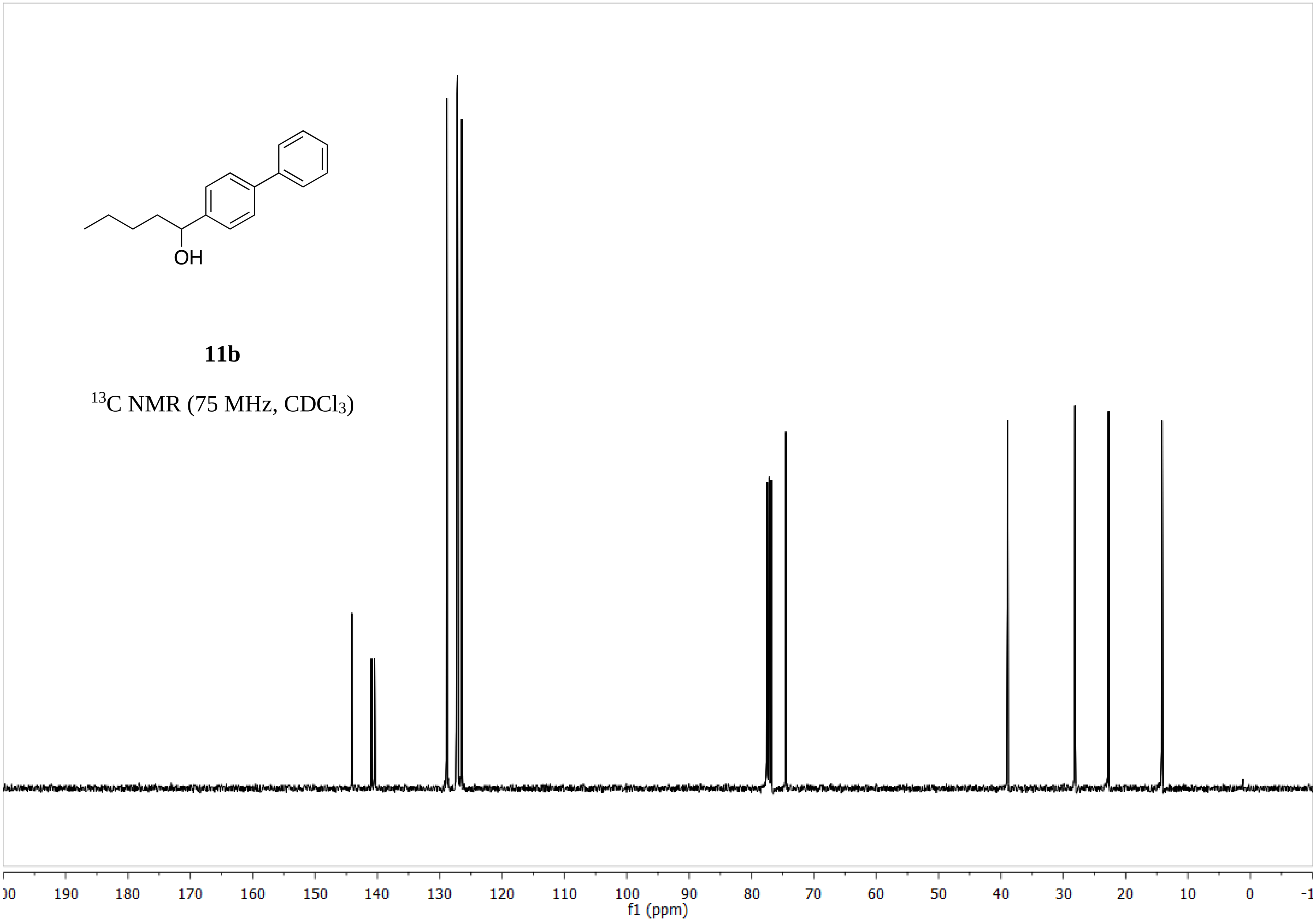

11b

^{13}C NMR (75 MHz, $CDCl_3$)

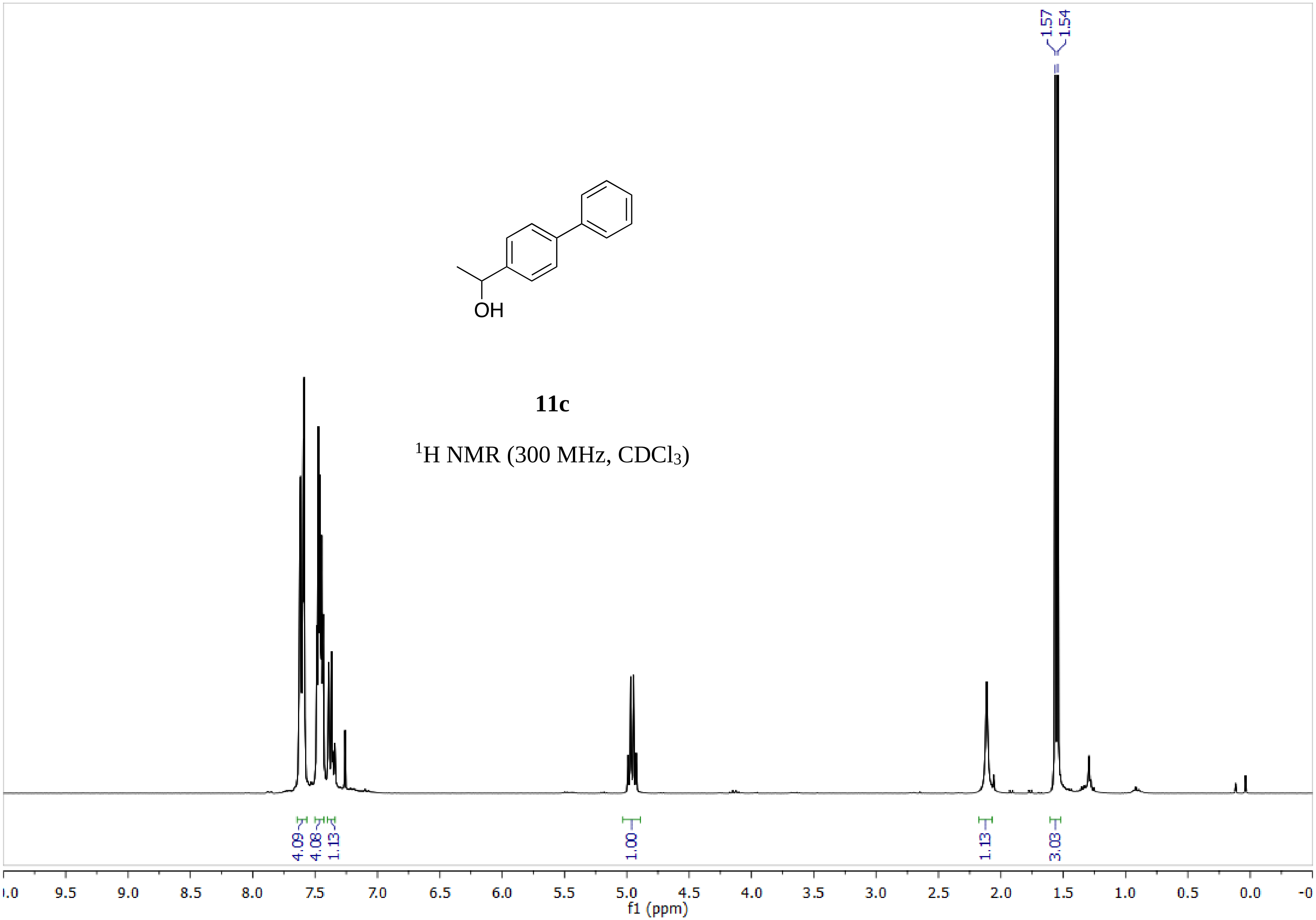
1.57
1.54
OH
11c
1H NMR (300 MHz, CDCl3)
4.09
4.08
1.13
1.00
1.13
3.03
f1 (ppm)

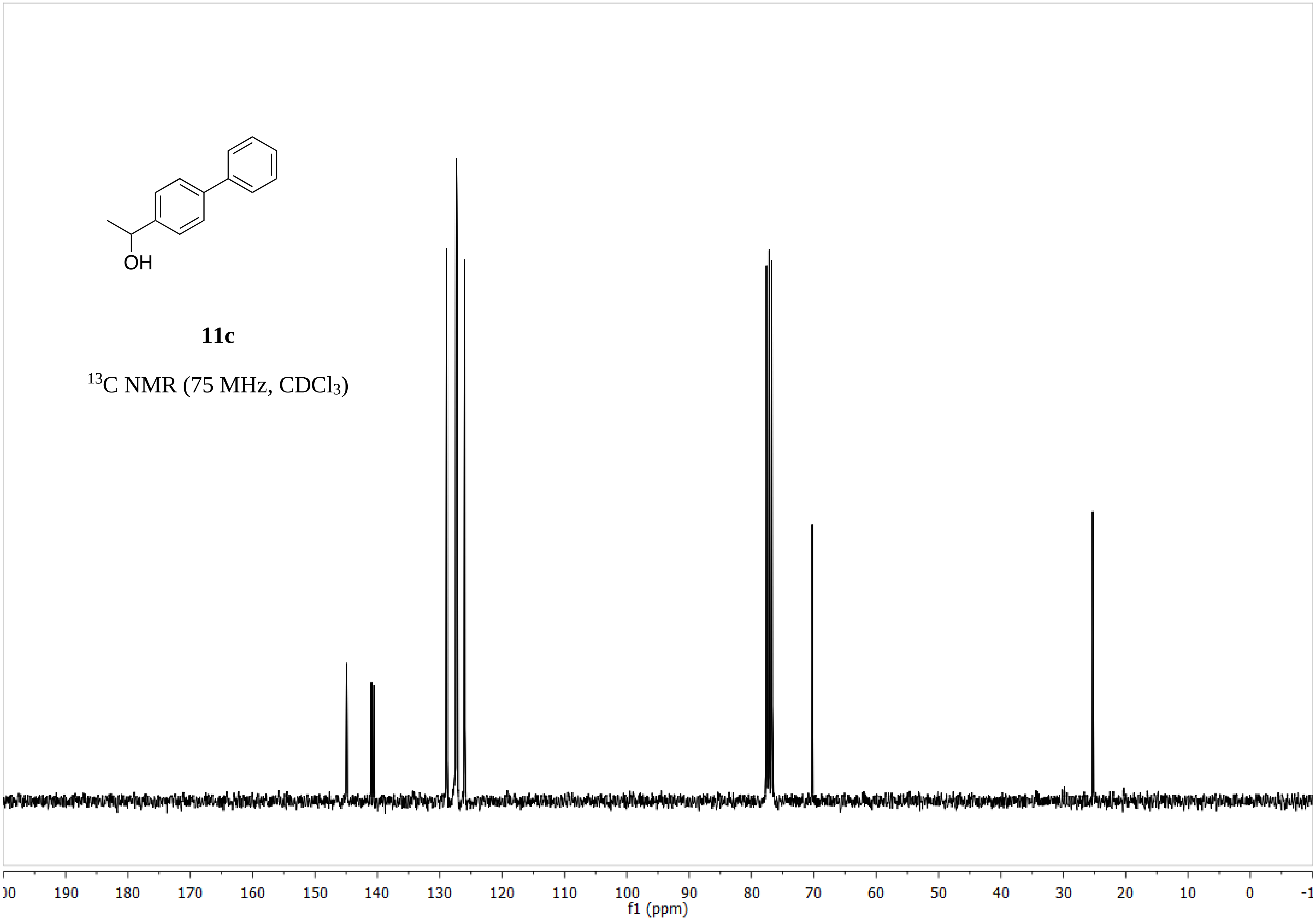
OH
11c
^{13}C NMR (75 MHz, $CDCl_3$)
f1 (ppm)

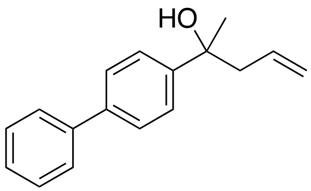

11d

^{1}H NMR (400 MHz, $CDCl_3$)

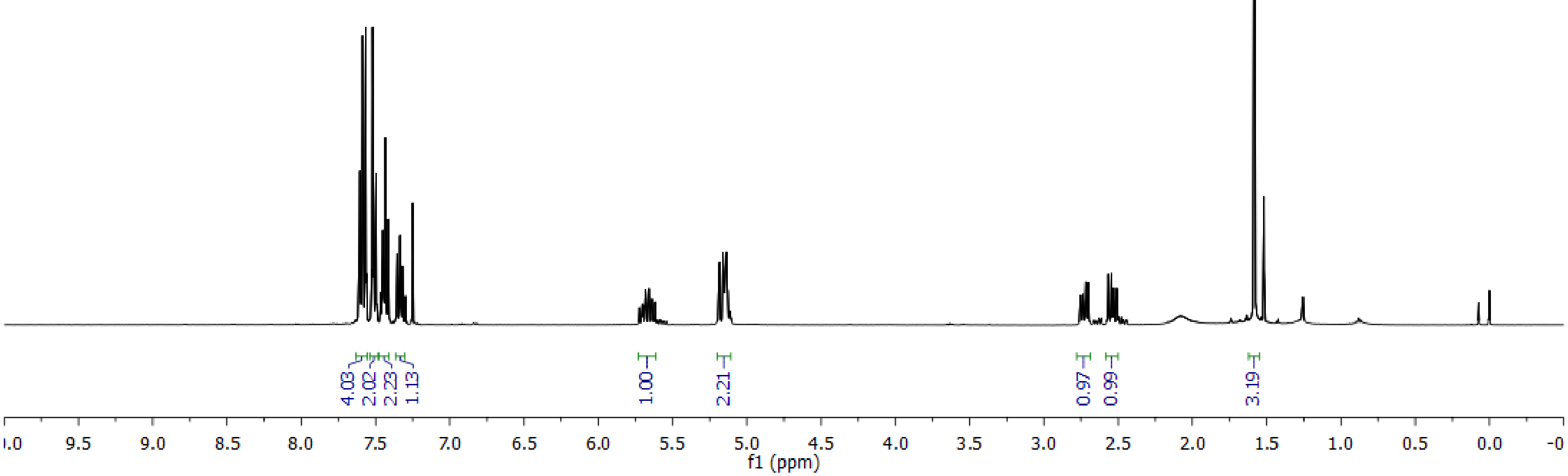

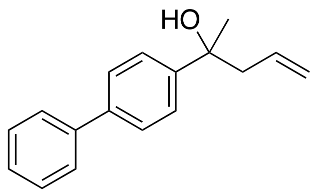

11d

^{13}C NMR (101 MHz, $CDCl_3$)

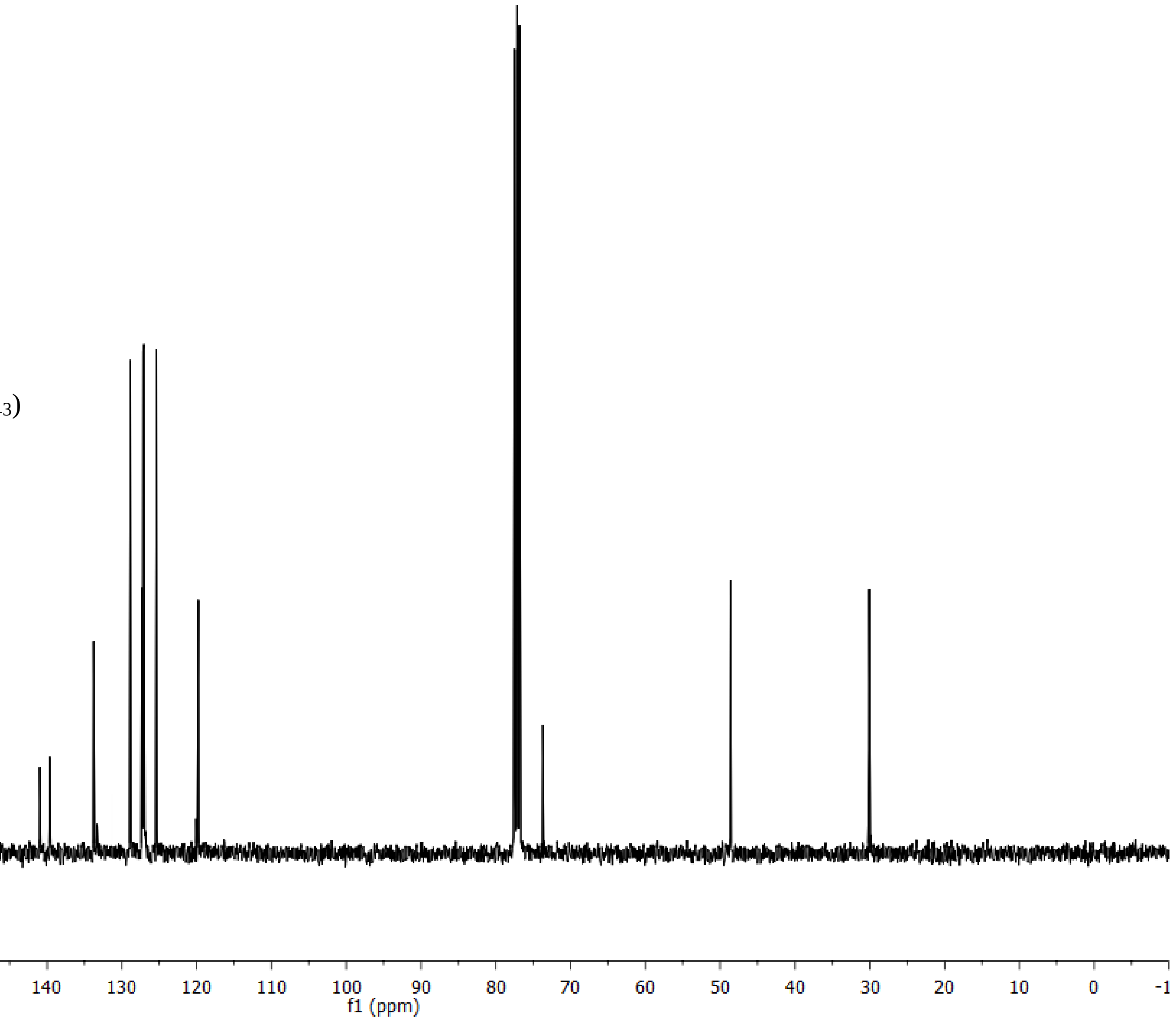

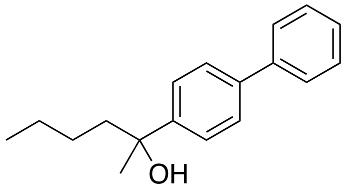

11e

^{1}H NMR (400 MHz, $CDCl_3$)

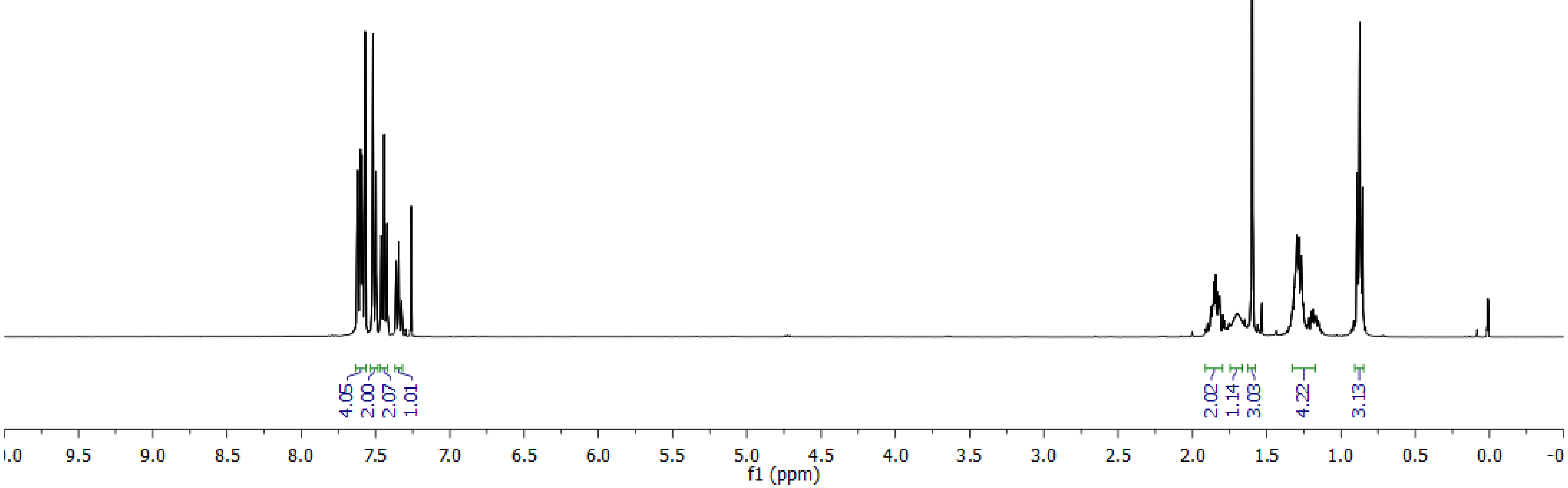

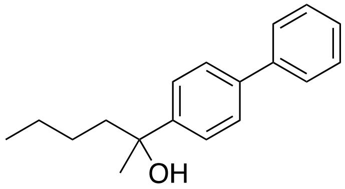

11e

^{13}C NMR (101 MHz, $CDCl_3$)

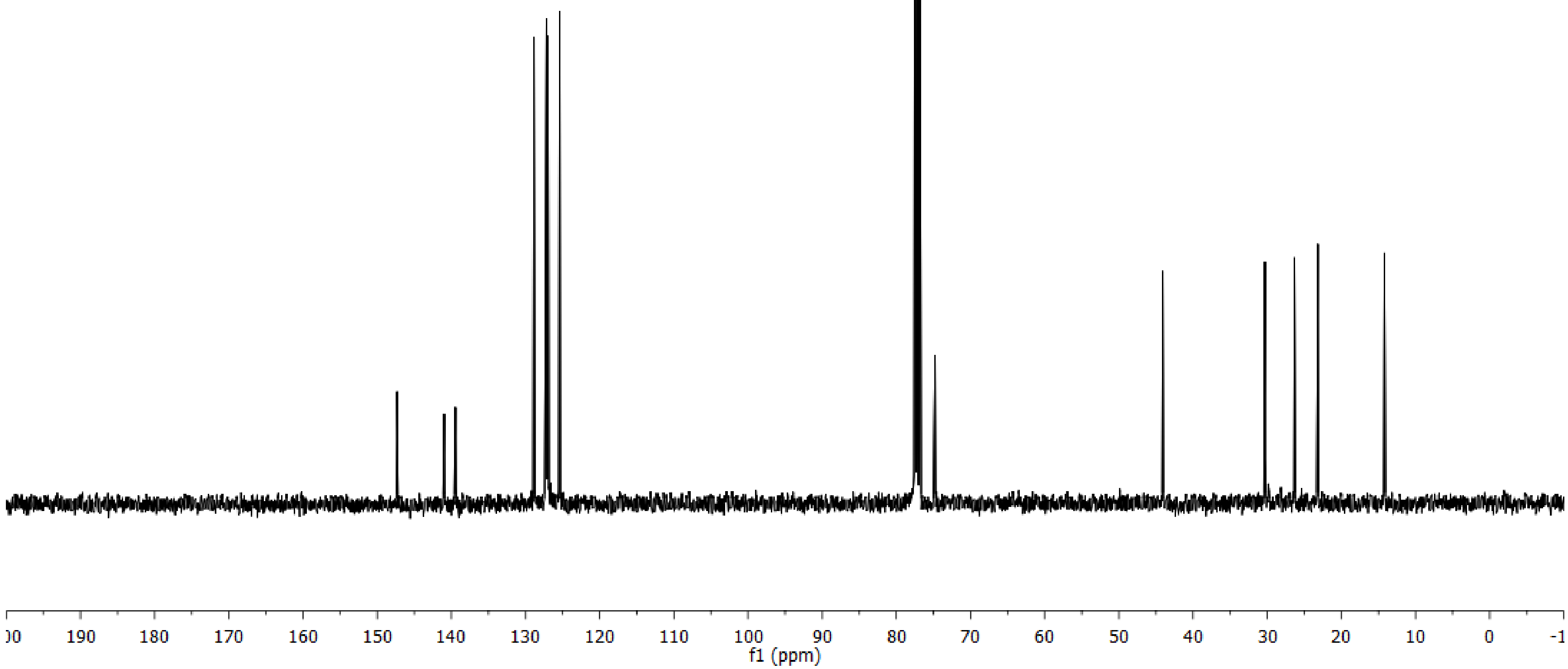

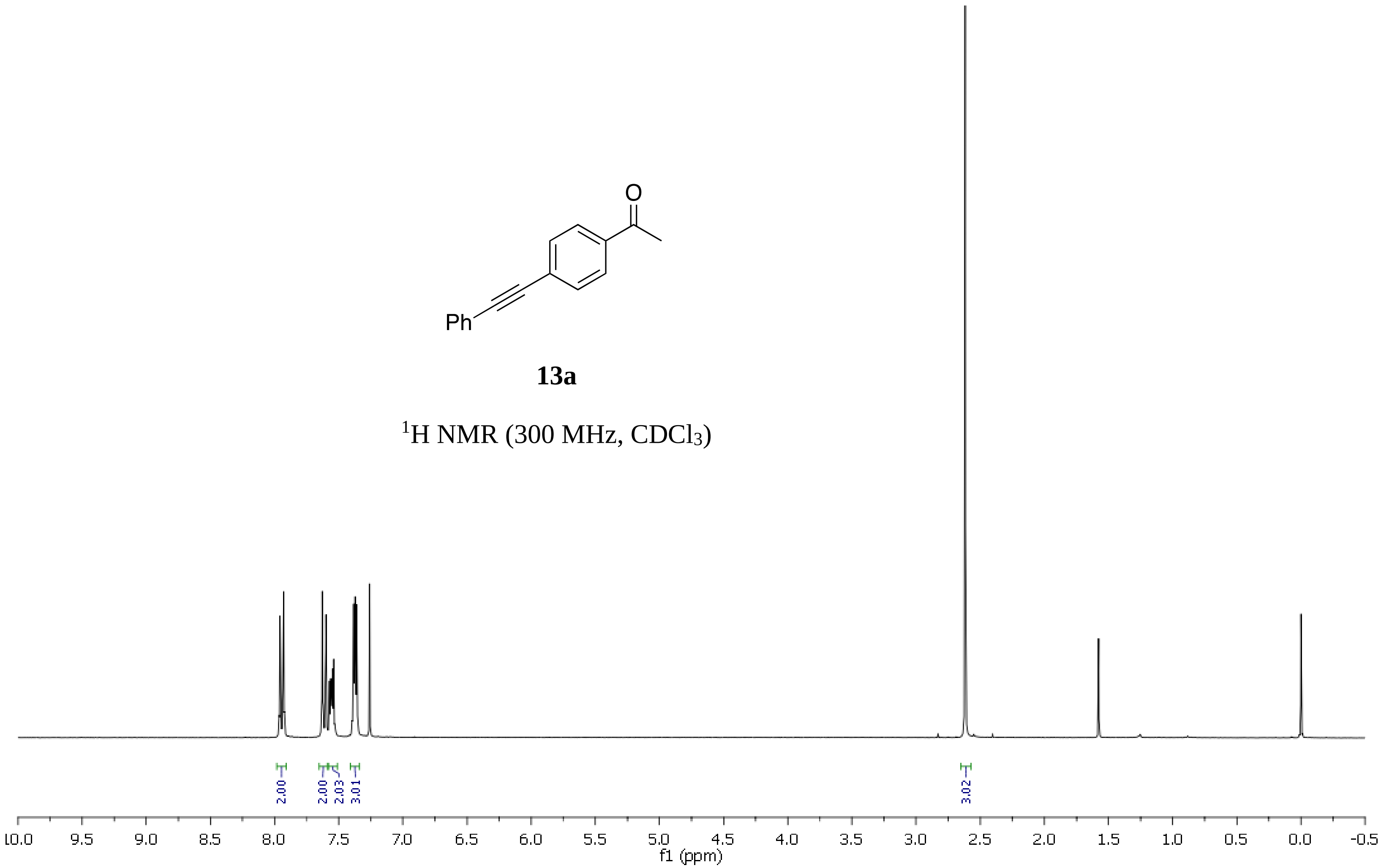

13a

^{1}H NMR (300 MHz, $CDCl_3$)

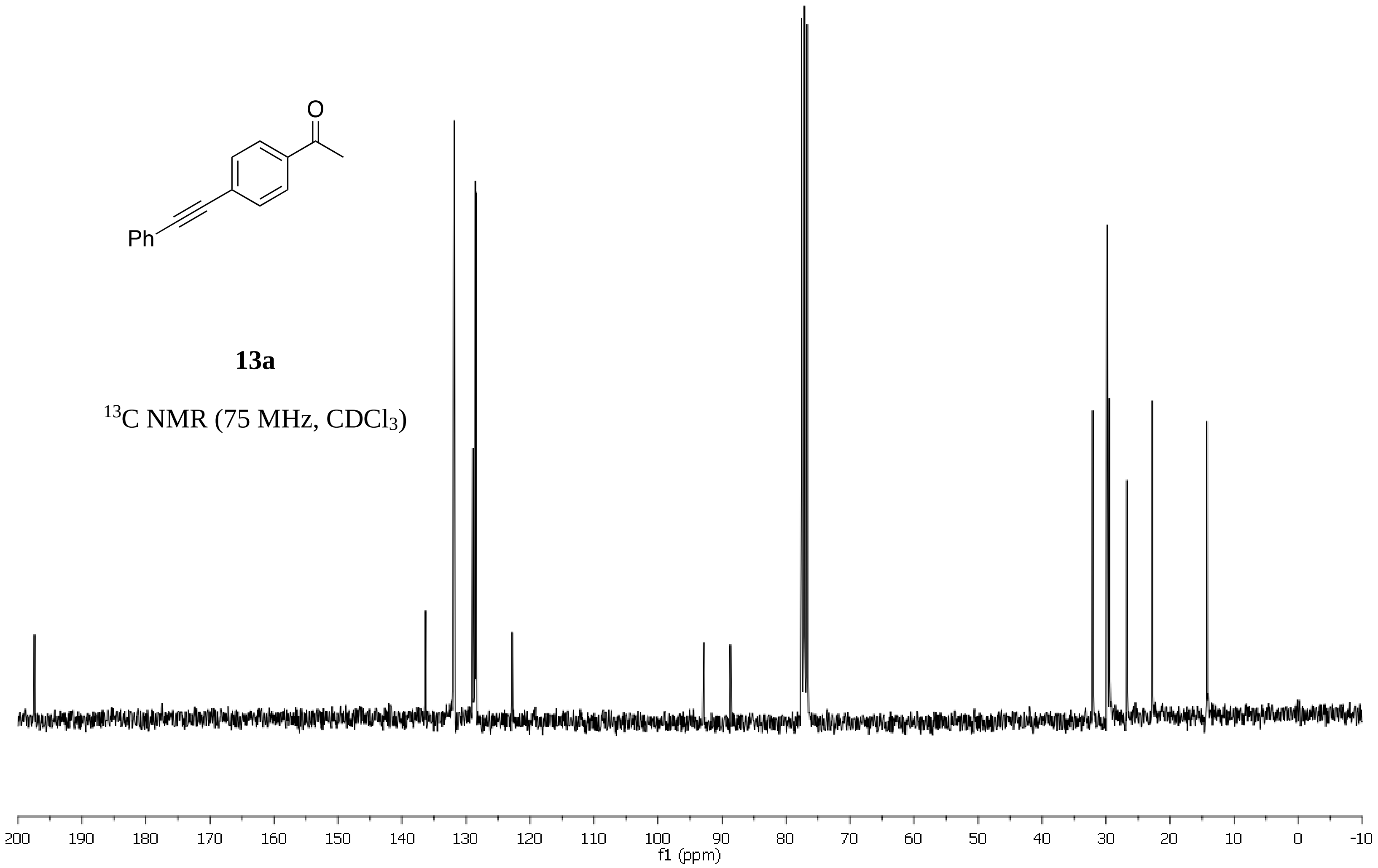

13a

^{13}C NMR (75 MHz, $CDCl_3$)

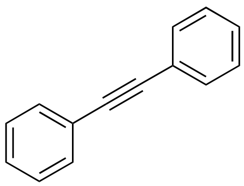

13b

^{1}H NMR (300 MHz, $CDCl_3$)

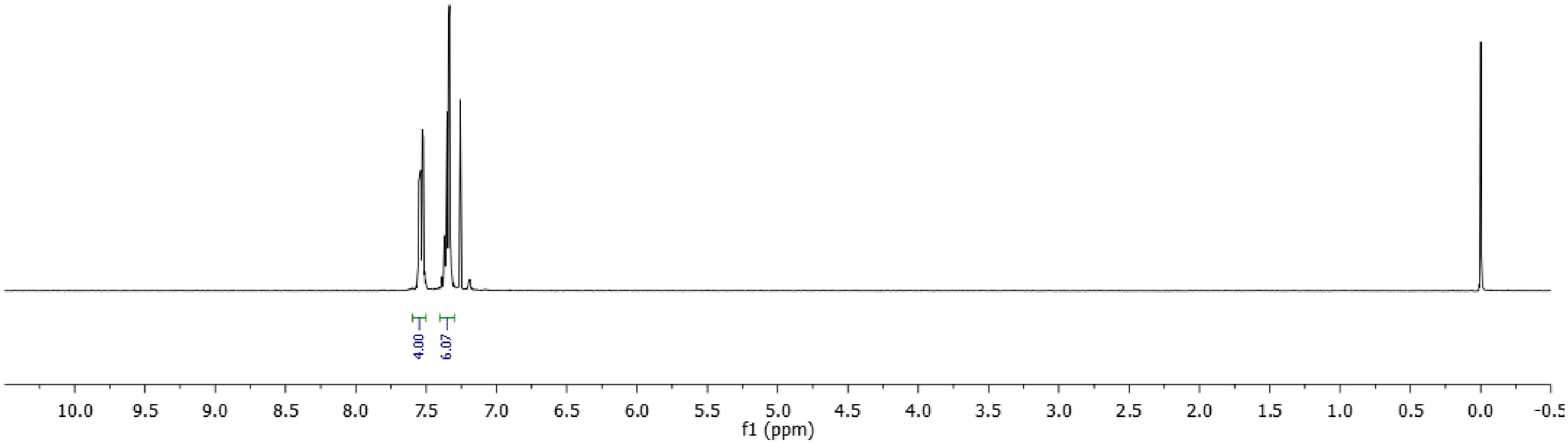

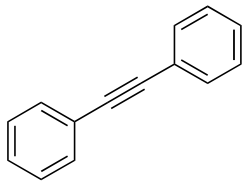

13b

^{13}C NMR (75 MHz, $CDCl_3$)

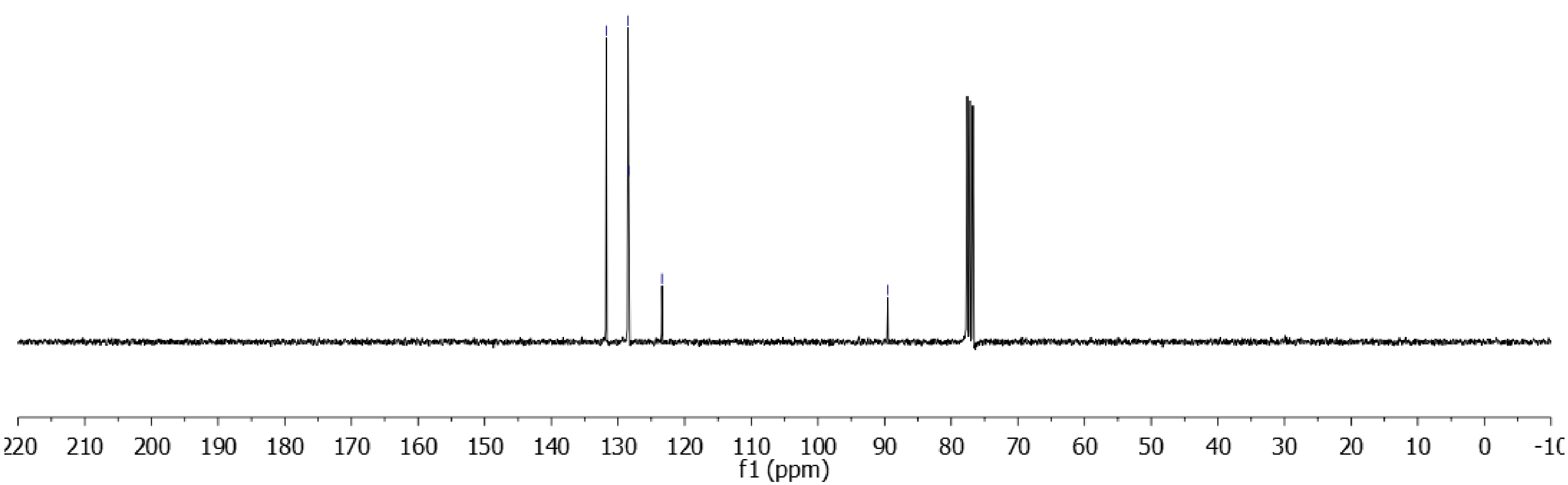

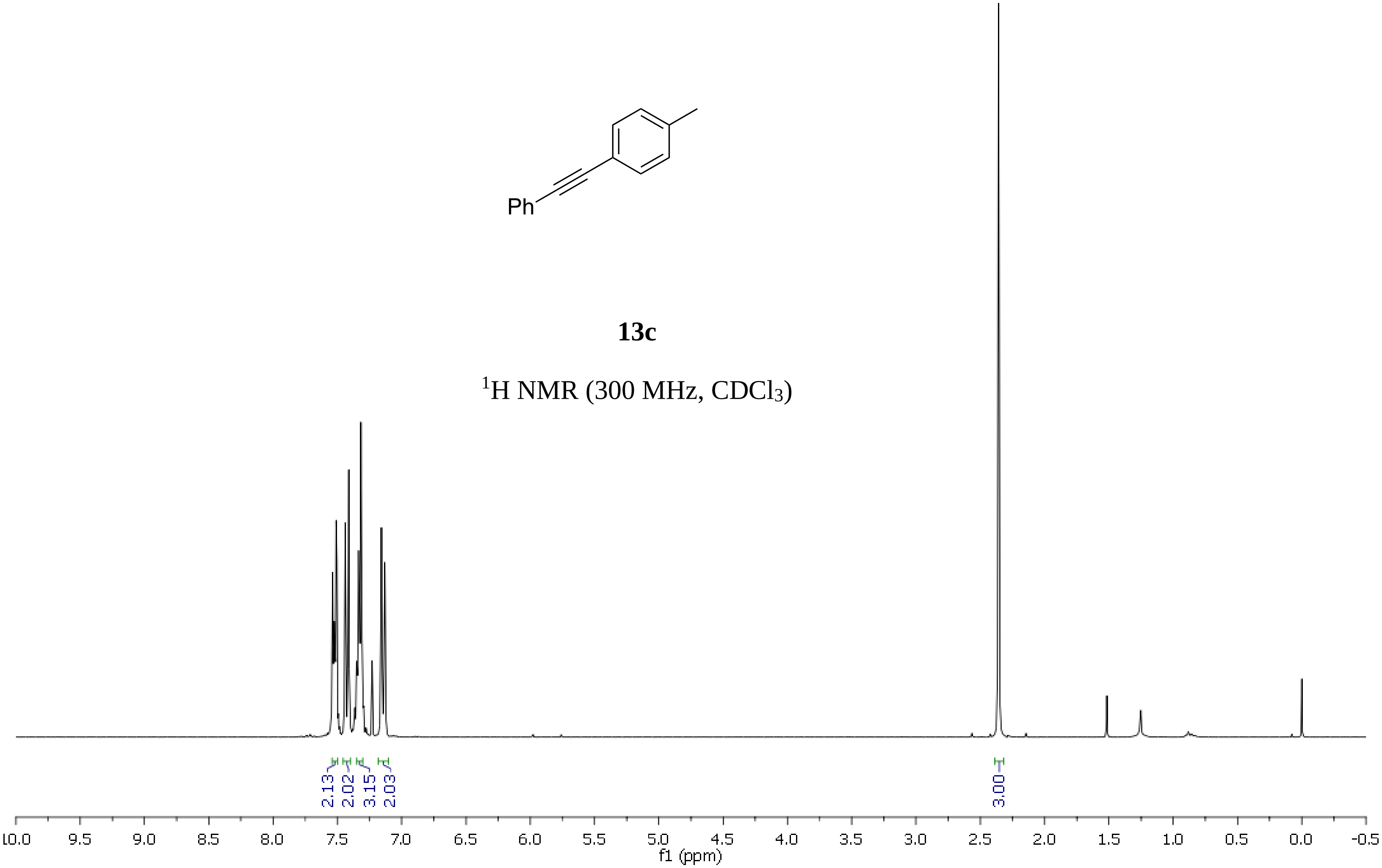

13c

^{1}H NMR (300 MHz, $CDCl_3$)

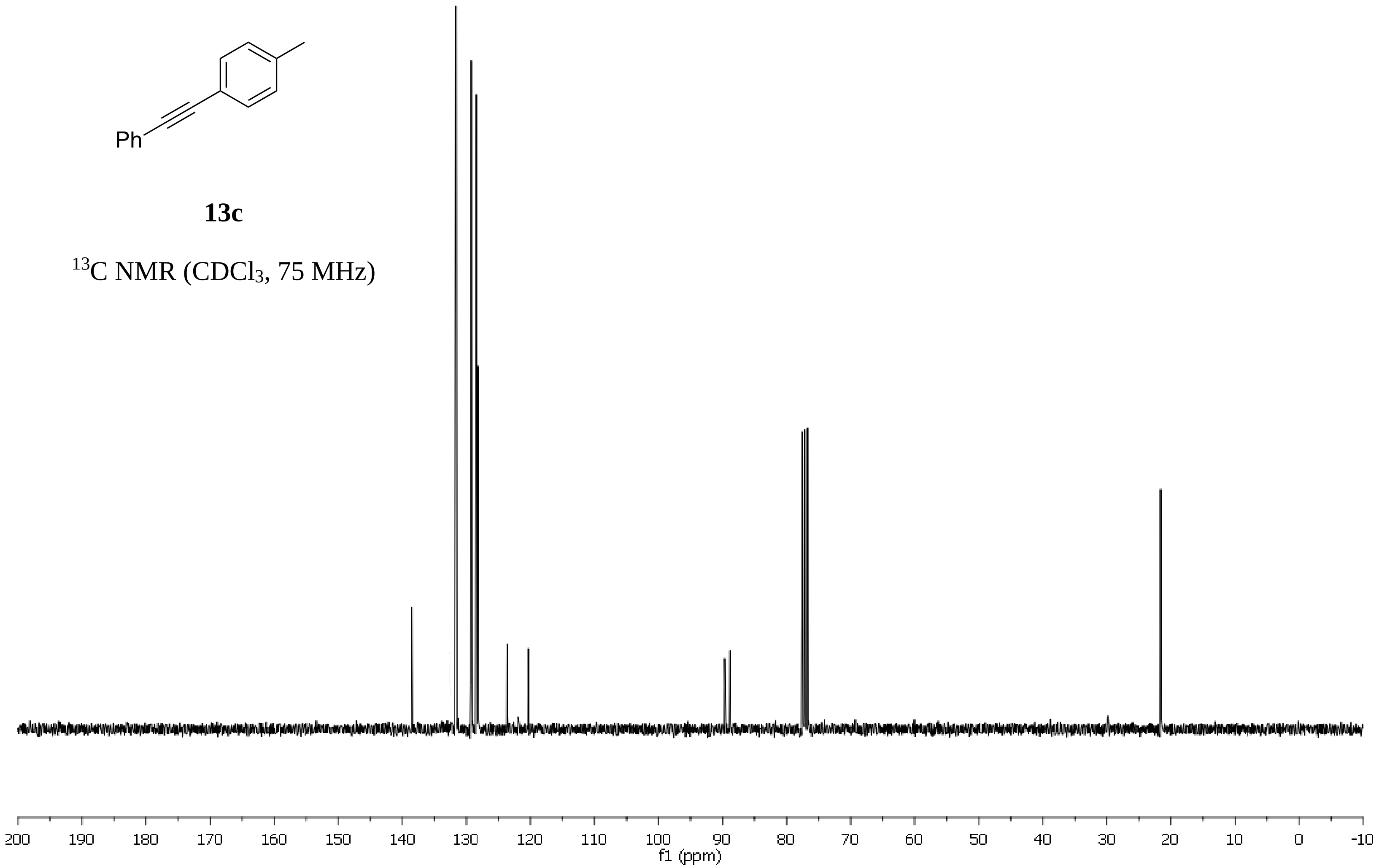

13c

^{13}C NMR ($CDCl_3$, 75 MHz)

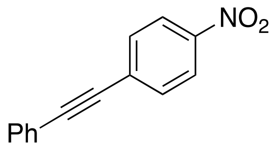

13d

^{1}H NMR (300 MHz, $CDCl_3$)

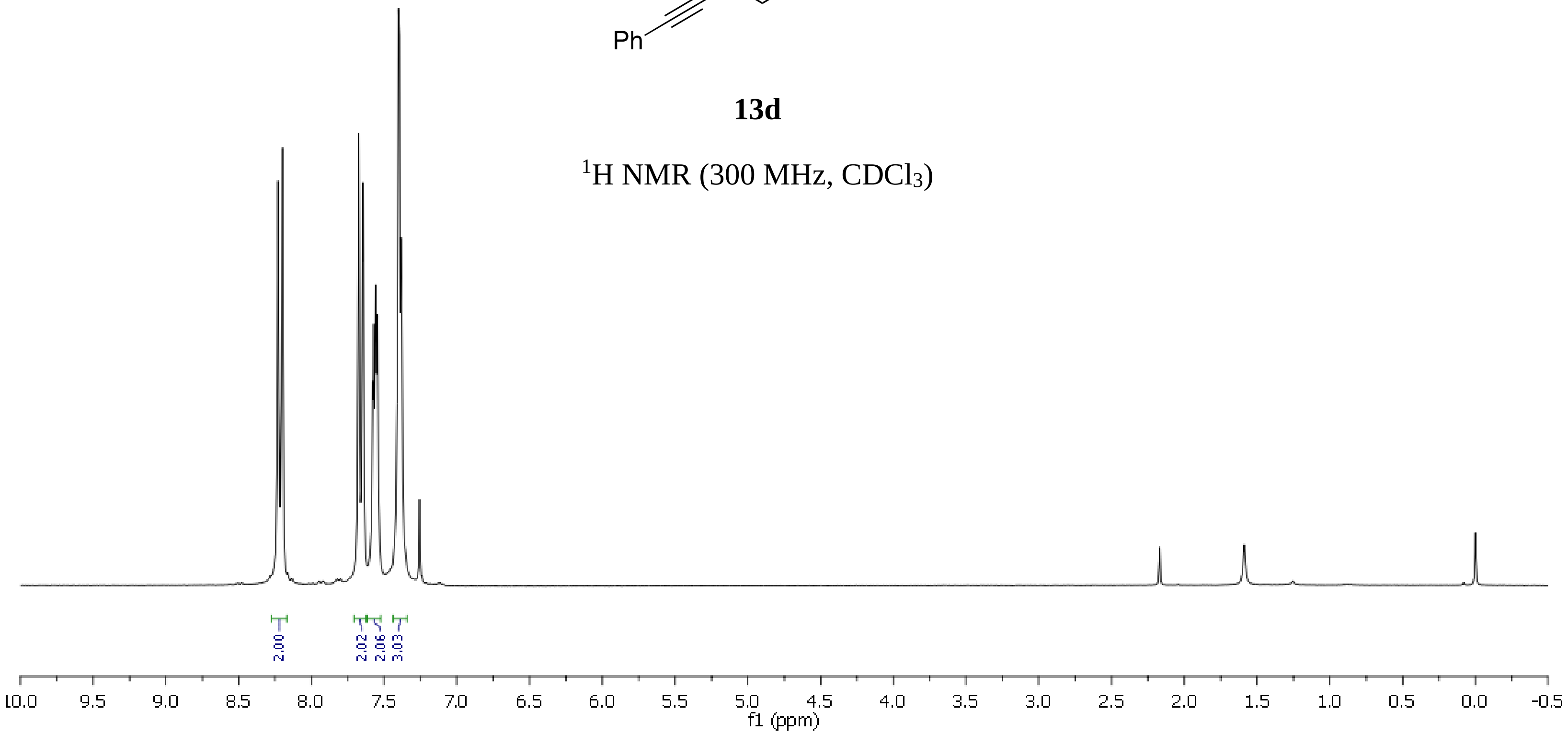

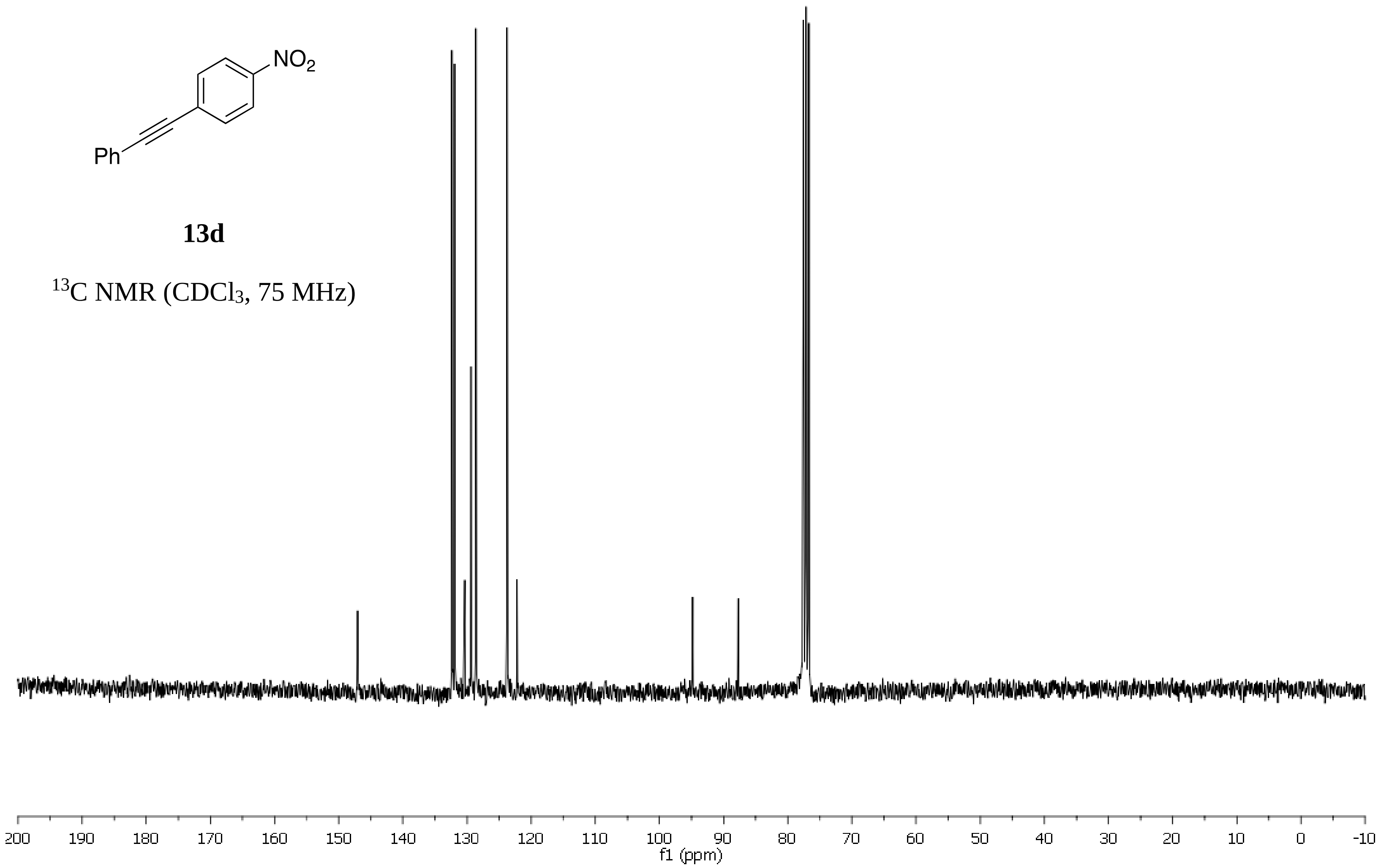

13d

^{13}C NMR ($CDCl_3$, 75 MHz)

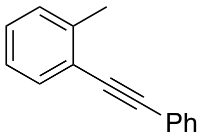

13e

^{1}H NMR ($CDCl_3$, 300 MHz)

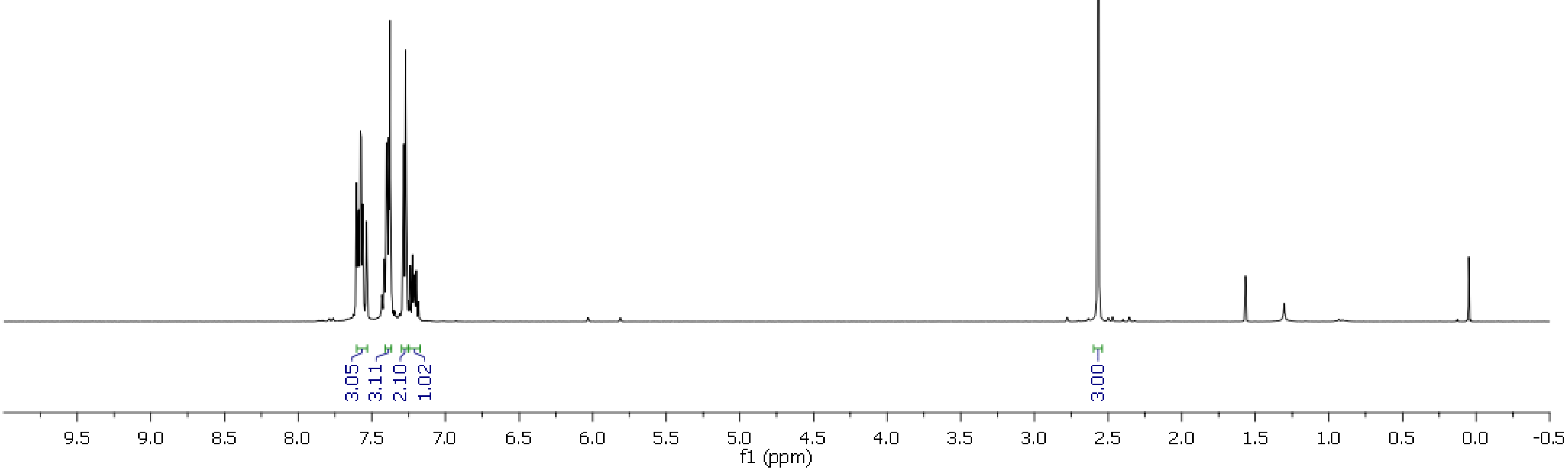

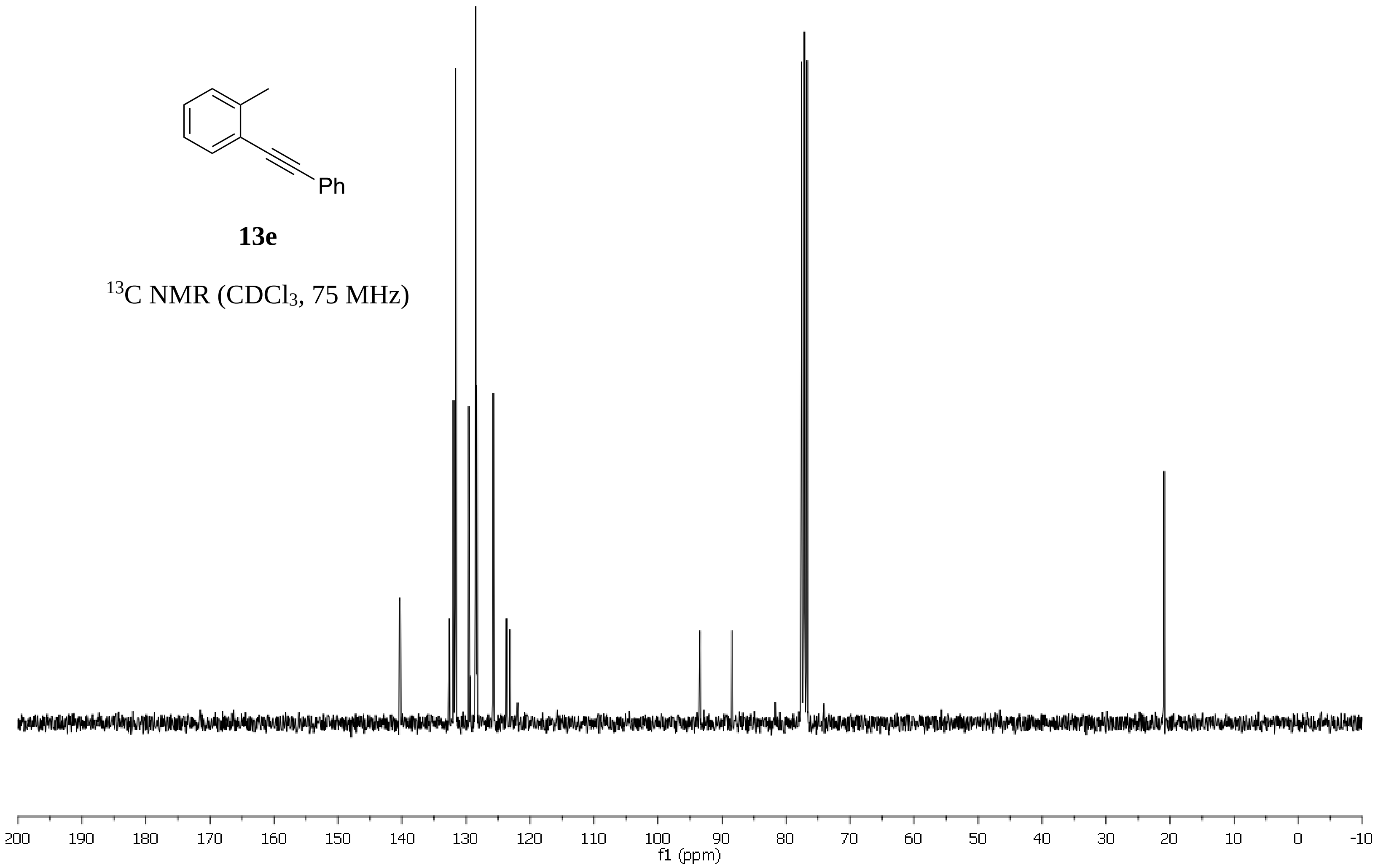

13e

^{13}C NMR ($CDCl_3$, 75 MHz)

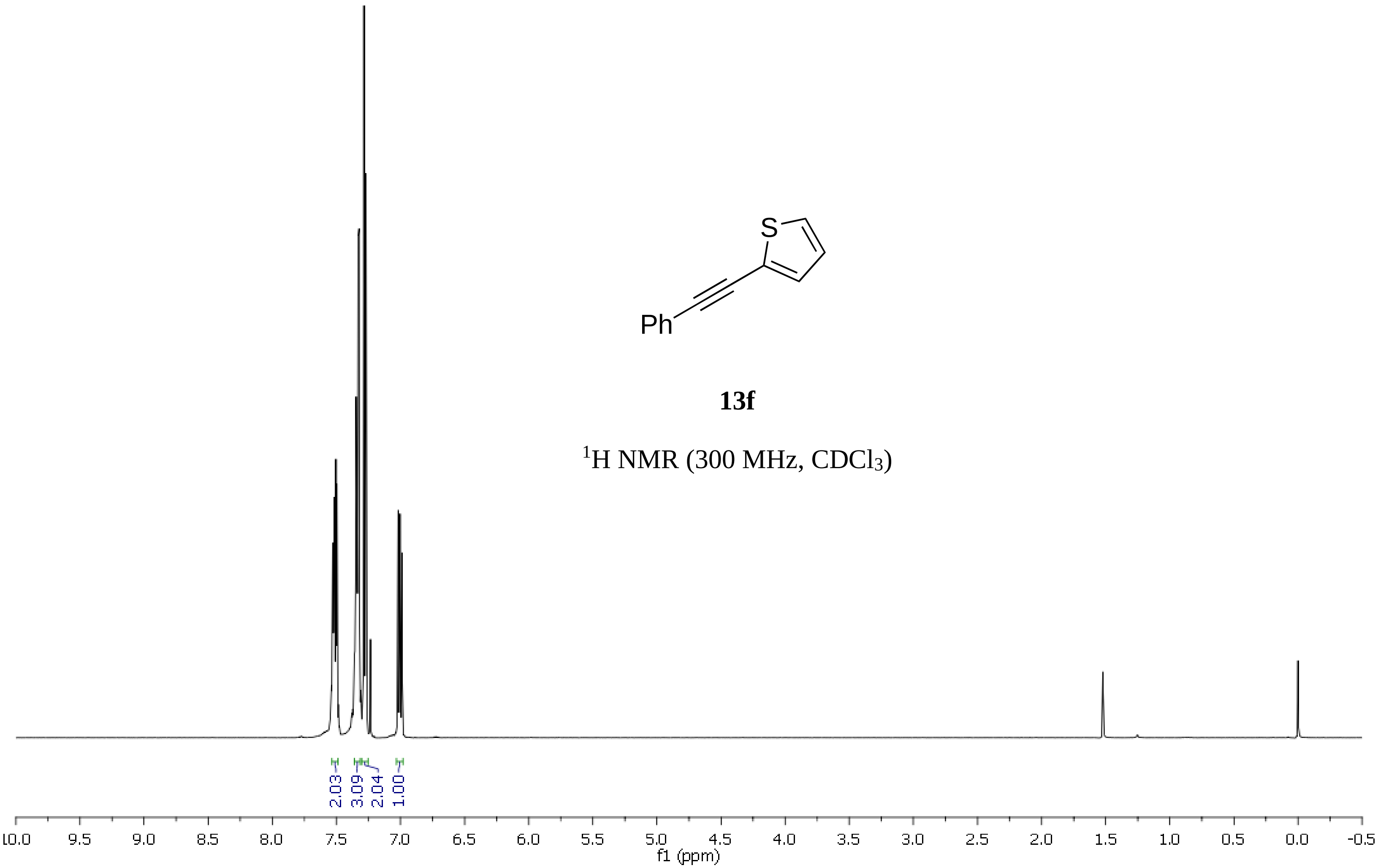

13f

^{1}H NMR (300 MHz, $CDCl_3$)

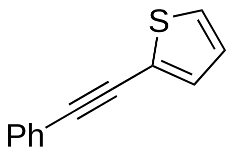

13f

^{13}C NMR ($CDCl_3$, 75 MHz)

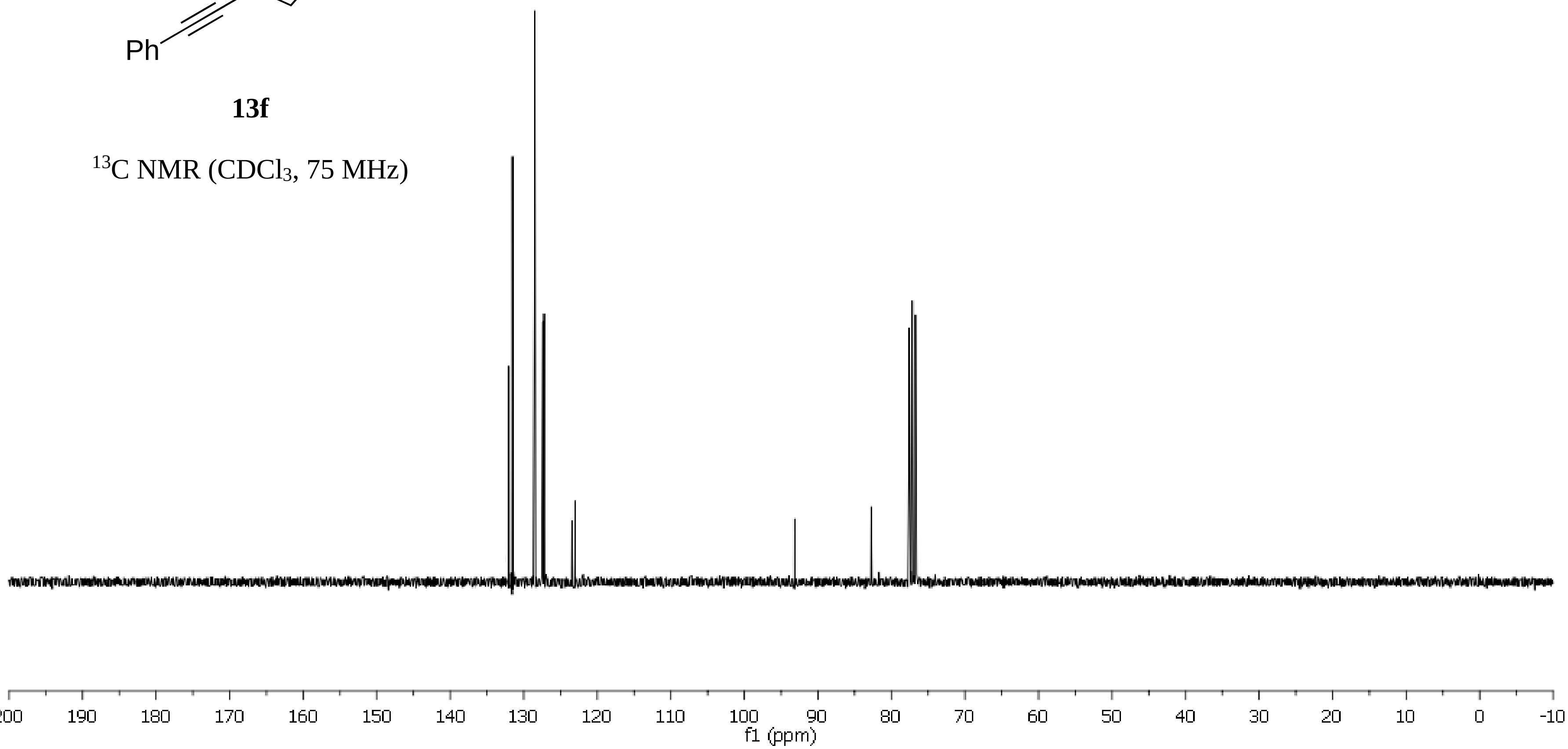

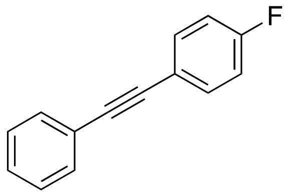

13g

^{1}H NMR (300 MHz, $CDCl_3$)

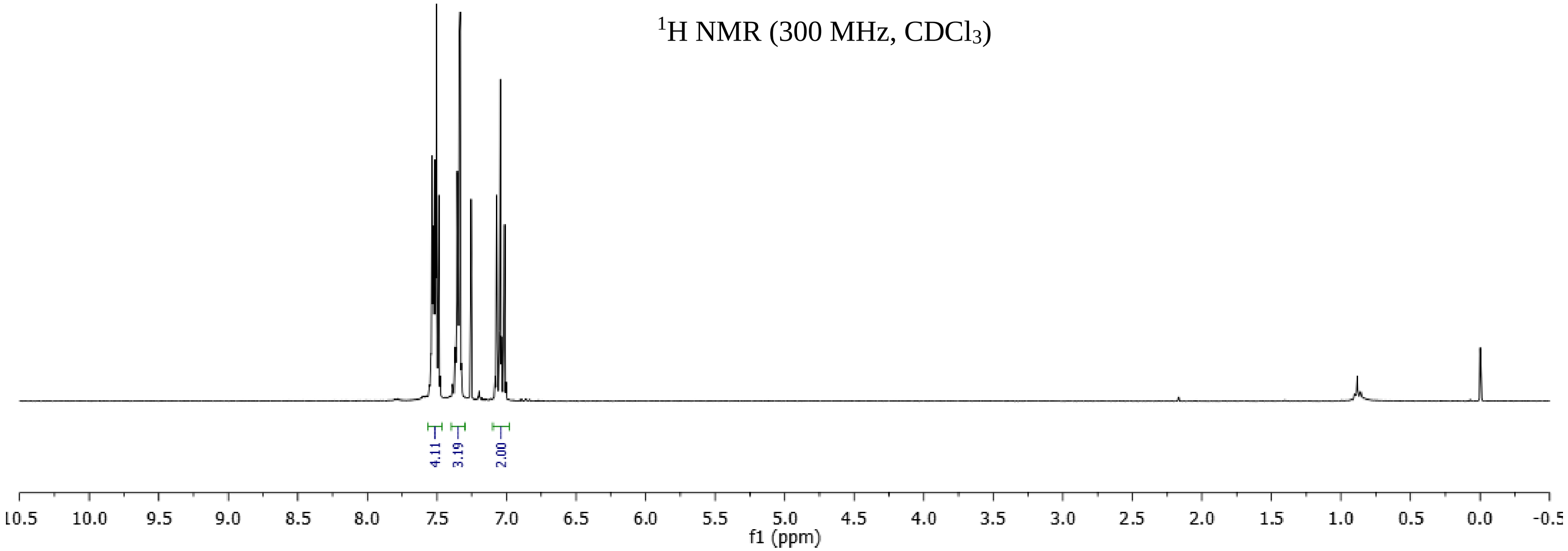

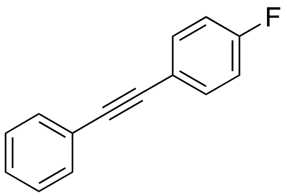

13g

^{13}C NMR ($CDCl_3$, 75 MHz)

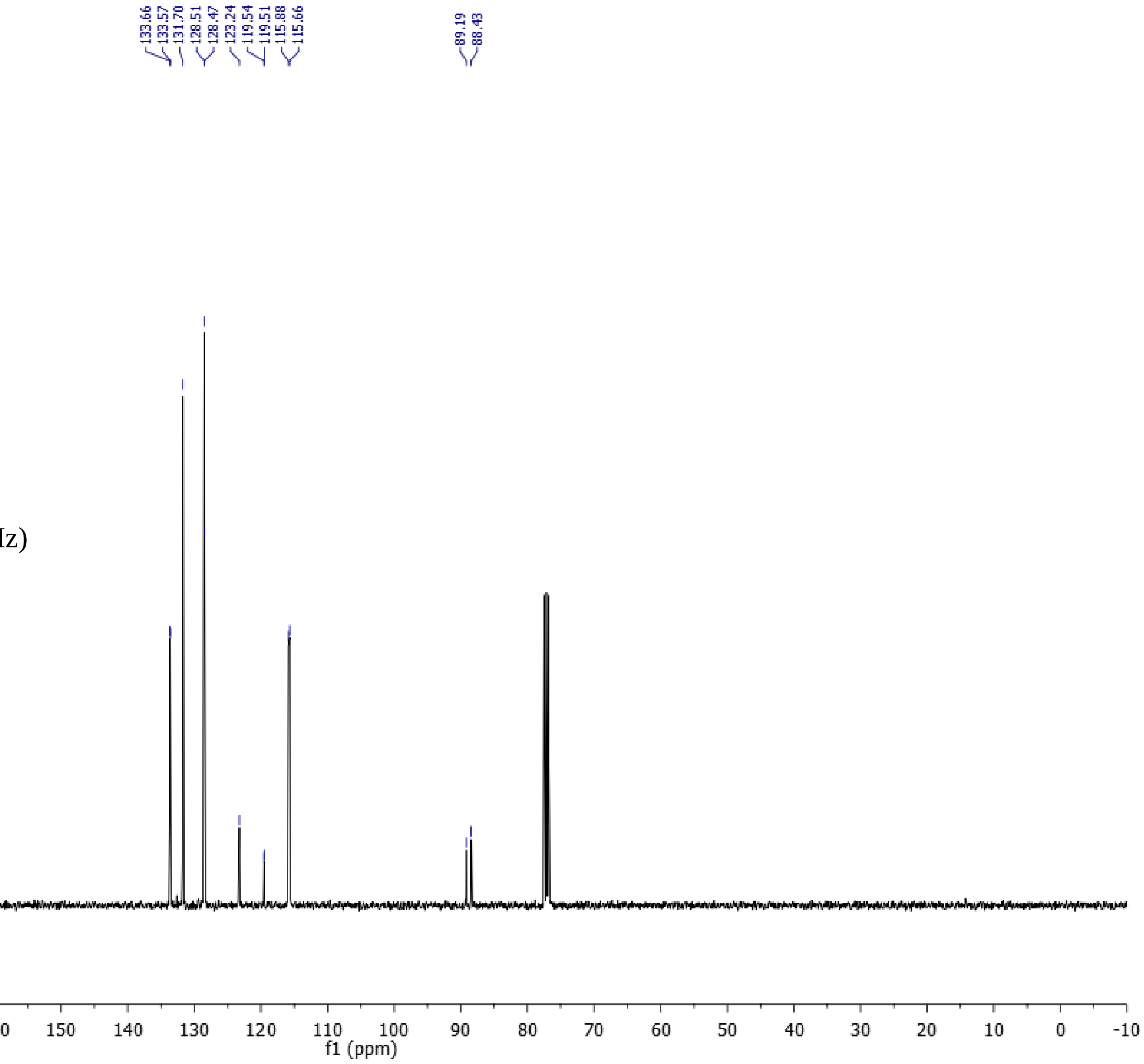

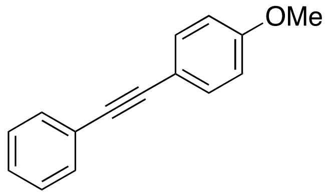

13h

^{1}H NMR (300 MHz, $CDCl_3$)

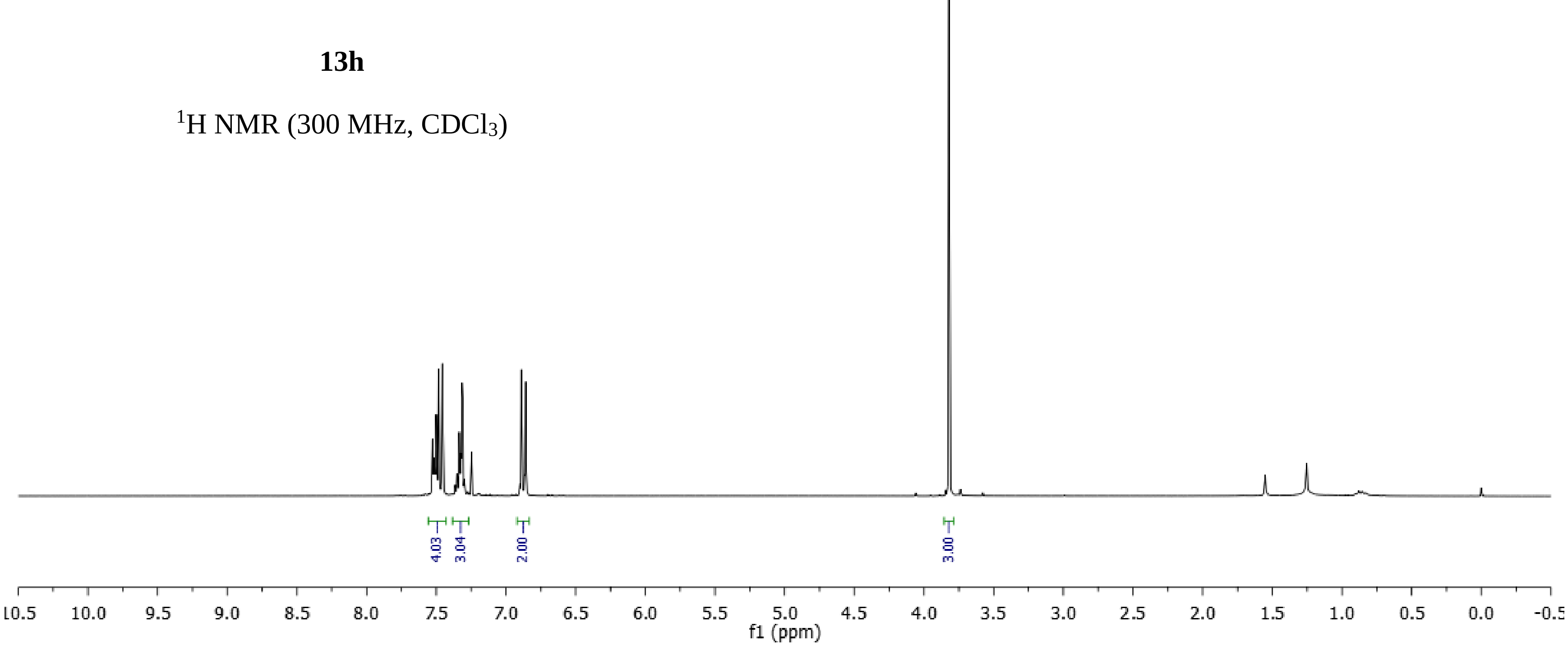

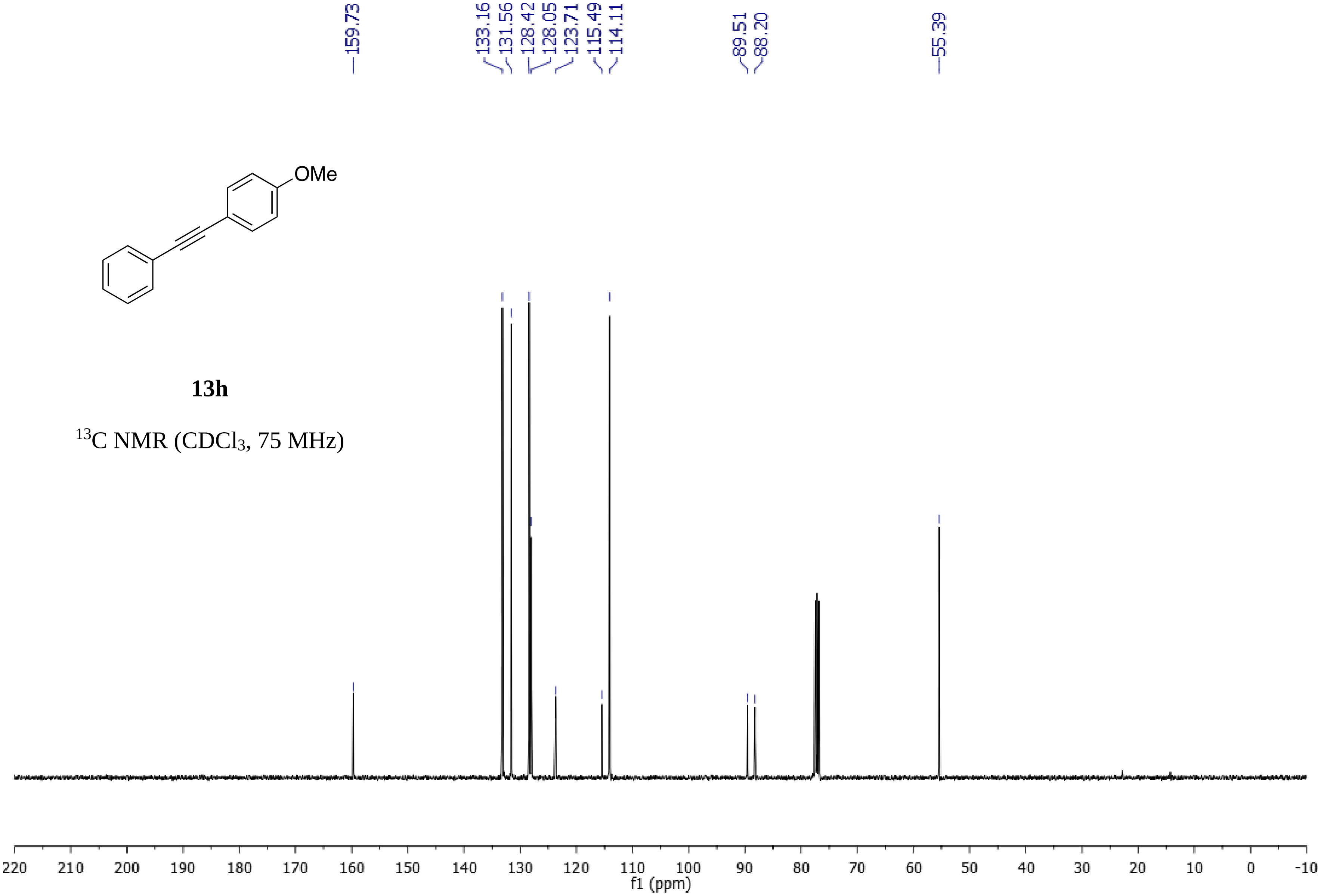

13h

^{13}C NMR ($CDCl_3$, 75 MHz)

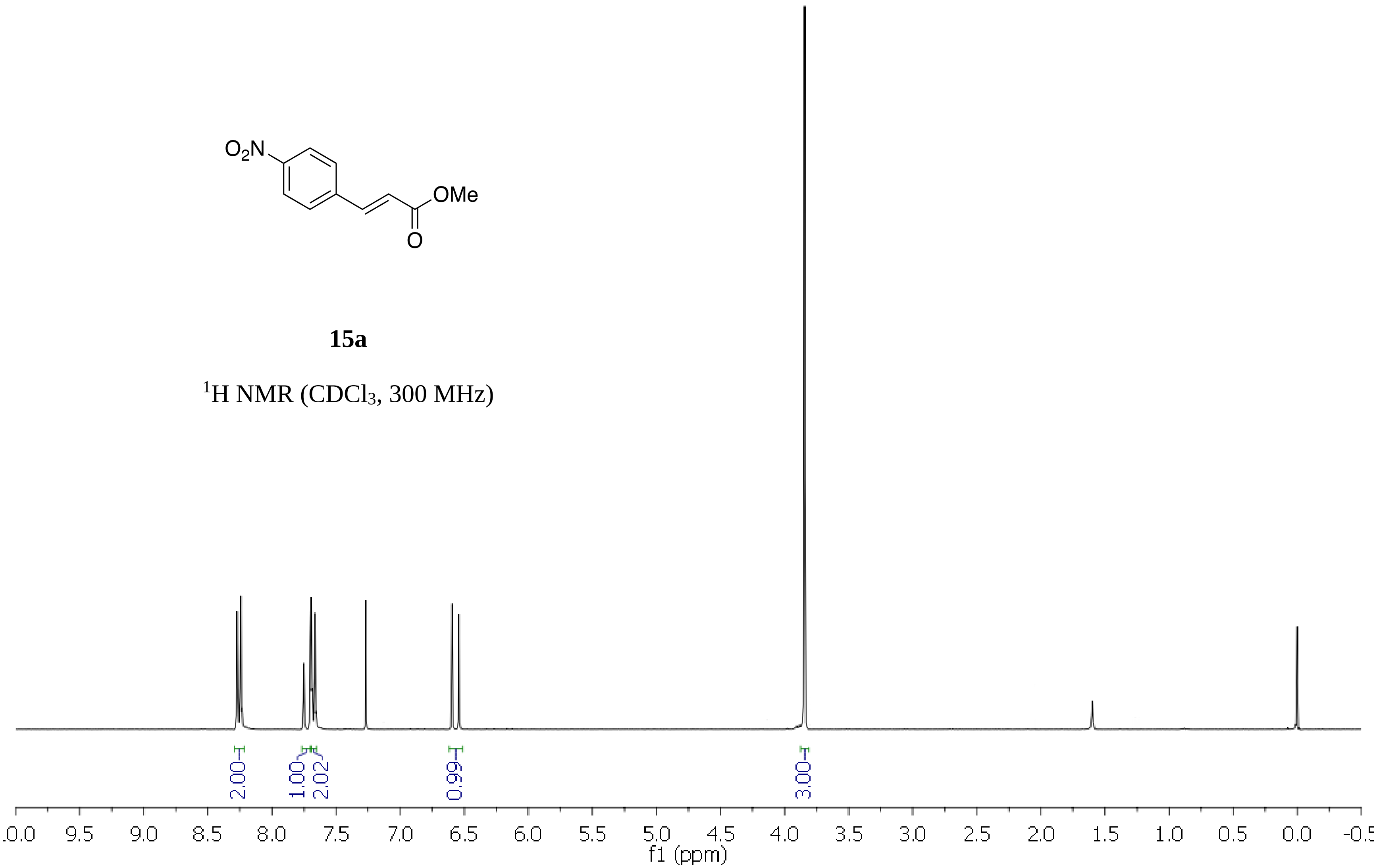

15a

^{1}H NMR ($CDCl_3$, 300 MHz)

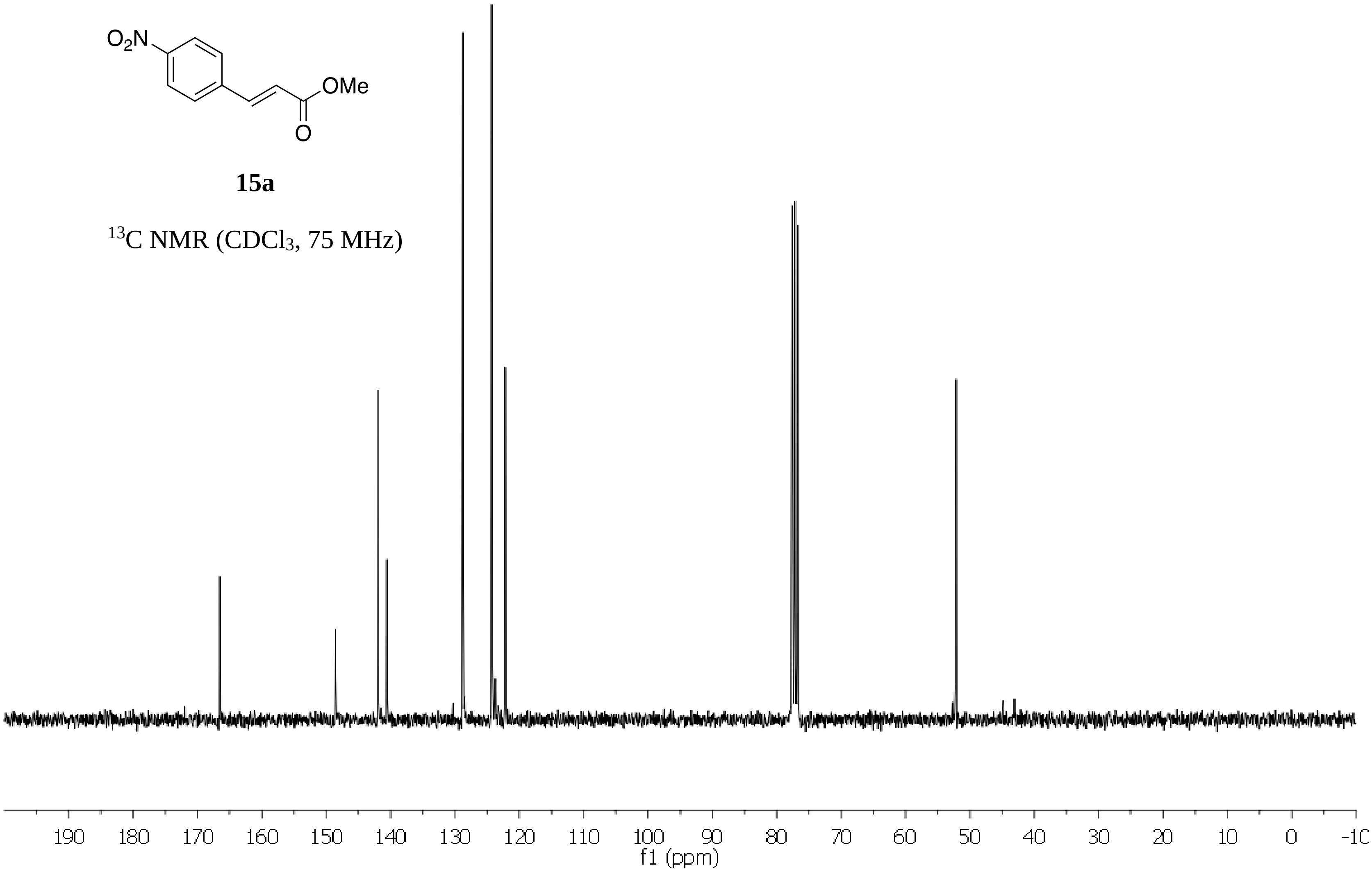

15a

^{13}C NMR ($CDCl_3$, 75 MHz)

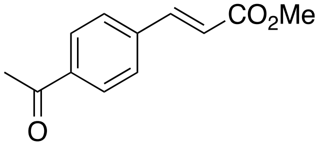

15b

^{1}H NMR ($CDCl_3$, 300 MHz)

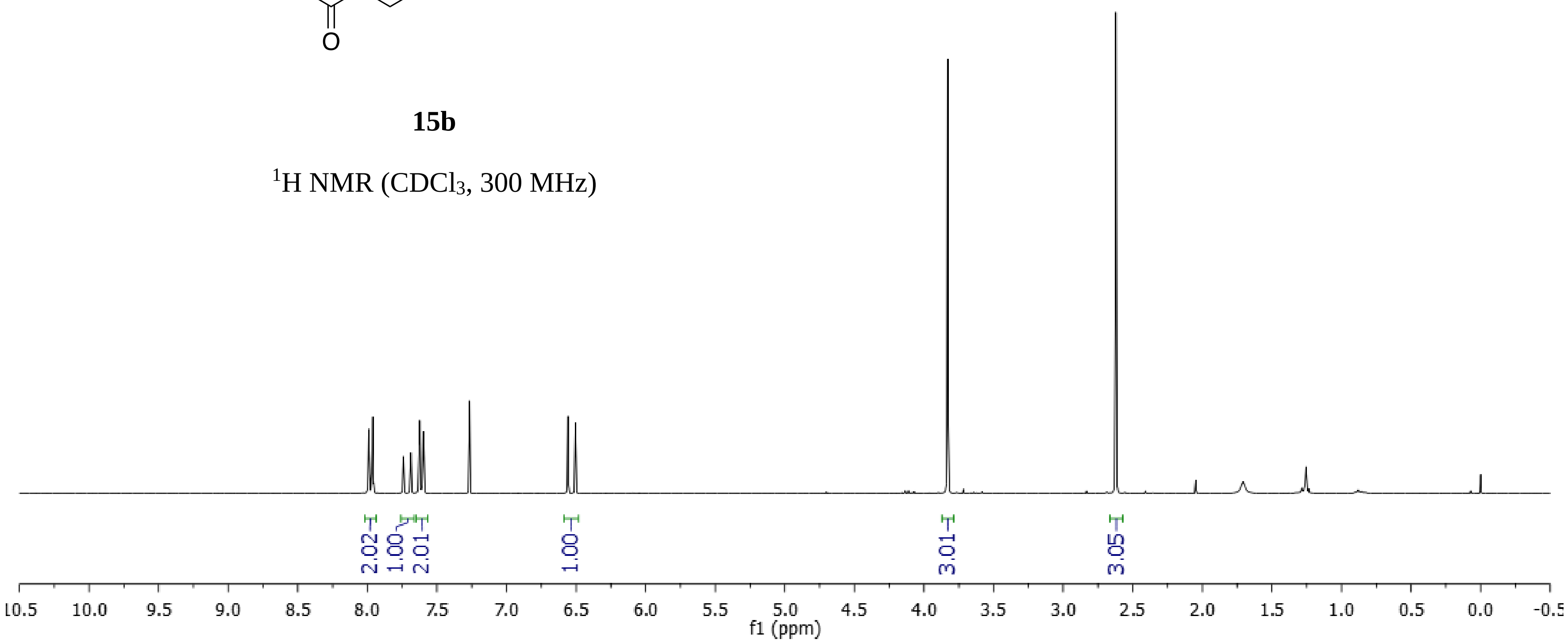

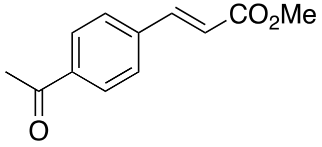

15b

^{13}C NMR ($CDCl_3$, 75 MHz)

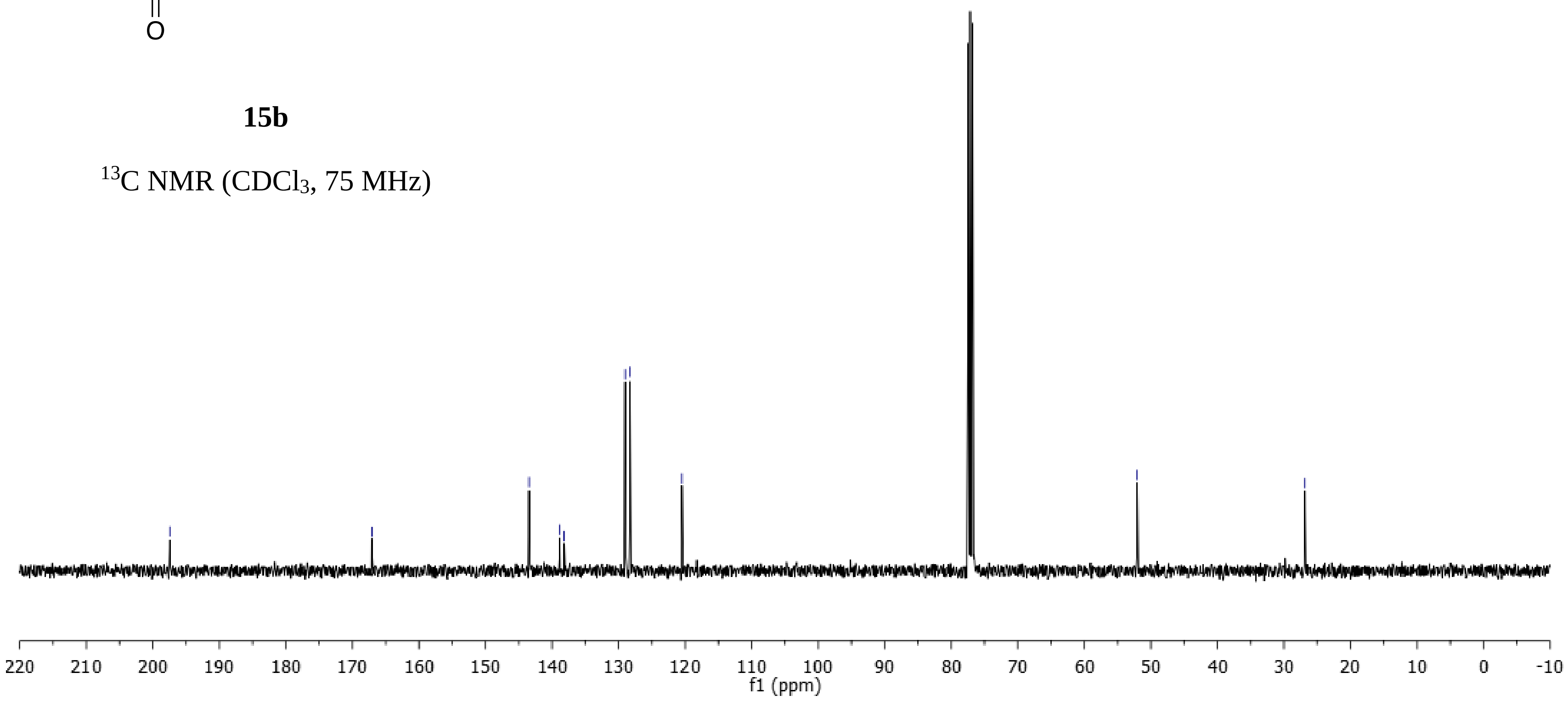

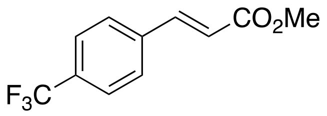

15c

^{1}H NMR ($CDCl_3$, 300 MHz)

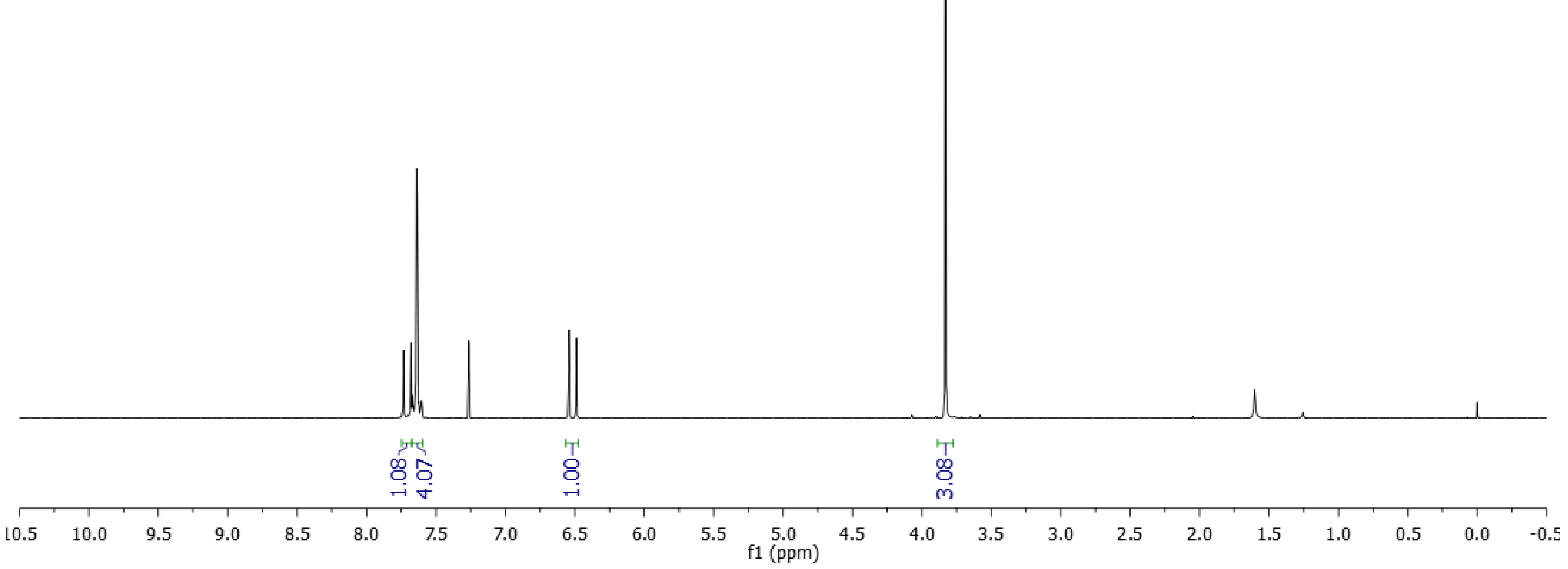

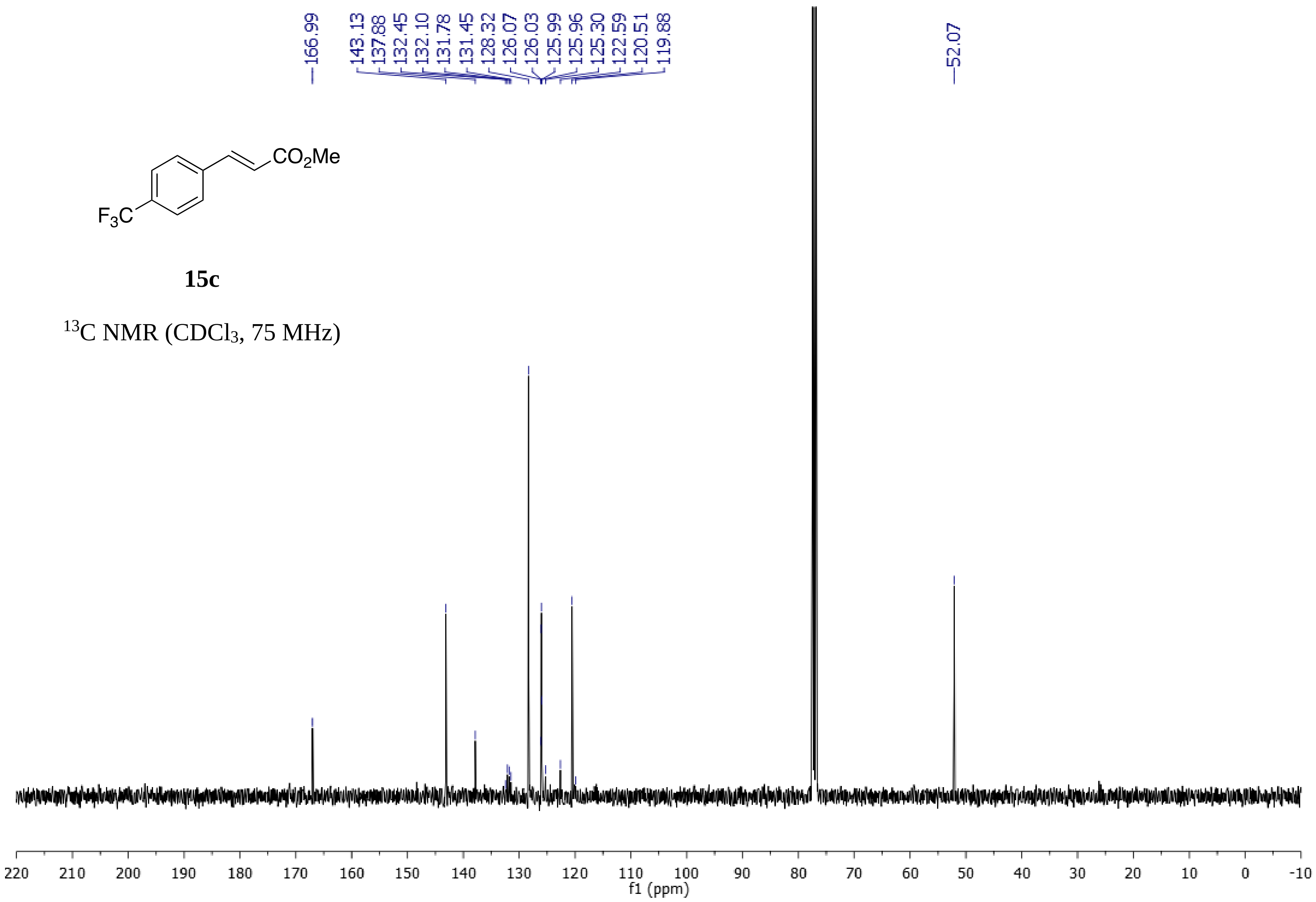

15c

^{13}C NMR ($CDCl_3$, 75 MHz)

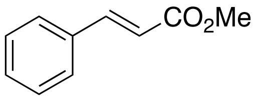

15d

^{1}H NMR ($CDCl_3$, 300 MHz)

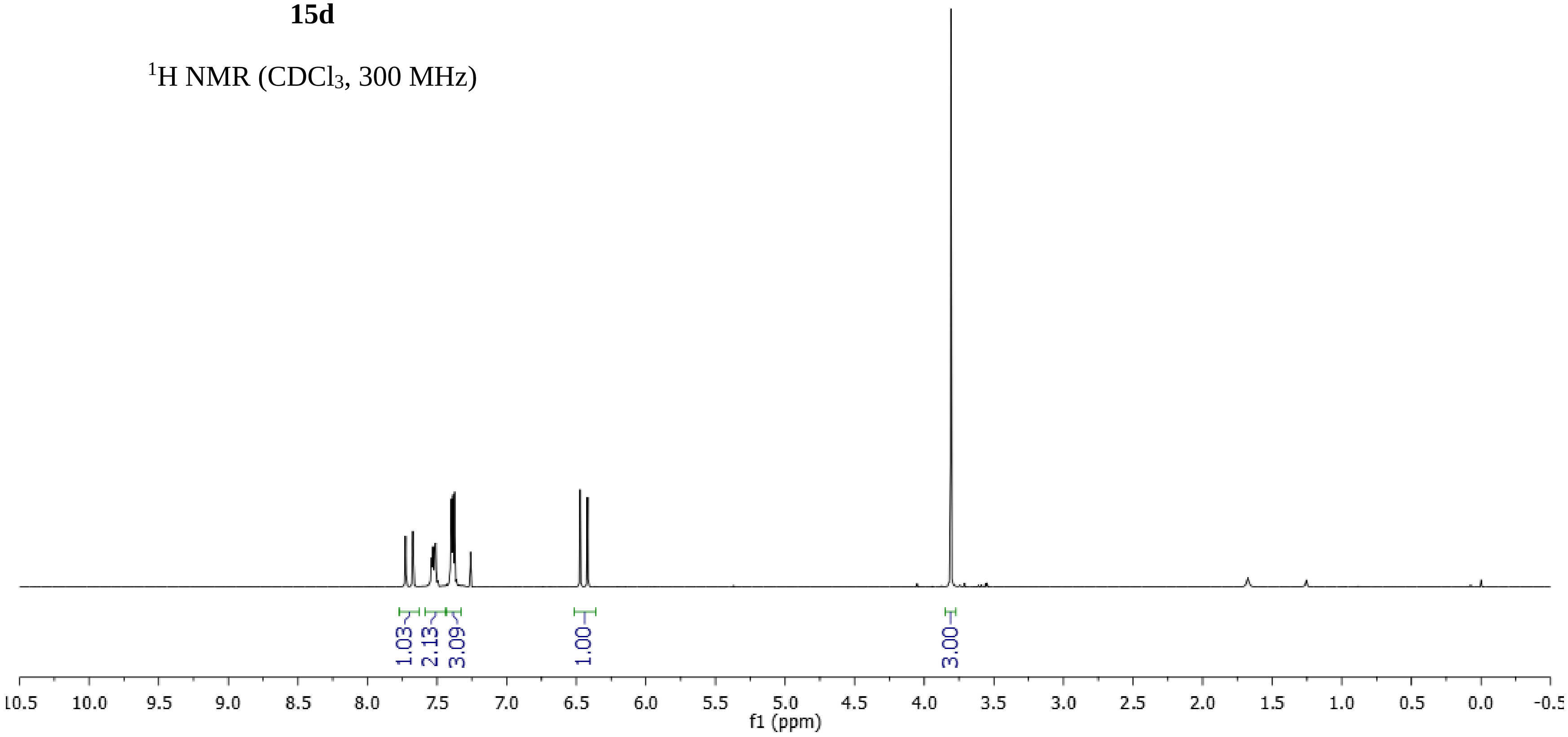

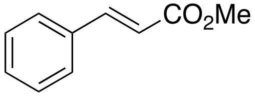

15d

^{13}C NMR ($CDCl_3$, 75 MHz)

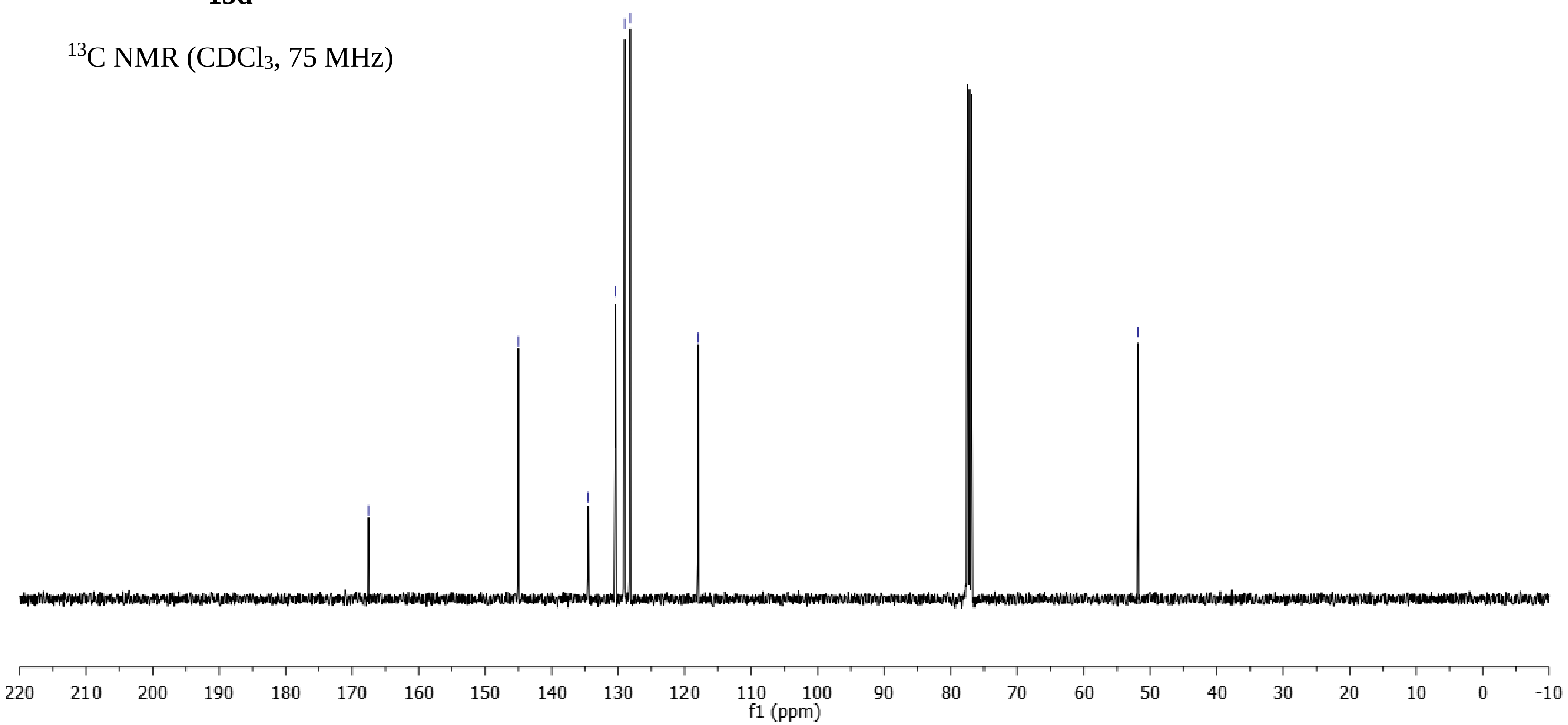

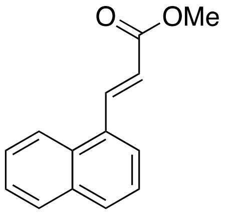

15e

^{1}H NMR ($CDCl_3$, 300 MHz)

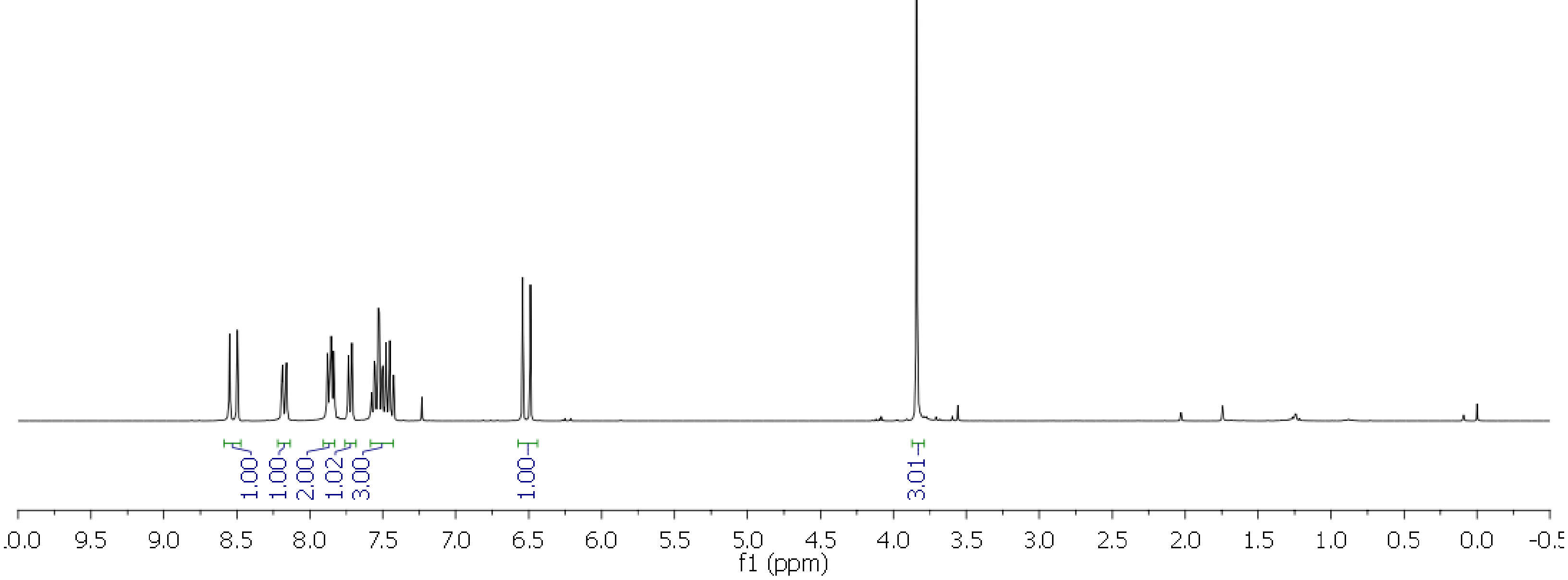

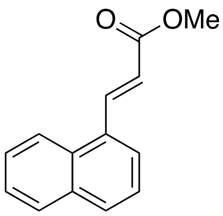

15e

^{13}C NMR ($CDCl_3$, 75 MHz)

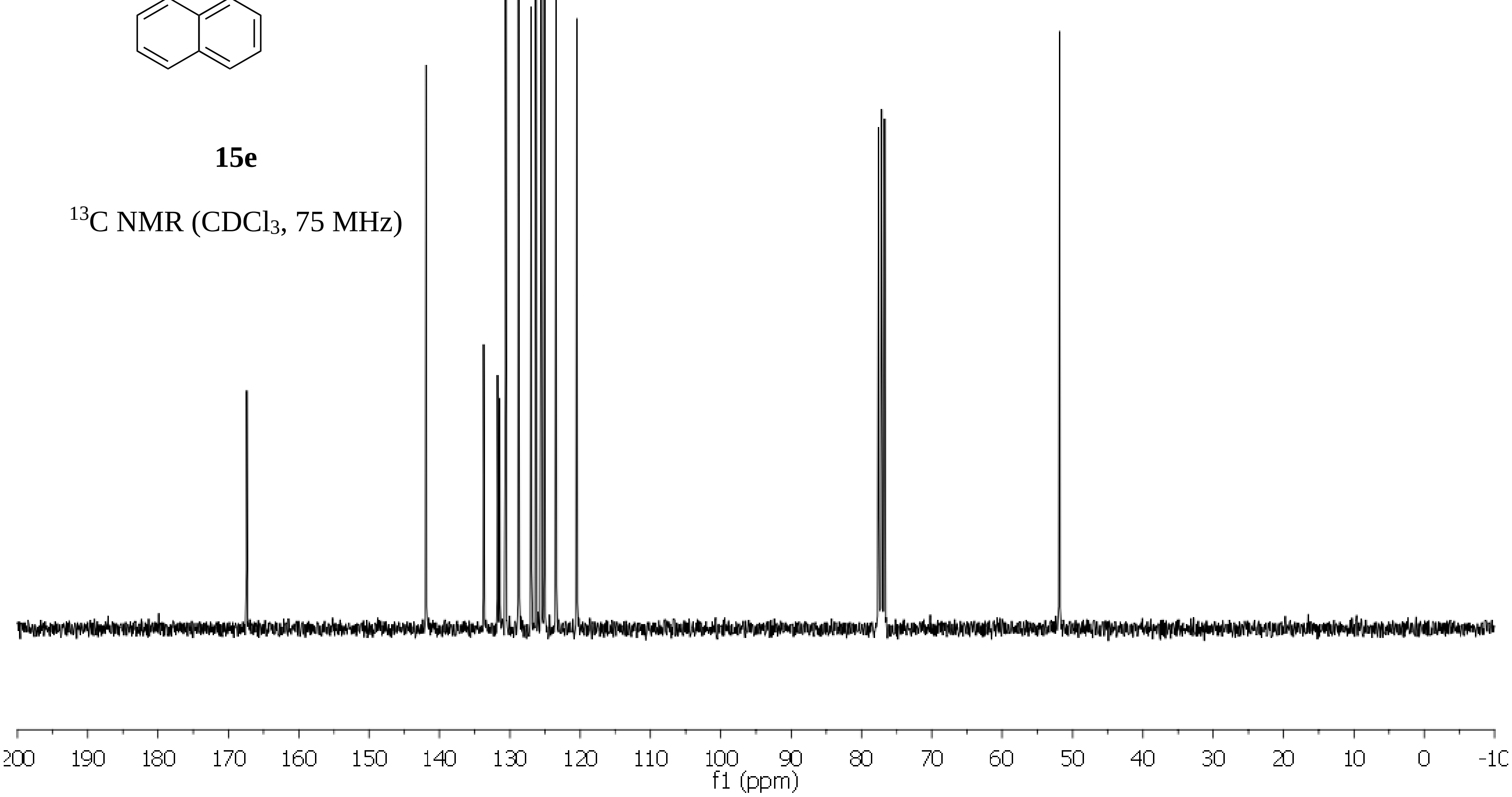

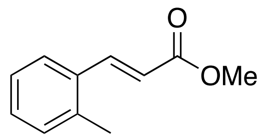

15f

^{1}H NMR ($CDCl_3$, 300 MHz)

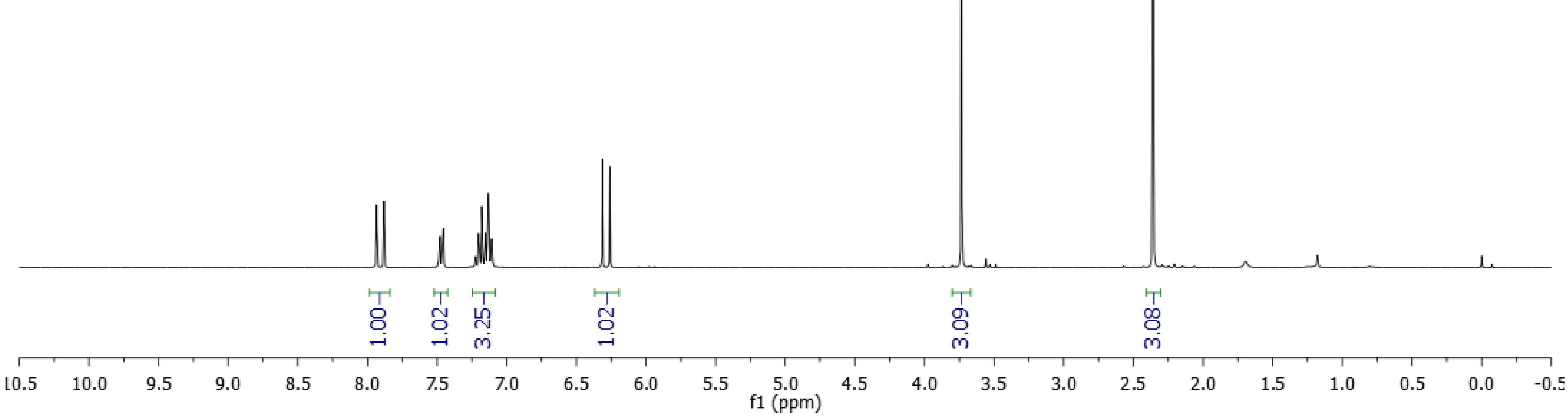

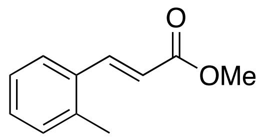

15f

^{13}C NMR ($CDCl_3$, 75 MHz)

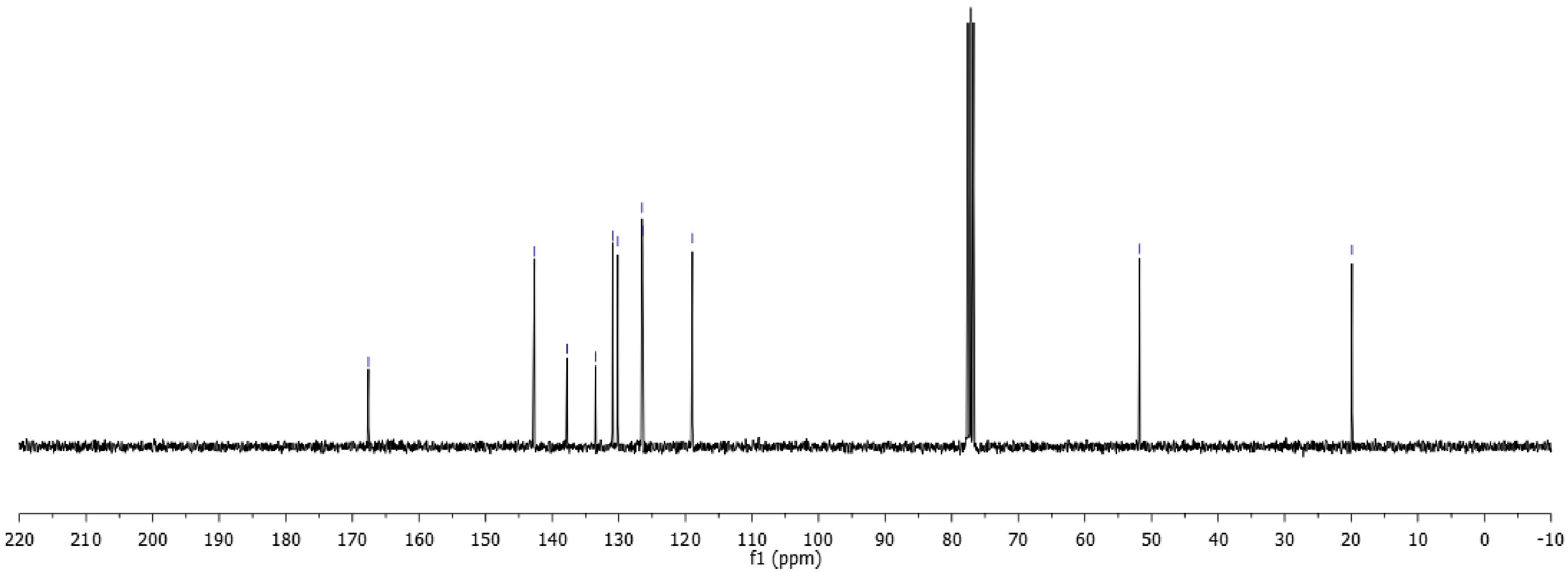

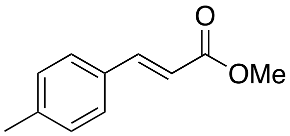

15g

^{1}H NMR ($CDCl_3$, 300 MHz)

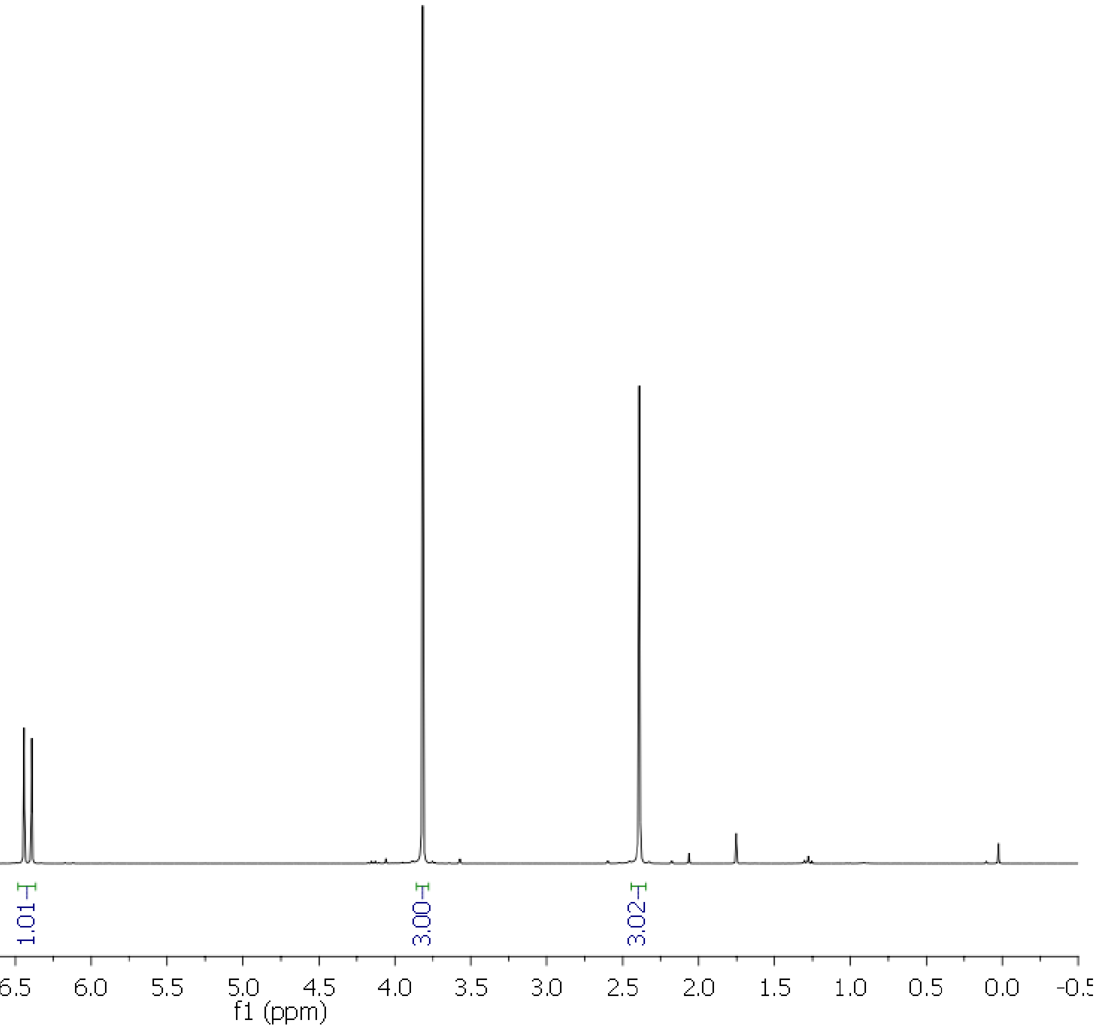

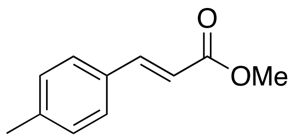

15g

^{13}C NMR ($CDCl_3$, 75 MHz)

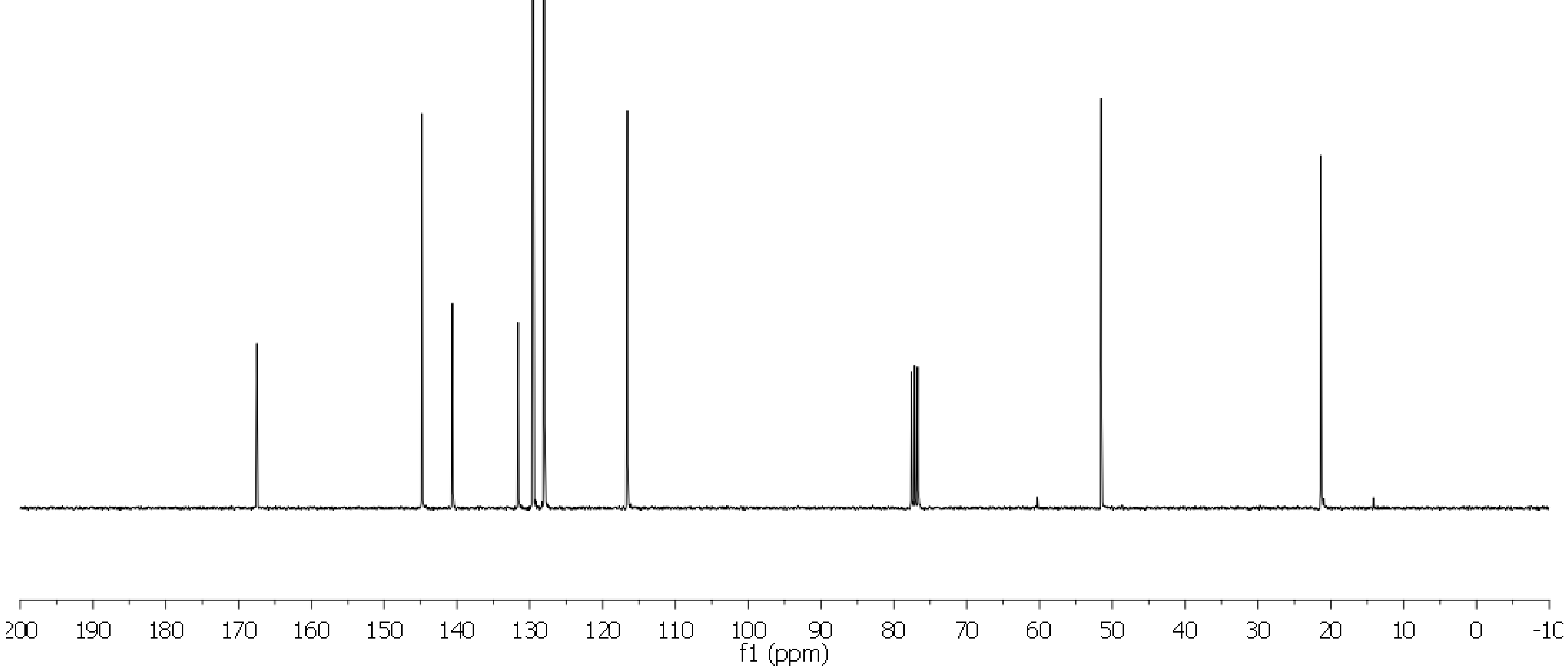

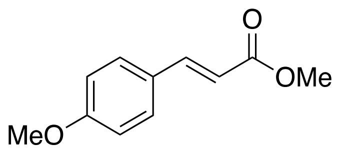

15h

^{1}H NMR ($CDCl_3$, 300 MHz)

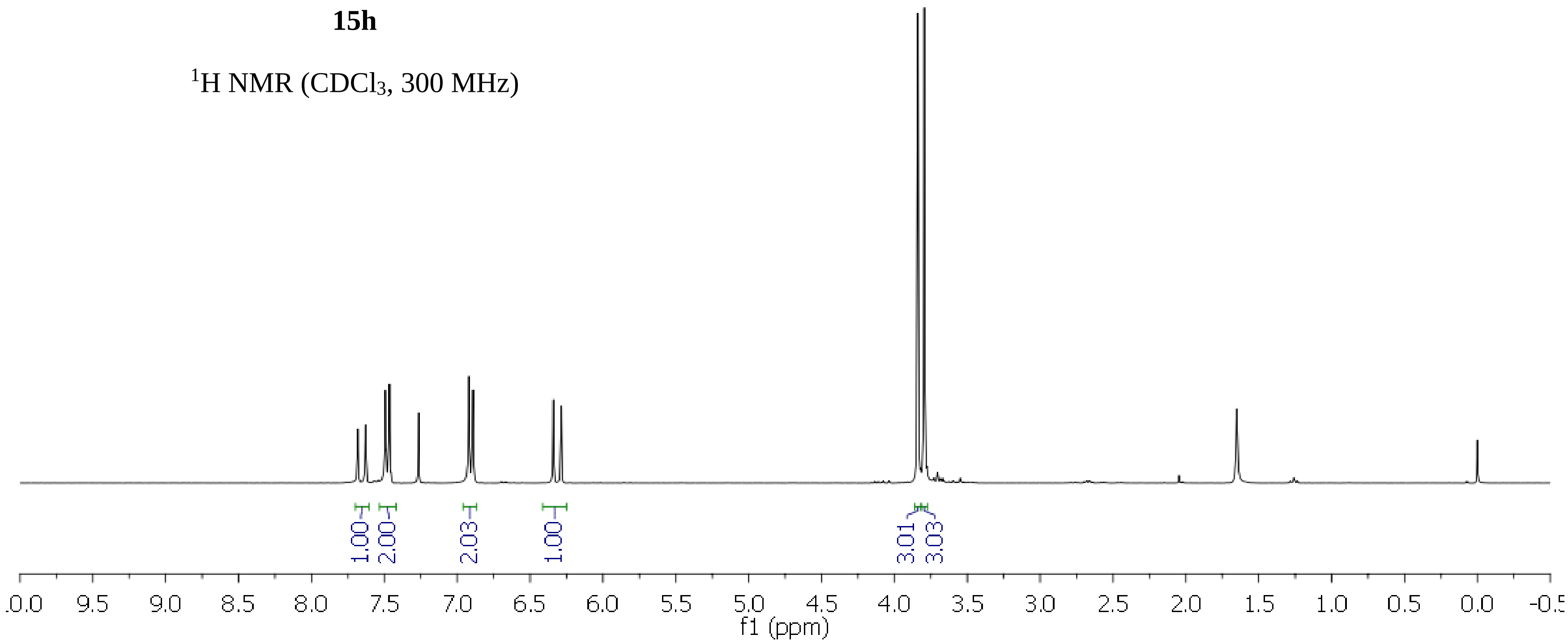

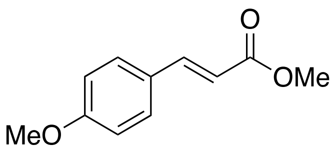

15h

^{13}C NMR ($CDCl_3$, 75 MHz)

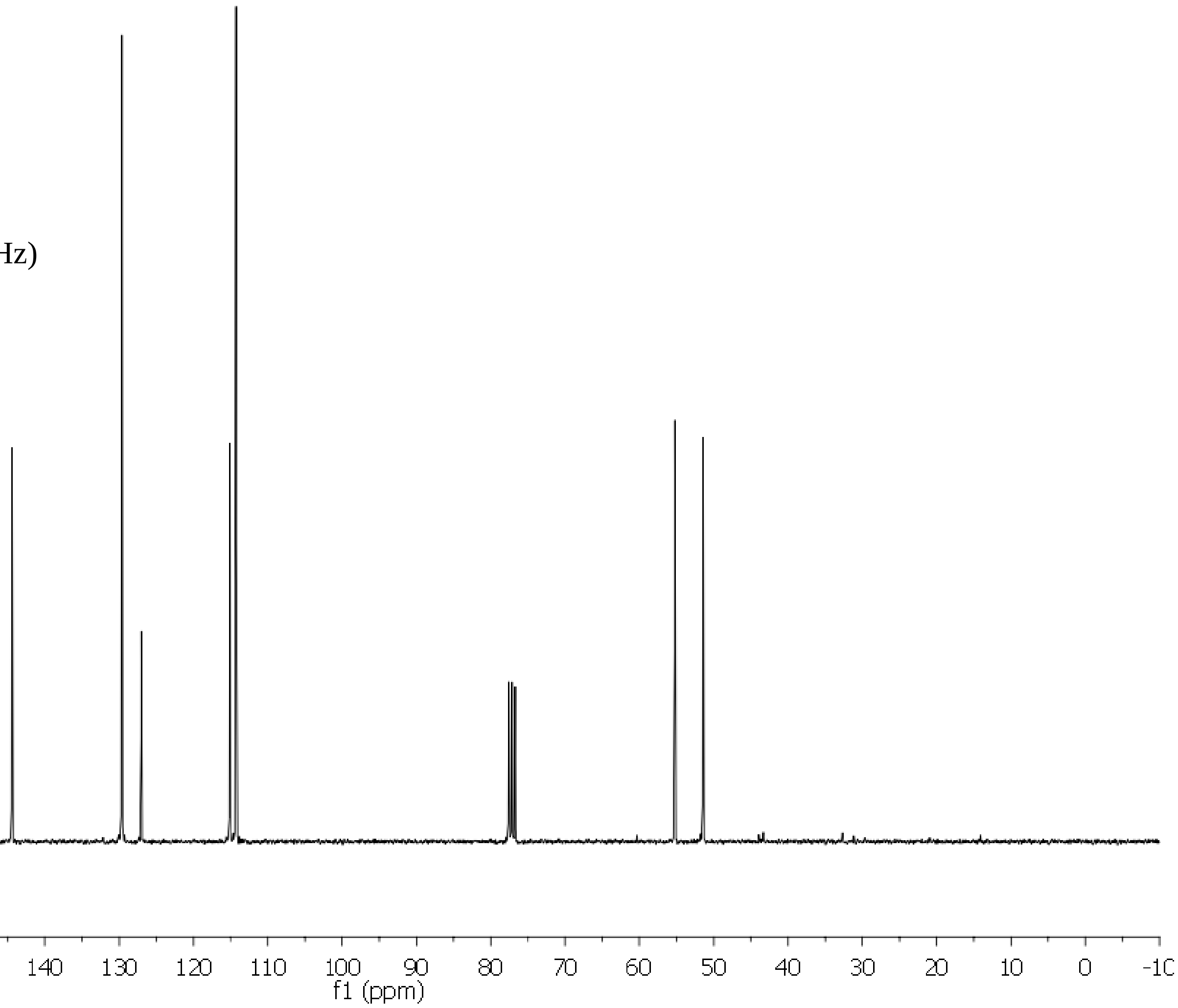

Supplement: Supplementary file 1 [file Data_Sheet_1.PDF]
